# *Stevia rebaudiana* Bertoni as a sweet herbal medicine: traditional uses, potential applications, and future development

**DOI:** 10.3389/fphar.2025.1638147

**Published:** 2025-09-04

**Authors:** Lisha Wang, Tianying Chang, Tonggang Zhu, Wenxin Hu, Xiaodan Wang, Chenxuan Dong, Yu Sun, Tianpeng Zhang, Yue Jiang, Chunna Zhao, Yingzi Cui, Jiajuan Guo, Xing Liao

**Affiliations:** ^1^ College of Traditional Chinese Medicine, Changchun University of Chinese Medicine, Changchun, China; ^2^ Affiliated Hospital of Changchun University of Chinese Medicine, Changchun, China; ^3^ Department of Rehabilitation, Jilin Provincial Electric Power Hospital, Changchun, China; ^4^ Institute of Basic Research in Clinical Medicine, China Academy of Chinese Medical Sciences, Beijing, China

**Keywords:** *Stevia rebaudiana* Bertoni, ethnobotany, traditional medicine applications, bioactivity, health promotion

## Abstract

*Stevia* *rebaudiana* Bertoni (*S. rebaudiana* for short), native to the Amambay Mountains of South America, is a sweet tasting medicinal plant with a long history of use in traditional medical systems. With the increasing global interest in natural products, their ethnopharmacological value and therapeutic potential have received growing attention from researchers, physicians, patients, and consumers. This review aims to comprehensively assess the ethnobotanical traits, traditional uses, pharmacological activities, major constituents, mechanisms of action, and safety profiles of *S. rebaudiana*. A comprehensive literature search was conducted using English and Chinese databases—Web of Science, PubMed, Scopus, ScienceDirect, CNKI, Google Scholar, and Elsevier—covering publications up to March 2025, including the keywords “ethnobotany”, “geographical distribution”, “traditional medicine”, “bioactivity”, “phytochemistry”, “pharmacological activities” and “toxicity”. Its taxonomic identity was confirmed using the Medicinal Plant Names Services (MPNS) and Plants of the World Online (POWO). Additional ethnopharmacological monographs and reference works were consulted to supplement database coverage. These findings show that *S. rebaudiana* has been traditionally used to lower blood glucose levels, reduce inflammation, and promote digestion. Modern research has identified bioactive metabolites, such as diterpenes, flavonoids, and phenolic acids, which exhibit antioxidant, anti-inflammatory, neuroprotective, and hepatoprotective effects. These properties have therapeutic potential for the management of metabolic disorders, cardiovascular diseases, neurodegenerative conditions and liver dysfunction. Although generally recognized as safe, some studies have reported immunological and reproductive concerns under high-dose or prolonged exposure, underscoring the need for further toxicological and clinical evaluation. In conclusion, this review bridges traditional ethnomedical knowledge with modern pharmacological evidence, providing a foundation for future research and the potential clinical translation of S. rebaudiana in phytotherapy.

## Graphical Abstract

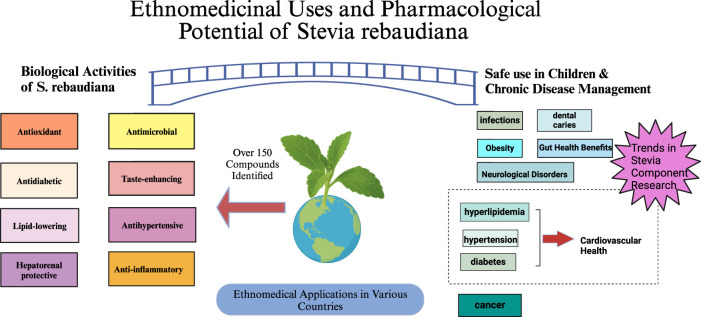

## Highlights

• This review summarizes the ethnomedical uses of *Stevia rebaudiana* across multiple traditional medical systems.
• The phytochemistry and biological activities of Stevia are comprehensively categorized and analyzed.
• This work bridges ethnopharmacological heritage and modern therapeutic potential of *Stevia rebaudiana*.

## 1 Introduction

*Stevia rebaudiana* Bertoni (*S. rebaudiana* for short) is a perennial herbaceous plant belonging to the Asteraceae family, native to the Amambay Mountains of South America, and primarily distributed in Paraguay and Brazil. Its sweetness is approximately 200–300 times that of sucrose, but it contains virtually no calories (Goyal et al., 2010). For centuries, indigenous Guaraní people have referred to it as “kaa-hee” (sweet herb), using it to sweeten yerba mate as well as to treat fever, manage diabetes, regulate blood pressure, combat microbial infections, and promote digestion (Marcinek and Krejpcio, 2016; Wu et al., 2021). It occupies a significant position in traditional South American medicine.

With the advancement of global trade and cultural exchanges, the unique sweetening properties and pharmacological potential of *S. rebaudiana* have garnered worldwide attention. The plant has been introduced into numerous countries and has gradually been integrated into various traditional ethnomedical systems, evolving into a widely used ethnobotanical resource. In the 1970s, *S*. *rebaudiana* was introduced to China, and commercial cultivation began in the 1980s. Since then, China has become the world’s largest producer and exporter of *S*. *rebaudiana* leaves and extracts (Brinckmann, 2015).

In traditional Chinese medicine (TCM), *S. rebaudiana*, also known as “sweet tea” is classified as entering the lung and stomach meridians, with functions of generating body fluids to quench thirst, promoting diuresis, and lowering blood pressure (Wang, 2000). Additionally, the need to improve the palatability of bitter herbal decoctions has led to the increased incorporation of *S. rebaudiana* in TCM. These factors have driven further investigation into the historical development and medicinal potential of *S. rebaudiana*, extending beyond its sweet taste to ensure its safe and effective integration into modern herbal medicine.

In recent years, extensive research has revealed that *S. rebaudiana* is not only a natural sweetener, but also a rich source of bioactive metabolites. To date, more than 153 active constituents have been isolated and identified from leaf extracts, including steviol glycosides, flavonoids, phenolic acids, diterpenoids, phenylethanoid glycosides, amino acids, fatty acids, glycerides, and oligosaccharides (Wölwer-Rieck, 2012; Molina-Calle et al., 2017; Prakash et al., 2017; Samuel et al., 2018; He et al., 2019; Myint et al., 2020; Kang et al., 2022). The bioactive metabolites in *S. rebaudiana* contribute not only to its sweetness but also to a range of therapeutic effects, including antidiabetic, anti-inflammatory, antioxidant, neuroprotective, and immunomodulatory activities (Borgo et al., 2021). These properties highlight its potential for managing various health conditions, including chronic diseases and inflammatory disorders, and offer protective benefits for cardiovascular, hepatic, and renal functions (Bao et al., 2024).

Despite the growing body of research, the connection between the ethnobotanical properties, traditional uses, and modern pharmacological studies of *S. rebaudiana* has not been comprehensively reviewed, and its application varies significantly across different cultures and medical systems (Arumugam et al., 2020). To date, no comprehensive review has elucidated its cross-cultural- therapeutic practices, bioactive mechanisms, and safety considerations.

Therefore, this review aims to comprehensively summarize the ethnobotanical background and traditional usage experience of *S. rebaudiana* across various traditional medical systems, explore its pharmacological activity and mechanisms of action, and critically analyze its safety. Understanding the historical context of *Stevia* not only enriches its cultural significance but also provides a foundation for its integration into modern healthcare solutions.

## 2 Methodology

### 2.1 Literature search strategy

A comprehensive literature search was conducted to collect relevant information on the ethnopharmacology, geographical distribution, traditional medicine, bioactivity, phytochemistry, pharmacology, and toxicology of *Stevia*. This process involved reviewing a wide range of sources, including articles, book chapters, books, and encyclopedias written in English and supported by scientific research projects and funds, within the time span from the inception of various databases to March 2025.

### 2.2 Databases searched

The literature search was conducted in several major scientific electronic databases to ensure comprehensive coverage of the subject. Relevant articles, book excerpts, books, and encyclopedias were extensively searched in Web of Science, PubMed, Scopus, ScienceDirect, Elsevier, Google Scholar, and CNKI, within the time frame from their inception to March 2025. Each database was searched using combinations of the identified keywords, and the results were reviewed to identify studies and materials that were consistent with the research objectives.

### 2.3 Inclusion criteria

The following criteria were set to select premium literature: publications written in English, studies supported by scientific evidence, research aligning with the focus on ethnopharmacology, phytochemistry, pharmacology, and toxicology, with a preference given to up-to-date studies, though some earlier works were also included when deemed to be groundbreaking in this respect.

### 2.4 Data extraction

Key data were extracted from the selected literature, focusing on the following: Ethnopharmacology (traditional medicinal uses of plant taxa), Geographical distribution (regions where the plant is native or commonly found), phytochemistry (identification and analysis of chemical metabolites focusing on the following scopes: ethnopharmacology (traditional medicinal uses of plant taxa), its geographical distributions (regions where the plant is native or commonly found), phytochemistry (identification and analysis of its chemical metabolites found in plant species), pharmacological effects (recorded therapeutic benefits and bioactivity), and toxicological properties (information on its safety, side effects, and toxicological studies).

### 2.5 Structural identification of phytochemicals

ChemDraw^®^ was used to display the chemical structures of the metabolites identified in the phytochemical analyses of *S*. *rebaudiana*. This allows for clear visualization of its molecular structures and ensures an accurate representation of its phytochemical data.2.6 Data Analysis and Synthesis.

After extraction, the data were organized and synthesized to gain a clear understanding of the plant’s ethnopharmacological relevance and its chemical composition. A comparative analysis was also conducted to correlate the phytochemical findings with pharmacological and toxicological results, taking a comprehensive approach to understand the medicinal value of *S. rebaudiana*.

## 3 Ethnobotanical background of *Stevia* Cav.

*Stevia* Cav. (family: Asteraceae, subfamily: Eupatoriae) is native to South America, with its distribution primarily in Paraguay, Brazil, and Argentina (Wu et al., 2021). The plant thrives in warm, humid, and subtropical climates and is commonly found in well-drained mountainous regions or moist grasslands (Bhattacharjee et al., 2020). Its natural distribution extends from the southwestern United States to northern Argentina, encompassing Mexico, the Andes, and the Brazilian Highlands (Brahmachari et al., 2010).

To date, the *Stevia* genus comprises 269 recognized species (https://powo.science.kew.org/, last accessed on 4 March 2025). Based on taxonomic studies, the *Stevia* species in North and Central America are grouped into three clades: *podocephalae*, *corymbosae*, and *fruticosae*, while the South American species are classified into the *breviaristatae* and *multiaristatae* groups (Brahmachari et al., 2010). Among them, *S. rebaudiana* and 14 other species are believed to have originated in Paraguay, which is also home to an additional 17 related species (Brahmachari et al., 2010). Table 1 summarizes the botanical classification and morphological characteristics of *S*. *rebaudiana*.

**TABLE 1:** Botanical taxonomy and morphological characteristics of *S. rebaudiana.*

Category	Taxonomic level	Classification
Botanical taxonomy	Kingdom	Plantae
	Subkingdom	Tracheobionta
	Superdivision	Sprematophyta
	Division	Magnoliophyta
	Class	Magnoliopside
	Subclass	Asteridae
	Cohort	Monochlamydae
	Order	Asterales
	Family	Asteraceae (formerly Compositae)
	Subfamily	Asteroideae
	Tribe	Eupatorieae
	Genus	*Stevia*
	Species	*rebaudiana*
Morphological Traits	Leaf	Petiole: Present or absent; Shape: Linear, oblong, elliptical, ovate, rhomboid; Margin: Entire, subentire, rounded-toothed, serrated, dentate; Glands: Glandular hairs present
	Flower	Pedicel: Short-pedicellate or sessile; Corolla: Funnel-shaped; Involucre: Cylindrical; Number: 5 to 6 florets per capitulum; Color: White, pink, or purple

Morphologically, *S. rebaudiana* features an erect or semi-prostrate growth habit, reaching a height of approximately 50–120 cm. It has upright stems and variable leaf shapes, which are typically lanceolate, elliptical, or serrated. The smooth and waxy leaf surfaces help reduce water loss and enable the plant to adapt to arid environments (Soejarto et al., 1982). Its inflorescences are small capitula bearing white or pale purple flowers, which are pollinated by both wind and insects. Following pollination, the plant produces small achenes equipped with feathery pappus structures, which are helpful for seed dispersal. These adaptive traits have facilitated the successful cultivation of this species in diverse ecological environments across the globe (Ramesh et al., 2006).

In several indigenous communities in Paraguay and Brazil, *Stevia* species—particularly *S. rebaudiana*—are well-known for their leaves, which contain high concentrations of steviol glycosides, the metabolite responsible for the intense sweetness of the plant (Borgo et al., 2021). Taxonomically, *S. rebaudiana* has been referred to as *Eupatorium rebaudianum* Bertoni and *Stevia rebaudiana* Bertoni Hemsl.

Owing to its unique sweetening properties and medicinal potential, *S. rebaudiana* has been introduced and cultivated in numerous countries across Asia and Europe, including China, Thailand, Bangladesh, India, Java, New South Wales, Sri Lanka, Switzerland, the Caucasus, and the western Himalayas (https://powo.science.kew.org/, last accessed on 4 March 2025). In recent years, China has become one of the leading cultivators of *S. rebaudiana*, with significant production regions including Beijing, Hebei, Shaanxi, Jiangsu, Fujian, Hunan, and Yunnan (Wang, 2000).

**FIGURE 1:**
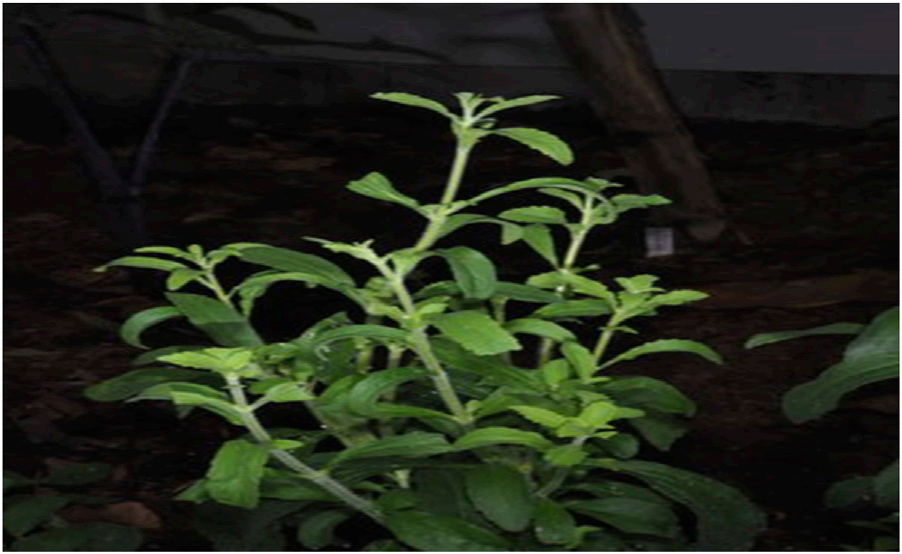
Nutritional growth stage whole plant diagram showingerect stems with opposite leaves (POWO, 2025).

**FIGURE 2:**
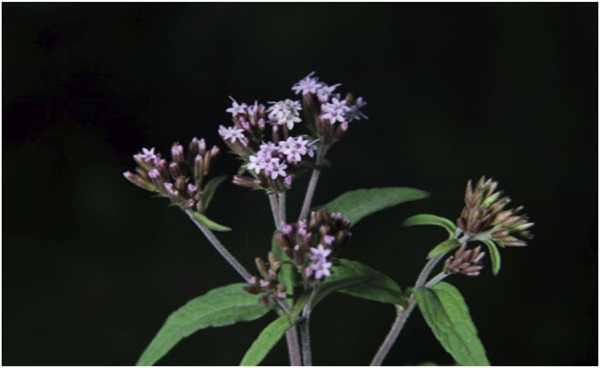
Inforescences with pinkish-purple flowers (POWO, 2025).

**FIGURE 3:**
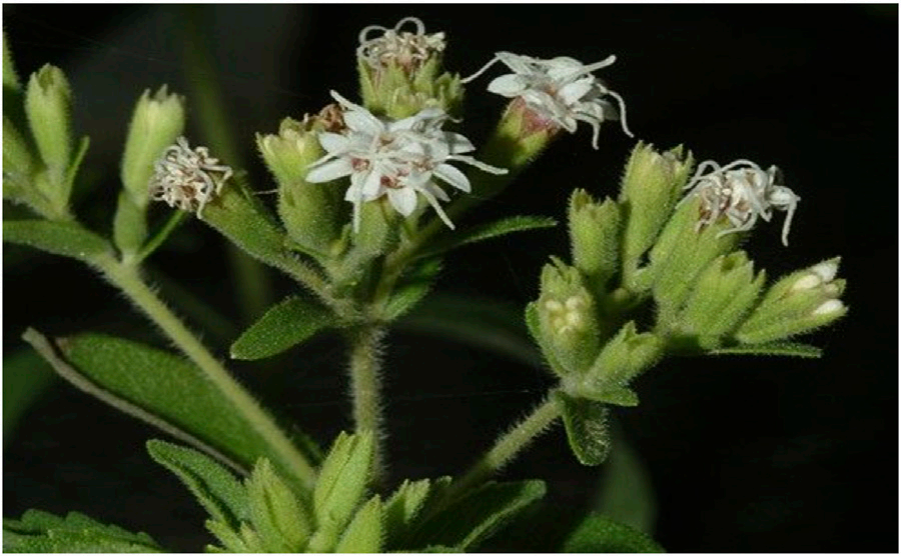
Inflorescence of green bracts with unopened white flowers (POWO, 2025).

**FIGURE 4:**
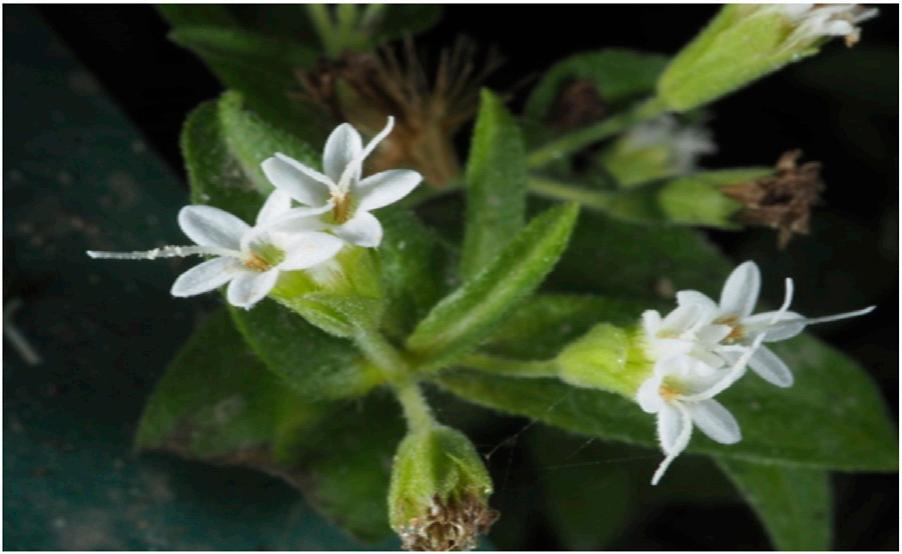
Close up of mature white flower showing corolla and stigma (POWO, 2025).

From an ethnobotanical perspective, the global spread of *S*. *rebaudiana* reflects not only the diverse ways in which varying cultures utilize the plant but also the intersection of traditional medicine and modern health demands. As a culturally significant medicinal plant with cross-cultural value, the diverse perceptions and uses of *S. rebaudiana* across nations offer a rich ethnobotanical context for understanding its global development and integration into both traditional and modern healthcare systems. The botanical taxonomy and morphological characteristics of *S. rebaudiana* are presented in Table 1, while representative morphological features are illustrated in Figures 1–4 (images adapted from Plants of the World Online, POWO: https://powo.science.kew.org/, accessed 4 March 2025).

## 4 Ethnomedical applications and development potential of *stevia* species

Since ancient times, plants have been widely utilized for the treatment of various ailments and are regarded as natural and effective sources of medicine (Chen and Ye, 2022). Among the species within the *Stevia* genus, *S. rebaudiana* stands out as the only one that has been extensively employed in ethnomedicine (Marcinek and Krejpcio, 2016).

In traditional medical systems, the leaves of *S. rebaudiana* are considered the most therapeutically valuable part of the plant. They are commonly used in the form of decoctions, infusions, crude extracts, and polyherbal formulations (as outlined in Table 2), particularly for regulating blood glucose, reducing blood pressure, exhibiting anti-inflammatory effects, promoting diuresis, and providing digestive support. Modern pharmacological studies have partially validated these traditional uses, further justifying their potential as globally relevant medicinal resources (Chan et al., 2000; Chowdhury et al., 2022).

**TABLE 2:** Traditional uses of *S*. *rebaudiana*.

Common name(s)	Region	Uses Part(s)	Preparation method	Traditional uses	Ref.
Ka’a He’e, Hierba dulce, del Paraguay, estevia, stevia	Paraguay	Leaves	Decoction	Natural sweetener; treatment of stomachache and heartburn; management of hypertension and diabetes; preventive therapy; consumed as a general tonic; added to yerba mate; mild neurostimulant; hepatoprotective; contraceptive; diuretic	(Planas and Kucacute, 1968; Small et al., 2001; Marcinek and Krejpcio, 2016; Kujawska, 2018; de Souza et al., 2020; Kujawska and Schmeda-Hirschmann, 2022)
			Alcohol maceration	Orally administered in small doses to stabilize palpitations; enhance cardiac and circulatory functions; regulate blood pressure	Marcinek and Krejpcio (2016)
			Topical application	Treatment of minor skin abrasions, burns, and skin infections	(Small et al., 2001; Brahmachari et al., 2010)
		Leaves and stems	--	Antidiabetic; antihypertensive, anticancer; anticariogenic; anti-inflammatory; antimicrobial effects	Paiva et al. (2024)
*Esteviah, Caá-ehé* *Hierba dulce, del Paraguay, stevia*	Brazil	Leaves	Traditionally consumed in combination with yerba mate to enhance vitality and improve digestion	Used to relieve fatigue; as a tonic; for anticariogenic; antidepressant; antidiabetic, anti-fatigue; cardiotonic; antihypertensive; anti-infective; anti-obesity, diuretic; wound-healing purposes; anticancer; anti-inflammatory; antimicrobial effects; as a contraceptive; reduce cravings for sweet foods; as a natural sweetener	(Taylor, 2005; de Souza et al., 2020; Borgo et al., 2021; Paiva et al., 2024)
			Oral administration	Treatment of leishmaniasis and Chagas disease; trypanocidal activity	(Beer et al., 2016; Moraes Neto et al., 2019)
			Leaf extract infusion	Used as an antiinfective agent with antifungal and anti-yeast activities	Duarte et al. (2005)
Honey leaf, or sweet-leaf	Argentina	Leaves	Oral administration	Lowering blood glucose, cholesterol, and lipid levels; managing dyslipidemia and atherosclerosis; as diuretic, cardiotonic, and antihypertensive agent; relieving cough and heartburn; antacid, appetite stimulant, digestive aid, antidiarrheal, and mild laxative; weight management; promoting wound healing, moisturizing skin, and preventing dermatitis and aging; anticariogenic, contraceptive, antibacterial, antiviral, antiparasitic; immune enhancement and preventive healthcare	(Hurrell and Puentes, 2013; Hurrell et al., 2015; Kujawska, 2018; Cevasco Contreras et al., 2024)
Madhu Patrika or “Mou Tulsi” or “Cheeni Tulsi”	India	Leaves	Chewed directly or prepared as a saline mouthwash	Used for oral hygiene and prevention of dental caries; regular chewing of the leaves to help manage diabetes	(Pradeep et al., 2024; Balkrishna et al., 2025)
			Making tea	Used as a substitute for sugar-sweetened beverages to reduce caloric intake and manage obesity; prepared as a cooling herbal tea; used for the treatment of gastrointestinal discomfort	Ali et al. (2024)
			Decoction	Used to regulate blood pressure and enhance cardiac and circulatory function; for the treatment of diabetes and tuberculosis; Exhibiting non-carcinogenic; antioxidant, anti-inflammatory; antimicrobial; antidiarrheal; diuretic; immunomodulatory activities	(Das, 2013; Ali et al., 2024; Gorain et al., 2024; Manthattil Vysyan et al., 2024; Sharma and Alam, 2024)
			Topical application	Promoting wound healing	Manthattil Vysyan et al. (2024)
--	Columbia	Leaves	Taken on an empty stomach	Anthelmintic	(Beer et al., 2016; Moraes Neto et al., 2019)
--	America	Leaves	Ethanol extract (oral)	Used for weight loss and lipid-lowering; as a natural sweetener; as a hypoglycemic, antihypertensive, and vasodilatory agent; for treating infections, including *Candida* albicans infections; as a taste-enhancing agent	Mendoza-Pérez et al. (2024)
Sutebia	Japan	Leaves	Oral administration	Used as a substitute for saccharin and widely applied in tea beverages; pickled foods; seafood products; confectionery	(Goyal et al., 2010; Samuel et al., 2018)
		Stems		Stem extracts exhibiting spasmolytic activity; exerting their effects via the calcium channel blocking mechanism	Shiozaki et al. (2006)
Candyleaf	China	Leaves	Oral administration	Sweet and cooling in nature; traditionally used to moisten dryness; generate fluids; relieve thirst; acting as an adjuvant in managing Xiaoke syndrome (diabetes); hypertension; hyperuricemia; used in epilepsy management; improving taste of bitter herbal prescriptions to enhance compliance in pediatric patients	(Wang, 2000; Ding et al., 2016; Bai et al., 2024; Waris et al., 2024)
--	Russia	Leaves	Oral	As sweetener in food and beverages	Samuel et al. (2018)
Seuteibia	South Korea	Leaves	Extracts used in functional foods and cosmetic	Used as a natural ingredient in health foods and skincare products; believed to aid in lipid lowering; strengthen the intestinal mucus layer; reduce inflammation associated with pathogens	(Aran et al., 2014; Kim et al., 2023; Han et al., 2024)
หญ้าหวาน(Yaa Waan)	Thailand	Leaves	Oral	Commonly used in traditional herbal teas to regulate metabolic balance	Tipduangta et al. (2019)
			Extracts in skincare	Applied in the management of diabetes and obesity; extracts also used in skincare for antioxidant; anti-aging; anti-inflammatory; whitening effects	Chaiyana et al. (2021)
--	Indonesia	Leaves	Oral (tea with roselle flowers)	Used as a natural sweetener; herbal tea metabolite to lower blood glucose levels and manage diabetes	(Mayasari et al., 2018; Rahman et al., 2021)
Daun Manish or Gula Daun	Malaysia	Leaves	Oral	Natural sweetener	Samuel et al. (2018)
--	Bangladesh	Leaves	Oral (chewed raw or juiced with lime)	Used to reduce blood glucose levels	Rahman et al. (2021)
--	Australia	Leaves	Oral	Natural sweetener	Samuel et al. (2018)
--	New Zealand	Leaves	Oral	Natural sweetener	Samuel et al. (2018)
--	Mexico	Leaves		For skin bumps	Esquivel-García et al. (2018)
--	Ukraine	Leaves	Oral	Used to lower blood glucose levels	Marchyshyn et al. (2023)
--	Poland	Leaves	Oral	Used to support heart health	Olas (2022)
Folium Steviae	Vietnam	Leaves	Oral	Used to support heart health	Borgo et al. (2021)

### 4.1 Applications of *S. rebaudiana* in global traditional medical systems

#### 4.1.1 Systematic use in South America, the region of origin

South America, notably Paraguay, Brazil, and Argentina, is the native region of *S. rebaudiana* and represents the area with the richest ethnomedical knowledge related to this species. Its therapeutic applications encompass metabolic disorders, cardiovascular diseases, gastrointestinal ailments, infectious diseases, neuroprotection and interventions for reproductive system-related conditions (Planas and Kucacute, 1968; Small et al., 2001; Marcinek and Krejpcio, 2016).

In Guaraní traditional medicine, *S. rebaudiana* is referred to as “kaa-hee” (sweet herb). Its uses include blood glucose regulation, fatigue relief, digestive promotion, and antimicrobial defense (Marcinek and Krejpcio, 2016). It is also commonly consumed with the local herbal infusion*, yerba mate*, to purify the blood and promote general wellbeing (Kujawska, 2018). These culturally embedded applications suggest that *S. rebaudiana* functions not only as a therapeutic agent but also as a preventive supplement in daily life.

Built upon long-term experiential accumulation and traditional knowledge transmission, this plant is regarded as having broad therapeutic potential, including glycemic control (Kujawska, 2018), cardiovascular support (Kujawska and Schmeda-Hirschmann, 2022), hepatoprotective, diuretic effects, neuroprotection, anti-cariogenic, antimicrobial activities (Marcinek and Krejpcio, 2016; de Souza et al., 2020), antiparasitic action, antitussive effects (Taylor, 2005), and even potential contraceptive (Planas and Kucacute, 1968).

Notably, its role in reducing sugar cravings has been highlighted as an important dietary intervention for metabolic syndrome and weight management (Small et al., 2001). In Argentina, decoctions of *S. rebaudiana* leaves are also used in folk medicine to lower cholesterol levels, regulate blood lipids, reduce blood pressure and enhance cardiac function (Hurrell and Puentes, 2013; Hurrell et al., 2015; Kujawska, 2018; Cevasco Contreras et al., 2024).

In local practice, the applications of *S. rebaudiana* are diverse, encompassing both internal and topical forms. It is commonly believed that soaking the leaves in alcohol to prepare traditional medicinal tinctures enhances cardiovascular function (Planas and Kucacute, 1968). For external use, crushed fresh leaves are directly applied to the skin for treating minor wounds, burns, and infections (Small et al., 2001; Brahmachari et al., 2010). Pharmacological experiments have further demonstrated that its injectable extracts possess significant antifungal and anti-yeast activity (Duarte et al., 2005), indicating its potential value in treating infectious diseases.

Notably, against the historical backdrop of infectious disease prevalence, many South American countries are currently undergoing an epidemiological transition from acute communicable to chronic noncommunicable diseases, including type 2 diabetes, obesity, cardiovascular diseases, depression and oral health burdens (Carmo et al., 2018; Araya et al., 2021). Additionally, certain regions remain affected by parasitic (Echavarría et al., 2021; Pinto et al., 2023) and fungal infections and reproductive health issues (Alves et al., 2024). In resource-limited areas of South America (Danpanichkul et al., 2024), *S. rebaudiana*, a locally available, low-cost, and culturally accepted herbal resource, has significant practical value in both disease prevention and adjunctive therapy. Its extensive traditional applications provide a pragmatic foundation for primary public health interventions and offer critical support for future pharmacological research and clinical translation of its effects.

#### 4.1.2 Traditional use in North America

In North America, *S*. *rebaudiana* is primarily utilized as a natural sweetener in foods and beverages. It is widely regarded as beneficial for weight management, regulation of blood glucose and blood pressure levels, and promotion of gut health (Samuel et al., 2018). In the United States, *S*. *rebaudiana* leaves are also incorporated into certain folk herbal practices to enhance immune function and treat mild infections (Mendoza-Pérez et al., 2024). These applications are closely aligned with the rising burden of metabolic diseases currently observed in North America, particularly in the United States.

Additionally, in Mexico, *S*. *rebaudiana* leaves have been traditionally used to treat skin lumps and mild dermatological conditions (Esquivel-García et al., 2018). Given that some rural populations in this region have relatively limited access to healthcare services, leveraging indigenous plant resources for primary health interventions holds considerable practical significance (Alves et al., 2024).

Therefore, the traditional uses of *S. rebaudiana* in North America not only illustrate its cultural heritage but also signify its potential applications in regional public health services, warranting further in-depth research and clinical transformation within the framework of modern medicine.

#### 4.1.3 Functional expansion in asian medical systems

In Asia, *S*. *rebaudiana* has been increasingly integrated into various traditional medical systems, underscoring its high relevance in addressing region-specific public health challenges. This trend highlights the practical value and broad applicability of *S. rebaudiana* in addressing the increasing burden of chronic diseases.

In India, the prevalence of type 2 diabetes and hypertension ranks among the highest globally, and cardiovascular diseases (Raghavan et al., 2023) and oral health issues are also of significant concern (Dave, 2024). Consequently, within the Ayurvedic medical system, *S. rebaudiana* is widely employed for oral hygiene management (Pradeep et al., 2024; Balkrishna et al., 2025). Decoctions of its leaves are also used to regulate blood pressure, improve cardiovascular function and control body weight. Particularly, stevia is recommended for patients with diabetes as a natural, low-calorie sweetener to replace refined sugars, thereby reducing sugar intake and improving metabolic health (Das, 2013; Ali et al., 2024; Gorain et al., 2024; Manthattil Vysyan et al., 2024; Sharma and Alam, 2024). In addition, herbal pastes prepared from fresh or dried leaves are commonly used to promote wound healing and tissue repair (Manthattil Vysyan et al., 2024), demonstrating auxiliary efficacy in traditional caregiving practices.

In Thailand and other Southeast Asian countries, despite the gradual enhancement of modern healthcare systems, traditional healers continue to play a vital role in community-based primary health services (Maneenoon et al., 2015). As a commonly used local herb, *S. rebaudiana* has long been incorporated into traditional prescriptions for modulating metabolic functions and assisting in glycemic and weight control (Tipduangta et al., 2019; Wanyo et al., 2024). In recent years, its extracts have been widely used in functional skin care products. Studies have demonstrated their antioxidant, anti-inflammatory, skin-brightening, and anti-aging effects (Chaiyana et al., 2021). These applications align closely with regional trends in metabolic disorders (Reutrakul and Deerochanawong, 2016; Danpanichkul et al., 2024) and chronic skin inflammation (Prasitpuriprecha et al., 2022; Chaweekulrat et al., 2025) in Southeast Asia.

In TCM, *S. rebaudiana* is classified as sweet and neutral in nature, with traditional functions of clearing heat, generating fluids, moistening dryness, tonifying the stomach and liver, and lowering blood sugar and pressure (Wang, 2000; Ding et al., 2016; Bai et al., 2024; Waris et al., 2024). Clinically, it can be consumed alone as tea or as a decoction or used in combination with other herbs to treat hyperglycemia, hyperlipidemia, gastrointestinal discomfort, and cough, with particularly notable efficacy in managing hyperglycemia and hyperlipidemia, meeting the therapeutic needs of patients with comorbid metabolic syndrome (“three highs”) (Zhang et al., 2020). In pediatric applications, stevia is frequently used to mask the bitterness of traditional Chinese decoctions, thereby improving adherence to treatment and serving as a practical adjunct in the management of respiratory diseases (Ding et al., 2016).

In Korea and Japan, where lifestyle-related diseases and chronic inflammation are on the rise, increasing attention has been paid to the role of *S. rebaudiana* in lipid regulation, maintenance of intestinal barrier integrity, and suppression of pathogen-associated inflammation. Relevant studies have indicated that stevia may help prevent obesity-induced dysbiosis and metabolic inflammation (Aran et al., 2014; Kim et al., 2023; Han et al., 2024). Notably, its leaf extracts have already been widely incorporated into functional foods and cosmetic products in both countries (Goyal et al., 2010; Samuel et al., 2018).

In resource-limited countries such as Indonesia, Bangladesh, and Malaysia, *S. rebaudiana* is utilized as a culturally accepted and cost-effective natural intervention for dietary management in diabetic populations (Mayasari et al., 2018; Rahman et al., 2021). Its broad accessibility and favorable safety profile make it a valuable auxiliary tool in community-level strategies for the prevention of noncommunicable diseases (NCDs).

The diverse applications of *S. rebaudiana* across the traditional medical systems in Asia are grounded in their rich cultural heritage and reflect their wisdom in combating chronic metabolic and cardiovascular conditions. These traditional practices provide a solid foundation for mechanistic studies and translational medicine, underscoring the practical significance and developmental potential of Stevia in the field of public health.

#### 4.1.4 Applications in oceania

In Oceania, *S. rebaudiana* is primarily used as a natural sweetener and food additive, with its application in functional foods and health beverages has expanded significantly. Beyond its sweetening properties, *S. rebaudiana* is increasingly recognized as a complementary herbal therapy with the potential to regulate blood glucose levels and promote digestive health (Samuel et al., 2018). These applications are particularly important in regions where obesity, metabolic syndrome, and diet-related chronic diseases are increasing.

#### 4.1.5 Other applications

Although the integration of *S*. *rebaudiana* into traditional medical systems in Europe and Africa is less extensive than that in South America or Asia, its applications, particularly in blood glucose regulation, antioxidant activity, and digestive support, are similar to those in the Americas. In certain African regions, its incorporation into traditional therapies reflects a broader trend of integrating globally recognized medicinal plants into local healthcare systems under resource constraints. Overall, the widespread ethnopharmacological applications of *S. rebaudiana* across continents (see Table 2 for details) highlight its functional diversity and cross-cultural therapeutic value. These patterns underscore the increasing importance of the role of the environment in global public health efforts.

### 4.2 Development potential of other *stevia* species

In addition to *S. rebaudiana*, other species of the Stevia genus also possess various ethnomedicinal values, which are summarized in Table 3. Five other Stevia species with notable sweetness have been identified, although they are generally less intense than *S. rebaudiana*, including *Stevia lemmonii* var. hispidula, *Stevia micradenia*, *Stevia oligocephala*, *Stevia perfoliata*, and *Stevia phlebophylla* (Soejarto et al., 1982). These species also feature certain medicinal properties. The sweetness profiles of these species may be enhanced through the application of modern biotechnological tools, such as hybrid breeding and gene editing, to improve their development and commercial utilization, thereby reducing the overreliance on *S. rebaudiana.*

**TABLE 3:** Traditional uses of other *Stevia* species.

Species	Common name(s)	Region	Uses Part(s)	Preparation method	Traditional uses	Ref.
*S. bogotensis* Tr. ex Cortés	*Jarilla,* *Clavito,* *eupatoria*	Columbia	Leaves	--	Antipyretic; diaphoretic agent	Borgo et al. (2021)
*S. eupatoria* (Spreng.) wild	--	China	Leaves	--	Analgesic; anti-inflammatory; antihypertensive agent	Mlambo et al. (2022)
*S. cardiatica* Perkins	--	Bolivia	Leaves	--	Used to treat heart disease	Borgo et al. (2021)
*Stevia yalae* Cabrera	--	Argentina	Leaves	--	Ornamental plant	Borgo et al. (2021)
*Stevia achalensis*	Comadre	Argentina	Leaves	--	Ornamental plant	Borgo et al. (2021)
*S. collina* Gardn.	Caá-ehé	Brazil	Leaves	--	Sweetener, as stomachic	Borgo et al. (2021)
*Stevia connata* Lag.	Pericón de monte	Guatemala	Leaves	--	Used to treat stomachache	Borgo et al. (2021)
*S. elatior* HBK.	A-cí	Mexico	Leaves	--	Used to soothe burns and abrasions	Borgo et al. (2021)
*Stevia eupatoria* (Spreng.) Wild	Hierba del borrego, yerba del borrego, cola del borrego, estevia	Cuba	Leaves	--	Diuretic; antimalarial; used for gastric pain; hypoglycemic; analgesic, anti-inflammatory; antihypertensive agent	Borgo et al. (2021)
*Stevia glandulosa* Hook. et Arn.	Hierba de la pulga	Mexico	Roots and leaves	--	Antipyretic	Borgo et al. (2021)
*S. linoides* Sch. Bip.	--	--	Leaves	--	Astringent	Borgo et al. (2021)
*Stevia puberula* Hook.	Lima-lima	Peru	Leaves	--	Used as a tea substitute and stomachic	(Borgo et al., 2021; Chaiyana et al., 2021)
*Stevia rhombifolia* HKB var. stepphanocoma Sch. Bip.	Manka pak’I, pirq’a	Peru	Leaves	--	Used to treat stomachache; as an emetic; as an additive to yerba mate	Borgo et al. (2021)
*Stevia salicifolia* Cav.	Hierba del aire, hierba de la mula, la envidia, zazale de olor, yerba de la mula, Hierba de la Santa Rita	Mexico, United States	Leaves	--	Used for the treatment of rheumatism; as a laxative; for relieving intestinal discomfort caused by parasites; for the treatment of fever and colds	Borgo et al. (2021)
*Stevia linoides* Sch. Bip.	--	--	Leaves	--	Astringent	Borgo et al. (2021)
*Stevia lucida* Lag.	Yerba del aire, hierba de la araña, ma-li-too, kebuj, mariposa, chirca, chilca, javillo, golondrina de la sabanera	Mexico, Guatemala, Colombia, Venezuela	Leaves	--	Used for wound treatment; pain relief; rheumatism management; and as an anti-inflammatory agent	Borgo et al. (2021)
*S. macbridei* B. L. Robins var. *anomala* B. L. Robins	Jauja-huancayo	Peru	Leaves	Topical application	Used by women for bathing purposes	Borgo et al. (2021)
*S. petiolata *(Cass) Sch. Bip	Guarme-guarmi	Peru	Leaf extract preparation	Flavoring	To give flavor to meat	Borgo et al. (2021)
*S. puberula* Hook.	Lima-lima	Peru	Leaves	--	Used as tea substitute and stomach medicine	Borgo et al. (2021)
*S. pilosa *Lag.	Florde María	Mexico	Leaves	--	Antimalarial; antipyretic; laxative; diuretic	Borgo et al. (2021)
*Stevia nepetifolia* HBK	Zazal, anis de ratón, peracón	Mexico, Guatemala	Leaves	--	Used for the treatment of dysmenorrhea	Borgo et al. (2021)
*S. palmeri* Gray	Raniweri, raniwori	Mexico	Leaves	--	As an odoriferous herb; used to improve digestion; relieve anxiety; promote blood circulation; enhance flavor; repel insects, acting as a natural preservative; purifying the air	(Máthé, 2015; Borgo et al., 2021)
*Stevia plummerae* Gray	Ronino	Mexico	Leaves	--	To make washes and poultices for open wounds	Borgo et al. (2021)
*Stevia serrata* Cav.	Ronino, Uriki, Otoninawa, Chapo, yerba picante, hipericón, Q’ang’aj, anis silvestre, hipericon arrie	Guatemala, Mexico	Leaves	--	Used for cleansing and dressing open wounds; applied to foot cuts and snake bites; used as a remedy for coughs; for the treatment of gastrointestinal disorders	Borgo et al. (2021)
*Stevia subpubescens* Lag.	Hierba de la mula, Zazal	Mexico	Leaves	--	Used for postpartum bathing; treatment of stomachache; relief of joint pain	Borgo et al. (2021)
*Stevia trifida* Lag.	Manzanilla de agua	Mexico	Leaves	--	Used for the treatment of dysentery	Borgo et al. (2021)
*S. serrata*	--	Mexico	Roots	Decoction	Used to treat diabetes	Padilla-Mayne et al. (2024)
				Wash and poultice	Applied to open wounds for healing purposes	
				Flavoring	An ingredient used to sweeten the traditional fermented beverage “tesgüino”	
			Roots, leaves, and flowers	Soaked and consumed together with *tabardillo*	Used for the treatment of digestive disorders; such as indigestion or slow digestion; intestinal infections; gastric discomfort or pain; diarrhea	http://www.medicinatradicionalmexicana.unam.mx/apmtm/termino.php?l=3&t=stevia-serrata (accessed on 03.04 2025)
			Leaves and roots	Essential oil	Antinociceptive activity	Cordeiro et al. (2020)

## 5 Phytochemical metabolites and bioactivities of *S*. *rebaudiana*

**FIGURE 5:**
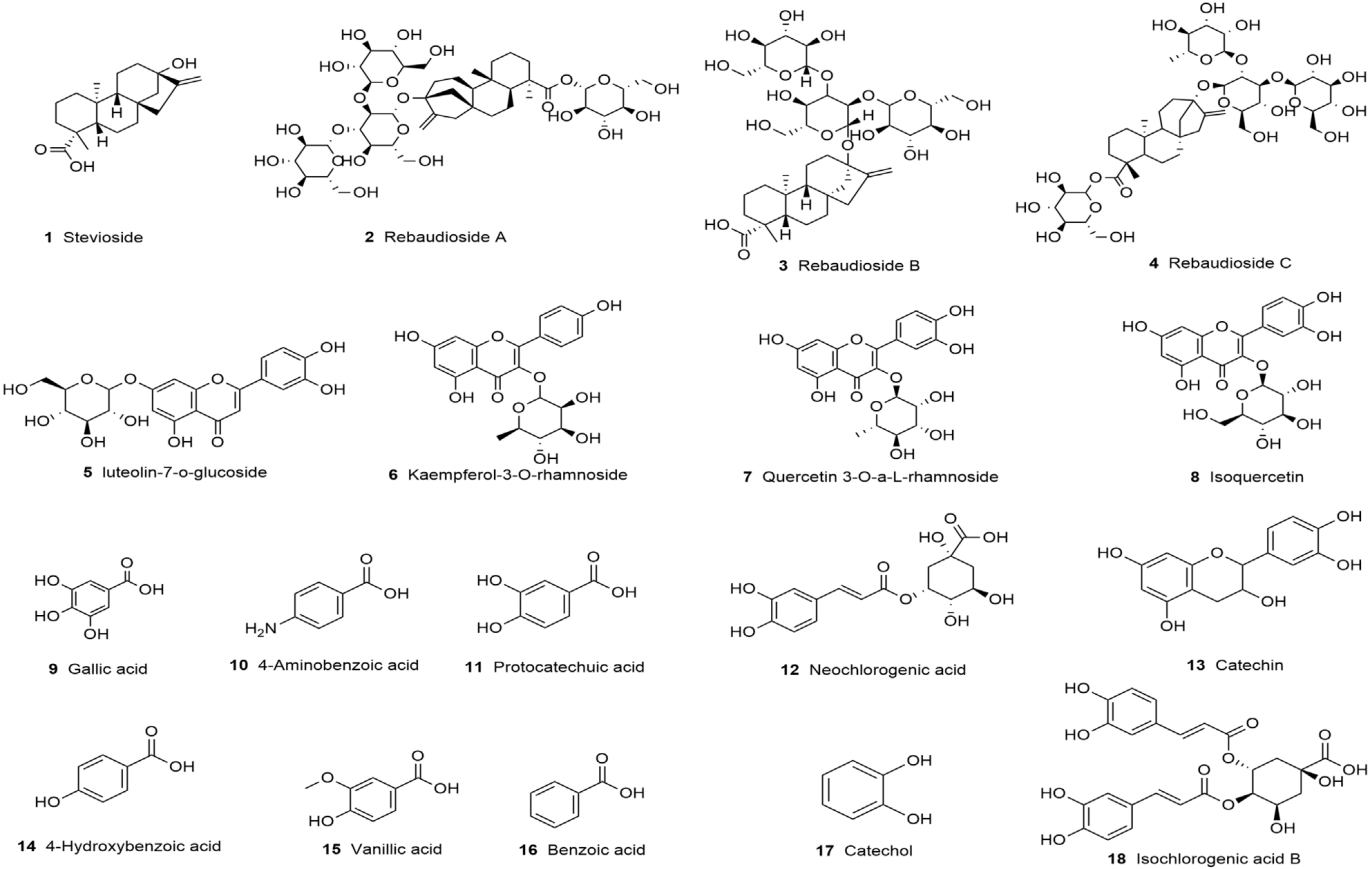
Chemical structure of diterpenoids, flavonoids and phenols in *S. rebaudiana* was prepared using ChemDraw.

In the herbal market, the primary medicinal part of *S. rebaudiana* is the leaves. To date, researchers have isolated and identified various bioactive constituents from its leaves, which can be broadly categorized into diterpenoids, flavonoids, polyphenols, phenylethanoid glycosides, amino acids, fatty acids, glycerolipids, and polysaccharides (Wölwer-Rieck, 2012; Molina-Calle et al., 2017; Prakash et al., 2017; Samuel et al., 2018; He et al., 2019; Myint et al., 2020; Kang et al., 2022). These phytochemicals are responsible for a wide range of pharmacological activities, including anti-inflammatory, antimicrobial, antioxidant, hepatoprotective, hypoglycemic, antihypertensive, cardioprotective, and antiparasitic effects (Koc et al., 2015). The following sections outline the major chemical constituents of *S. rebaudiana* and their associated biological activities, according to their chemical classifications. Figure 5 shows the representative chemical structures of key diterpenoids, flavonoids, and phenolic compounds isolated from *S. rebaudiana.*

### 5.1 Diterpenoids and derivatives

The leaves of *S. rebaudiana* are rich in diterpenoid glycosides, including stevioside, rebaudioside, steviolbioside, dihydroisosteviol, rubusoside, and dulcoside. Among these glycosides, stevioside and rebaudioside are the principal active constituents.

Rebaudioside has demonstrated antidiabetic activity by stimulating insulin secretion and exerting antihypertensive effects through the calcium channel-blocking mechanism (Ruiz-Ruiz et al., 2017). In addition, it can inhibit the release of pro-inflammatory factors, interleukin-6 (IL-6) and tumor necrosis factor alpha (TNF-α), through the nuclear factor kappa-light-chain-enhancer of activated B cells (NF-κB) signaling pathway to improve chronic inflammation (Ruiz-Ruiz et al., 2017).

Stevioside reduces blood glucose levels through multiple mechanisms, including activation of the Adenosine Monophosphate-Activated Protein Kinase (AMPK) and PI3K/Akt signaling pathways, regulation of hepatic glycogen metabolism, inhibition of α-glucosidase, and modulation of glucose transporter type 4(GLUT4) translocation (Ghanta et al., 2007; Lemus-Mondaca et al., 2012).

In addition, stevioside suppresses inflammation by downregulating the IκBα/NF-κB, MAPK, and TLR4-mediated immune signaling pathways, reducing the release of IL-6, TNF-α, interleukin-1 beta (IL-1β), and nitric oxide (NO) (Boonkaewwan et al., 2006; Boonkaewwan and Burodom, 2013), and regulating the Bax/Bcl-2/Caspase-3 signaling cascade to inhibit apoptosis, particularly in osteoarthritis and inflammation-related cartilage damage (Cai et al., 2023).

Stevioside enhances mitochondrial bioenergy metabolism by inducing PGC-1α expression, promoting fatty acid oxidation, reducing fat accumulation, and improving insulin sensitivity, thereby contributing to metabolic homeostasis (Park et al., 2022a). It also facilitates bile acid excretion and inhibits HMG-CoA reductase activity, thereby reducing cholesterol synthesis, lowering serum lipid levels, decreasing hepatic lipid deposition, and reducing cardiovascular risks (Melis, 1992).

Steviol glycosides, including stevioside and rebaudioside, have also shown significant antihypertensive effects through multiple mechanisms, including calcium channel antagonism, inhibition of angiotensin-converting enzyme (ACE) activity, promotion of natriuresis, and modulation of the sympathetic nervous system (Chan et al., 2000; Wang and Wu, 2019; Ray et al., 2020; Olas, 2022).

Other diterpenoid glycosides, such as steviolbioside, dihydroisosteviol, rubusoside, and dulcoside, have also demonstrated potential bioactivities in glycemic regulation, anti-inflammatory effects, and hepatoprotection (Ruiz-Ruiz et al., 2017).

Moreover, derivatives such as STVNa have been shown to attenuate high-fat diet–induced renal injury through antioxidant, anti-inflammatory, and anti-apoptotic mechanisms, offering therapeutic potential for obesity-related chronic kidney disease (Mei et al., 2020). Metabolites such as steviophethanoside and 6-O-acetyl-(12R)-epiblumdane have been reported to enhance insulin secretion and improve glucose metabolism (Bhasker et al., 2015; Prakash et al., 2017; Kang et al., 2022).

### 5.2 Phenolic metabolites and derivatives

Phenolic metabolites have attracted considerable attention because of their multiple biological functions, including free radical scavenging, metal chelation, and regulation of signaling pathways and enzymatic activities (Simoni et al., 2024). To date, more than 30 phenolic constituents have been identified in the leaves of *S*. *rebaudiana,* primarily including phenolic acids, flavonoids, and other polyphenol derivatives (Myint et al., 2020). These polyphenols are broadly involved in various physiological processes and exert pharmacological effects, such as antioxidant, anti-inflammatory, antiproliferative, and pro-apoptotic activities, through the modulation of signaling pathways, including PI3K/Akt, MAPK, CDK4/Cyclin D1, and p53. Their favorable bioactivities have been validated in multiple disease models, particularly demonstrating significant potential in chronic inflammation and metabolic disorders (Lopez et al., 2016; Myint et al., 2020; Zipinotti Dos Santos et al., 2023; Kaundal et al., 2024; Simoni et al., 2024; Zhu et al., 2024).

#### 5.2.1 Phenolic acids

*S. rebaudiana* is rich in various phenolic acids, including chlorogenic, caffeic, trans-ferulic, p-coumaric acid, and rhamnose acids, and their glycosidic derivatives (Celaya et al., 2022). Studies have shown that polyphenol extracts obtained from the leaves are dominated by chlorogenic acid and its isomers, indicating that phenolic acids are the major constituents of these extracts (Myint et al., 2020).

Due to high water solubility, these metabolites exhibit significant antioxidant and antimicrobial activities in aqueous extracts (Boling et al., 2020; Raghu and Velayudhannair, 2023). Their minimum inhibitory concentrations (MIC) ranged from 1.67 to 3.33 mg/mL for bacteria and from 6.67 to 13.3 mg/mL for fungi. In addition, they demonstrated inhibitory effects on several digestive enzymes, comparable to those of EGCG (Myint et al., 2023). Furthermore, phenolic acids can alleviate oxidative stress by activating the Nrf2/HO-1 signaling pathway and enhancing the activities of antioxidant enzymes such as SOD, CAT, and glutathione peroxidase (GSH-Px) (Myint et al., 2020). Their antimicrobial mechanisms primarily involve the disruption of cell membrane integrity, interference with DNA synthesis, and inhibition of biofilm formation (Myint et al., 2020). These metabolites also exhibit potent antiproliferative and pro-apoptotic effects against various tumor cell lines (Lopez et al., 2016; Myint et al., 2020; Zipinotti Dos Santos et al., 2023).

Processing methods significantly affect phenolic acid stability. Compared to fresh leaves, different drying treatments markedly increased the levels of caffeic acid and trans-ferulic acid (p < 0.05) (Lemus-Mondaca et al., 2018). Although steviol glycosides are the principal bioactive metabolites in *S. rebaudiana*, phenolic acids are also regarded as auxiliary health-promoting metabolites, potentially enhancing the overall functional value of *S. rebaudiana* as a nutraceutical (Wanyo et al., 2024).

#### 5.2.2 Flavonoids

Multiple flavonoid metabolites have been identified in the leaves of *S. rebaudiana*, primarily including flavonols (e.g., quercetin and kaempferol), flavones (e.g., rutin and luteolin), and their glycosidic derivatives (Wölwer-Rieck, 2012; Celaya et al., 2022). These metabolites are widely present in samples across different varieties with different extraction methods and form an important basis for their bioactivities. Notably, rutin is prone to degradation during the drying process (Lemus-Mondaca et al., 2021). Quantitative analyses indicate that the total flavonoid content in *S. rebaudiana* infusions and ethanolic extracts are 71.79 ± 0.00 and 56.66 ± 0.92 mg QE/g, respectively, suggesting that flavonoids are one of its major phenolic constituents (Andrade et al., 2021).

Flavonoids feature excellent antioxidant properties and capable of scavenging reactive oxygen species (ROS) and free radicals possibly by inhibiting ROS-generating enzymes, chelating metal ions, and donating hydrogen atoms. Their regulatory effects on metabolism, inflammation, and immune responses are primarily mediated through signaling pathways, such as sirtuin 1 (Sirt1)/AMPK and PI3K/Akt/mTOR, contributing to their potential in the intervention of diabetes and cardiovascular diseases (Yao et al., 2024). Their antiviral mechanisms include inhibition of viral entry, suppression of viral replication, and enhancement of host immune responses (Aghababaei and Hadidi, 2023).

In terms of functional specificity, quercetin mitigates fibrosis progression in diabetic nephropathy and chronic kidney disease by inhibiting the TGF-β/Smad signaling pathway (Aghababaei and Hadidi, 2023), whereas kaempferol regulates bile acid metabolism, promotes cholesterol efflux, and suppresses foam cell formation, thereby alleviating conditions such as NAFLD, NASH, and hypercholesterolemia (Yao et al., 2024). Luteolin demonstrates multi-target therapeutic potential in models of rheumatoid arthritis, hepatic fibrosis, and asthma (Zhu et al., 2024). Apigenin exhibits anxiolytic and antidepressant activities by modulating GABA receptor activity (Singh A. et al., 2024).

#### 5.2.3 Other polyphenols

In addition to phenolic acids and flavonoids, *S*. *rebaudiana* contains other types of polyphenolic metabolites, primarily including tannins and lignins. Tannins are among the common secondary metabolites in plants, and in *S. rebaudiana*, they are predominantly hydrolyzable tannins with a relatively high content (Andrade et al., 2021). These metabolites possess notable antioxidant and antimicrobial properties and may exert synergistic effects in the regulation of the gut microbiota and the delay of lipid oxidation. Furthermore, current research on lignins in *S. rebaudiana* remains limited, and systematic structural identification and functional validation are lacking; therefore, their associated biological activities require further investigation.

### 5.3 Amino acids and derivatives

The leaves of *S. rebaudiana* contain a total of 13 identified amino acids and their derivatives, including glutamate, proline, arginine, serine, lysine, etc. Among them, proline, choline, and serine are present at the highest concentrations and are believed to play essential roles in metabolic regulation and maintenance of cellular functions (Molina-Calle et al., 2017).

### 5.4 Fatty acids and derivatives

*S. rebaudiana* leaves are rich in fatty acids and their derivatives, including palmitamide, docosenamide (also known as erucamide), N-stearoyl valine, 16 types of glycerolipids (mono-, di-, and triglycerides), and four types of free fatty acids and their derivatives (Molina-Calle et al., 2017). These lipid compounds exhibit potential bioactivities, including lipid metabolism regulation, immune modulation, cardiovascular health maintenance, and oxidative stress balance.

For instance, linoleic acid improves lipid profiles by promoting lipid metabolism through Peroxisome Proliferator-Activated Receptor Gamma (PPARγ) activation, lowering LDL levels, and increasing HDL levels, thereby reducing the risk of cardiovascular diseases (Siddique et al., 2016). It can also inhibit pro-inflammatory signaling mediated by TLR4/NF-κB, reducing the expression of TNF-α, IL-6, and COX-2 to alleviate chronic inflammation and potentially improve insulin resistance via the IRS1/PI3K/Akt signaling pathway (Siddique et al., 2016).

6-Octadecenoic acid is believed to modulate macrophage polarization toward the M2 anti-inflammatory phenotype via PPARγ activation. Stearic acid, a key metabolite of cell membranes, contributes to maintaining membrane fluidity and enhancing nervous system stability, whereas palmitic acid is primarily involved in energy metabolism and adipose tissue formation (Siddique et al., 2016).

Additionally, *S. rebaudiana* leaves are rich in NADPH-dependent superoxide-generating lipoproteins (suprol), which may reduce inflammation and vascular injury by activating NADPH oxidase (Nox), inhibiting TLR4/NF-κB signaling, and modulating eNOS/NO-mediated vasodilation. These actions contribute to blood glucose regulation, immune balance, and atherosclerosis prevention (Isoyan et al., 2019).

### 5.5 Oligosaccharides

The leaves of *S. rebaudiana* contain various oligosaccharides, primarily γ-cyclodextrin, maltose and its phosphate ester derivatives, and trehalose. Among them, γ-cyclodextrin may function as soluble dietary fiber, promoting gut microbiota homeostasis. Additionally, it has demonstrated potential anti-obesity and lipid-lowering activities by regulating lipid metabolism (Molina-Calle et al., 2017).

### 5.6 Volatile oils

The volatile oils of *S. rebaudiana* leaves are rich in bioactive metabolites, including oxidized steviol, spathulenol, (E)-nerolidol, phytol, and α-cadinol, among others. These metabolites exhibit a wide range of biological activities, including insecticidal, antimicrobial, antioxidant, anti-inflammatory, respiratory protection, and neuroprotective effects (Benelli et al., 2020).

Furthermore, studies have shown that *S. rebaudiana* volatile oils can penetrate viral envelopes, interfere with viral adsorption, and inhibit the viral life cycle, demonstrating their potential therapeutic value against viral infections such as HSV-1/HSV-2, HIV, influenza (IFV), and SARS-CoV-2 (Siddique et al., 2016; Chen and Ye, 2022). The oils also disrupt bacterial cell membranes and affect biofilm formation, exhibiting broad-spectrum antimicrobial activity against *Staphylococcus aureus*, *Escherichia coli*, and *Salmonella* (Muanda et al., 2011). Additionally, *S. rebaudiana* volatile oils demonstrate neurotoxic and repellent properties against pests, such as aphids and whiteflies, by affecting their nervous systems and modulating oxidative stress responses (Benelli et al., 2020).

Moreover, ledene oxide-(II), a product of linalool oxidation, may have a neuroprotective effect, making it a potential candidate for treating neurodegenerative diseases. Geranyl vinyl ether may possess sedative and antispasmodic properties, suggesting its potential use in aromatherapy (Muanda et al., 2011).

### 5.7 Phenylethanol derivatives

The leaves of *S. rebaudiana* contain phenylethanol glycosides (PhGs), particularly the newly discovered steviophethanoside, which has shown potential for promoting insulin secretion, improving glucose metabolism, and potentially lowering blood glucose levels. The underlying mechanisms may involve the stimulation of pancreatic β-cells, regulation of glucagon levels, and modulation of glucose metabolism (He et al., 2019).

### 5.8 Sesquiterpenoids

The sesquiterpenoids in *S. rebaudiana* include various isomers, such as Sterebin I, J, E, F, M, and N. These metabolites have been detected in both polar and non-polar extracts and may exhibit anti-inflammatory and antimicrobial activities (Molina-Calle et al., 2017).

### 5.9 Other metabolites

Purines and their derivatives in *S. rebaudiana* may possess antioxidant and neuroprotective properties (Molina-Calle et al., 2017). Retinoid derivatives are potentially related to cellular differentiation, antioxidant activity, and vitamin A metabolism (Molina-Calle et al., 2017). Alkaloids have been shown to exhibit neuroprotective and anti-inflammatory effects, although their specific mechanisms require further investigation (Ruiz-Ruiz et al., 2017). Triterpenes and sterols may possess anti-inflammatory and cholesterol-regulating properties (Ruiz-Ruiz et al., 2017).

The diverse phytochemical metabolites found in *S. rebaudiana* leaves form the basis for its wide range of biological activities and associated health benefits. Table 4 summarizes the major metabolites identified in *S. rebaudiana* and their respective categories, facilitating the understanding of the composition of S. rebaudiana and providing a foundation for exploring its pharmacological mechanisms of action.

**TABLE 4:** Metabolite composition of S. rebaudiana.

NO.	Family		Metabolites	Ref.
1	Terpenoids	Diterpenoids	Austroinulin	Molina-Calle et al. (2017)
2			Steviol	
3			Steviolbioside	
4			Rubusoside	
5			Steviol + Glucose + 4-Methylglucuronide	
6			Dulcoside A	
7			Stevioside	
8			Steviol + 2 Glucoses4-Methylglucuronide	
9			Steviol + 2 Glucoses + 2Xyloses	
10			Rebaudioside A	
11			Rebaudioside B	
12			Rebaudioside C	
13			Rebaudioside D	
14			Rebaudioside E	
15			Rebaudioside G	Wölwer-Rieck (2012)
16			Dulcoside B	
17			Rebaudioside L	
18			Rebaudioside H	
19			Rebaudioside K	
20			Rebaudioside J	
21			Rebaudioside N	
22			Rebaudioside M	
23			Rebaudioside O	
24			Rebaudioside F	Molina-Calle et al. (2017)
25			Dulcoside A derivative	
26			Rebaudioside U	Liu et al. (2018)
27			Rebaudioside T	
28			Rebaudioside S	
29			Rebaudioside R	Liu et al. (2018)
30			Rebaudioside IX	Prakash et al. (2017)
31			Rebaudioside KA	Zhang et al. (2020)
32			6-O-acetyl-(12R)-epiblumdane	Kang et al. (2022)
33		Sesquiterpenoids	Sterebin I/J	Molina-Calle et al. (2017)
34			Sterebin E/F/M/N	
35	Phenolic metabolites	Flavonoids	Kaempferol monoglycoside	
36			Kaempferol rhamnoside	
37			Flavonoid monoglycoside	
38			Quercetin-3-O-arabinoside	
39			Quercitrin	
40			Flavonoid monoglycoside	
41			Flavonoid diglycoside	
42			Rutin	
43			Flavonoid coumaroylglucosideglucoside	
44			Quercetin 3-O (coumaroylglucoside)7-O-glucoside	
45			Flavonols	Wölwer-Rieck (2012)
46			Quercetin-3-O-β-D-rhamnoside	
47			Quercetin-3-O-glucoside	
48			Quercetin-3-O-rutinoside	
49			Quercetin-3-O-(4-O-trans-caffeoyl)-α-L-rhamno-pyranosyl-(1−6)-β-D-galactopyranoside	
50			Kaempferol-3-O-rhamnoside	
51			Apigenin	
52			Apigenin-4′-O-β-D-glycoside	
53			Apigenin-7-O-β-D-glycoside	
54			Luteolin-7-O-β-D-glycoside	
55		Quinic and caffeic acid and derivatives	Quinic acid	Molina-Calle et al. (2017)
56			Quinate phosphate	
57			1,5-Dicaffeoilquinic acid	
58			Caffeic acid	Myint et al. (2020)
59			4-Coumaric acid	
60			Cinnamic acid	
61			Syringic acid	
62			Vanillic acid	
63			4-Methoxybenzoic acid	
64			4-Methylcatechol	
65			Gallic acid	
66			Pyrogallol	
67			3,4,5-Tricaffeoylquinic acid	
68			1,3,5-Tricaffeoylquinic acid	
69			Rutin	
70			3-Feruloyl-5-caffeoylquinc acid	
71			4-Caffeoyl-5-feruloylquinic acid	
72			3,5-Dicaffeoylquinic acid (isochlorogenic acid A)	
73			1,4-Dicaffeoylquinic acid	
74			3,4-Dicaffeoylquinic acid (isochlorogenic acid B)	
75			1,3-Dicaffeoylquinic acid	
76			4,5-Dicaffeoylquinic acid (isochlorogenic acid C)	
77			Quercetin-3-O-glucoside	
78			Galuteolin	
79			Quercitrin	
80			Roseoside	
81			3-Feruloylquinic acid	
82			5-Feruloylquinic acid	
83			4-Caffeoylquinic acid (cryptochlorogenic acid)	
84			3-Caffeoylquinic acid (chlorogenic acid)	
85			5-Caffeoylquinic acid (neochlorogenic acid)	
86			5-p-Coumaroylquinic acid	
87			4-Caffeoylshikimic acid	
88			3-Caffeoylshikimic acid	
89			5-Caffeoylshikimic acid	
90			Catechin	
91			Luteolin	
92			Sinapic acid	
93			Trans-ferulic acid	
94			Caffeoyl-feruloylquinic acid	Wölwer-Rieck (2012)
95			feruloylquinic acid	
96			4-coumaric acid	
97			Pyrogallol	
98		Phenylethanoid Glycosides	4-hydroxyphenyl ethyl-8-O-[α- l-arabinopyranosyl-(1→6)]β-d-glucopyranoside (steviophethanoside)	He et al. (2019)
99			Pyrogallol	
100			Icariside D	
101			Salidroside	
102			Cuchiloside	
103			Tyrosol	
104	Other families	Amino acids and derivatives	Aminobutyric acid	Molina-Calle et al. (2017)
105			Serine	
106			Proline	
107			Pyroglutamic acid	
108			Choline	
109			Alanine	
110			Asparagine	
111			Glutamate	
112			Leucine/Isoleucine⁄	
113			Lysine	
114			Threonine	
115			Tryptophan	
116			Valine	
117		Fatty acid amides and derivatives	Palmitamide	
118			Oleamide	
119			Estearamide	
120			13-Docosenamide	
121			N-stearoyl valine	
122			Palmitamide	
123			Estearamide	
124			13-Docosenamide	
125			N-stearoyl valine	
126		Fatty acids and derivatives	Myristic acid	
127			Palmitic acid	
128			Stearic acid	
129			Oleic acid	
130			Oleic acid derivative	
131			Oleic acid derivative	
132			Gondoic acid derivative	
133		Glycerolipids	MG (16:1)	
134			MG (16:0)	
135			MG (18:1)	
136			MG (18:0)	
137			MG (20:1)	
138			MG (22:1)	
139			DG (18:1/0:0/20:1)	
140			DG (18:0/0:0/18:1)	
141			DG (18:1/0:0/16:0)	
142			DG (18:1/0:0/18:1)	
143			DG (18:1/0:0/20:0)	
144			DG (18:2/0:0/20:0)	
145			DG (20:0/0:0/20:1)	
146			DG (20:1/0:0/20:2)	
147			TG (16:1/20:0/20:0)	
148		Oligosaccharides	Gamma-cyclodextrin	
149			Disaccharide	
150			Trehalose	
151			Maltose + phosphate	
152		Purines	Purine	
153		Retinoids	Retinol derivative	

The bioactive metabolites of *S. rebaudiana* have demonstrated diverse pharmacological properties, including antioxidant, anti-inflammatory, and metabolic regulatory effects. These activities provide a strong basis for the application of these compounds in the prevention and management of chronic diseases, such as diabetes, obesity, and cardiovascular disorders, and support their incorporation into functional foods and nutraceutical products. Additionally, *S. rebaudiana* has shown significant antimicrobial properties, further expanding its therapeutic potential (Table 5). Moreover, to advance mechanistic studies and clinical translation, it is essential to prioritize representative metabolites based on their abundance and reported bioactivities, thereby identifying the most promising candidates for therapeutic development (Tables 6, 7).

**TABLE 5:** Antimicrobial categories of *S. rebaudiana*.

Category	Microorganism (Latin)	Extract type	Active results	Antimicrobial index (e.g., MIC/zone)	Mechanism (if known)	Ref.
bacteria	*Escherichia coli* O157	Fermented hot water extract	Exhibited bactericidal activity	Complete killing at 40% (v/v)	Acidic pH-dependent bactericidal effect; suppression of VT1/VT2 toxin production	Ruiz-Ruiz et al. (2017) Tomita et al. (1997)
	*Enterotoxigenic Escherichia coli* EC127	Fermented hot water extract	Viable count reduced from 10^9^ to <10^5^ cfu/mL within 2 h at 20% (v/v)	High susceptibility	Acidic pH disturbs extracellular environment and interferes with metabolic activity	
	*Enteroinvasive Escherichia coli* EC174	Fermented hot water extract	Viable count reduced from ∼10^9^ to <10^2^ cfu/mL within 2 h at 20% (v/v)	High susceptibility at 20%; total kill at 40% (v/v)	Acidic pH damages the cell membrane and leads to cell death	
	*Escherichia coli*	Crude methanolic extract	Exhibited bactericidal activity	Positive control: amoxicillin; negative: <1% DMSO; 69% inhibition rate	--	(Tomita et al., 1997; Sumit et al., 2008; Maryam et al., 2015; Ruiz-Ruiz et al., 2017; Chakma et al., 2023; Stachurska et al., 2023)
		Volatile extract (e.g., petroleum ether)	Exhibited bactericidal activity; complete inhibition at low concentration	MIC as low as 250 µg/mL	--	
		Aqueous extract	Inhibition observed	7 mm (low activity)	--	
		Ethanol extract (shaking or Soxhlet method)	Moderate to high antibacterial activity observed	Inhibition zone range: 6.53–18.81 mm; max: 18.81 mm	--	
	*Escherichia coli* DSM 5695	SRa (25%, acetone)	Initial inhibition followed by rebound	MIC: 25%; toxic	Concentration-dependent toxicity; weakened under dynamic conditions	Stachurska et al. (2023)
		SRm (25%, Methanol)	Enhanced lysis followed by rebound	MIC: 25%; detoxified but activated	Promotes lysis; growth stimulation under dynamic conditions	
	*Escherichia coli* DSM 613	SRa (25%, acetone)	OD significantly reduced	MIC: 25%; toxic	Toxic under static, but neutralized under dynamic conditions	
		SRm (25%, acetone)	No significant antibacterial activity	None	No apparent toxicity or inhibition	
	*Proteus mirabilis*	Polar extracts (methanol, ethyl acetate, hexane)	All extracts showed activity; hexane extract exhibited the strongest inhibition	Moderate to high activity	--	(Tomita et al., 1997; Sumit et al., 2008; Ruiz-Ruiz et al., 2017)
		Chloroform	Inhibition (10 mm zone of inhibition)	10 mm	Antibacterial activity	
	*Salmonella* spp.	Fermented hot water extract	Viability reduced from ∼10^9^ to <10^2^ cfu/mL within 2 h at 20% (v/v) concentration	High susceptibility	Low pH disrupts bacterial metabolism and inhibits growth	Tomita et al. (1997)
	*Staphylococcus aureus*	Crude methanolic extract	Inhibitory rate of 50%; Amoxicillin used as positive control; DMSO (<1%) as negative control	--	--	(Tomita et al., 1997; Sumit et al., 2008; Maryam et al., 2015; Ruiz-Ruiz et al., 2017; Chakma et al., 2023)
		Shaking extraction (ethanol: water = 80:20), 50 mg/mL	Highest inhibition zone reached 28.00 mm; range: 10.33–28.00 mm	10.33–28.00 mm; classified as high activity (>14 mm)	--	
		Fermented hot water extract (30%–40% v/v)	Significant inhibition after 2 h	Approx. 30%–40% (v/v); moderately sensitive	Acidic pH weakens cell wall synthesis, causing structural disruption	
	*Pseudomonas aeruginosa*	SRa (25%, acetone extract of *S. rebaudiana*)	No synergy with Phi6 phage; OD decreased under static condition	MIC: 25%; growth inhibition under static conditions	Weak toxicity; activity reduced under dynamic condition	(Sumit et al., 2008; Ruiz-Ruiz et al., 2017; Chakma et al., 2023; Stachurska et al., 2023)
		Shaking extraction (ethanol:water = 50:50), 25–50 mg/mL	Inhibition zone up to 14.67 mm; range: 6.27–14.67 mm	6.27–14.67 mm; moderate activity (10–14 mm)	--	
	*Pseudomonas syringae*	SRm (25%, Methanol)	No synergy with Phi6; flat growth curve	--	Limited impact on growth; no lysis or synergy	Stachurska et al. (2023)
	*Enterococcus faecalis*	Shaking extraction (ethanol:water = 50:50), 50 mg/mL	Inhibition zone up to 18.00 mm; range: 6.67–18.00 mm	6.67–18.00 mm; moderate to high activity (≥14 mm)	--	(Sumit et al., 2008; Chakma et al., 2023)
	*Bacillus subtilis*	Crude methanolic extract	Inhibitory rate of 51.8%; Amoxicillin used as positive control; DMSO (<1%) as negative control	Inhibitory rate: 51.8% (qualitative)	--	(Tomita et al., 1997; Sumit et al., 2008; Maryam et al., 2015)
		water extract	Inhibition zone approximately 8–9 mm	mild activity (8–9 mm)	--	
	*Vibrio parahaemolyticus*	Fermented hot water extract	<10% (v/v) extract significantly reduced colony count within 2 h; highly sensitive	Not numerically specified (highly sensitive)	Extremely sensitive to acidic pH; low concentration causes cell lysis	Tomita et al. (1997)
	*Bacillus cereus*	Fermented hot water extract	<10% (v/v) extract showed significant inhibition, but spores may survive	Not numerically specified; sensitive (spore resistance noted)	Acidic pH affects vegetative cells; spores highly resistant but partially inactivated	
	*Yersinia enterocolitica*	Fermented hot water extract	30%–40% (v/v) concentration caused significant inhibition within 2 h	Not numerically specified (moderately sensitive)	Acidic pH inhibits physiological activity and disrupts cell membrane integrity	
	*Listeria monocytogenes*	Stevia ethanolic extract	Inhibitory at 62.5–500 µg/mL; MIC = 125 µg/mL, MBC = 250 µg/mL; inhibited biofilm formation (P < 0.01), damaged membranes, caused nucleic acid leakage	MIC = 125 µg/mL; MBC = 250 µg/mL	Damages cell membrane, inhibits biofilm formation, induces nucleic acid leakage	Belda-Galbis et al. (2014)
Fungi	*Candida albicans*	Water, ethanol, and acetone extracts	Acetone extract showed strongest inhibition; more effective than water extract	Inhibition zone: 17–21 mm (acetone); lower for other solvents	Likely related to cell wall or membrane disruption	(Jayaraman et al., 2008; Ruiz-Ruiz et al., 2017)
	*Trichophyton rubrum*	Leaf extract	Maximum inhibition zone of 12 mm at a concentration of 7.5 µg/mL	Inhibition zone: 9–12 mm	Possibly disrupts cell membrane and/or cell wall integrity	(Jayaraman et al., 2008; Sumit et al., 2008)
	*A.niger*	Petroleum Ether	Inhibition (16 mm zone of inhibition	16 mm	Antifungal activity	Sumit et al. (2008) Sumit et al. (2008)
	*P. chrysogenum*	Cyclohexane	Inhibition (14 mm zone of inhibition)	14 mm	Antifungal activity	
	*A. solani*	Chloroform	Inhibition (16 mm zone of inhibition)	16 mm	Antifungal activity	
Viruses	*Rotavirus*	Methanolic leaf extract	Maximum inhibition ∼71.6% at 200 µg/mL; no significant cytotoxicity observed	IC_50_ ≈ 95 µg/mL; CC_50_ > 300 µg/mL	May interfere with viral adsorption/entry; activates host defenses	Takahashi et al. (2001)
	*Herpes Simplex* Virus Type 1	Polysaccharide fractions (SFW, SSFK)	Both fractions significantly inhibited HSV-1 infection. SFW: EC_50_ = 0.3 µg/mL, SI = 917; SSFK: EC_50_ = 18.8 µg/mL, SI > 53. Reduced plaque size post-entry.	SFW: EC_50_ = 0.3 µg/mL; SSFK: EC_50_ = 18.8 µg/mL; SI > 50	Interferes with viral adsorption, penetration, glycoprotein expression, and cell-to-cell transmission; heparin-mimetic competition	(Ceole et al., 2020; Aghababaei and Hadidi, 2023)
Parasites	*Trypanosoma cruzi*	DCM extract of *Stevia aristata*	100 µg/mL killed 100% of parasites *in vitro* within 96 h; EC_50_ = 47.9 µg/mL; significantly reduced cyst weight *in vivo* (5.47 → 2.55 g, p < 0.05)	EC_50_ = 47.9 µg/mL; total kill at 100 µg/mL (96 h)	--	Albani et al. (2022)
	*Entamoeba histolytica*	Stevioside (STV)	IC_50_ = 9.53 mM (24 h); induced cell membrane irregularity, autophagic vacuole formation, and morphological abnormalities; reduced liver damage *in vivo*	IC_50_ = 9.53 mM (24 h)	Disrupts membrane structure, induces autophagy-like vacuoles, inhibits cysteine protease expression and proteolytic activity	Ortega-Carballo et al. (2024)

*S. rebaudiana* acetone extract → SRa.

*S. rebaudiana* methanol extract → SRm.

Activity level based on inhibition zone diameter.

<7 mm: No activity.

7–10 mm: Low activity.

10–14 mm: Moderate activity.

14 mm: High activity.

Antibacterial Index (Abl): A comparative indicator of antimicrobial potency based on inhibition zone diameter (Abl ∝ zone size).

**TABLE 6:** Major metabolite Categories of *S*. *rebaudiana*, Their Bioactivities, Estimated Relative Abundance and Health Benefits.

Metabolites category	Representative compounds	Estimated relative abundance	Potential bioactivities and health benefits	Ref.
Diterpenes and Derivatives	Stevioside, Rebaudioside A Rebaudioside C Dulcoside A	Steviol glycosides 8%–20%; Stevioside, 4%–13%; Rebaudioside A, 2.3%–3.8%; Rebaudioside C, 1%–2%; Dulcoside A, 0.4%–0.7%; Rebaudioside D, ∼0.2%; Rebaudioside M, ∼0.1%	Antidiabetic, antihypertensive, anti-obesity	(Chatsudthipong and Muanprasat, 2009; Peteliuk et al., 2021; Zhou et al., 2021; Celaya et al., 2022)
Phenols and Derivatives	Gallic acid, Caffeic acid, Rosmarinic acid, Chlorogenic acid	Total phenolics (TPC) mg GAE/g DW:80.13–86.47; Total flavonoids (TFC) mg QE/g DW: 111.16–126.70	Antibacterial, anti-inflammatory, antioxidant	(Singh et al., 2024b; Molina-Calle et al., 2017; Liu et al., 2018; Kinki et al., 2021; Celaya et al., 2022; Chakma et al., 2023; Simlat et al., 2023; Kaundal et al., 2024)
		Total phenolics (TPC) mg GAE/g extract 259.96 ± 23.66 Total flavonoids (TFC) mg QE/g extract 247.41 ± 19.92		
		Total phenolics (TPC) mg GAE/g DW:5.3 Total flavonoids (TFC) mg CE/g DW:28.6 Condensed tannins (CTC) mg CE/g DW: 4.6		
Phenylethanoids	Tyrosol-like metabolites	--	Antimicrobial, antiviral, antioxidant, gut health promotion	He et al. (2019)
Polysaccharides	Arabinogalactan II Fructooligosaccharides	8.1g/100 g	Immunomodulatory, antioxidant, gut health promotion	(Kim et al., 2011; Molina-Calle et al., 2017; Li et al., 2021; Peteliuk et al., 2021)
Volatile Oils	Caryophyllene, Linalool, Geraniol, α-Humulene, Borneol	0.25% (v/w) in dry leaf	Antimicrobial, antiviral, anti-inflammatory	(Singh et al., 2024b; Bao et al., 2024)
Amino Acids and Derivatives	Leucine, Glutamine	11.2–16.0 g/100 g	Immune function enhancement, protein synthesis promotion	(Molina-Calle et al., 2017; Peteliuk et al., 2021)
Fatty Acids and Derivatives	Linolenic acid, Palmitic acid	1.9–3.73 g/100 g	Cardiovascular disease prevention, cholesterol reduction, metabolic improvement	(Molina-Calle et al., 2017; Peteliuk et al., 2021)
Oligosaccharides	γ-Cyclodextrin, Maltose phosphate derivatives, Trehalose	--	Gut health promotion, antidiabetic effects	Molina-Calle et al. (2017)
Sesquiterpenes	β-Caryophyllene, Farnesol, Humulene	--	Anti-inflammatory, antimicrobial	(Simlat et al., 2023; Bao et al., 2024)
Other	Nutritional and non-essential bioactive metabolites	Dietary fiber: 6.8–15.2 g/100 g Vitamin C: 14.98 mg/100 g Vitamin B_2_ (Riboflavin): 0.43 mg/100 g Folic acid: 52.18 µg/100 g Pyrogallol-type compounds (phenolic derivatives): 951.27 mg/100 g	--	(Kim et al., 2011; Kinki et al., 2021; Peteliuk et al., 2021)

GAE, gallic acid equivalent; QE, quercetin equivalent; DW, dry weight.

This table summarizes the major classes of chemical constituents identified in *S. rebaudiana* as reported in the current literature, including estimates of their relative abundance, representative monomers, and typical pharmacological activities of these monomers. It provides a reference for future targeted screening and functional investigations.

**TABLE 7:** Nutritional and Mineral Composition of *S*. *rebaudiana* Leaves (per 100 g dry weight).

Category	Component	Content (unit)	Ref.
Basic Nutritional Components (per 100 g dry leaf)	Moisture	5.5 g	Kinki et al. (2021)
	Ash	8.2 g	
	Crude protein	16.2 g	
	Crude fat	3.8 g	
	Crude fiber	7.9 g	
	Carbohydrates	58.8 g	
Mineral Content (per 100 g dry leaf)	Calcium (Ca)	359.6 mg/100 g	
	Sodium (Na)	102.9 mg/100 g	
	Potassium (K)	347.4 mg/100 g	
	Magnesium (Mg)	324.1 mg/100	
	Iron (Fe)	297.9 mg/100 g	
	Copper (Cu)	3.7 mg/100 g	
	Manganese (Mn)	9.4 mg/100 g	

## 6 Quality control of *S*. *rebaudiana*

With the growing global application of *S*. *rebaudiana* as both a natural sweetener and potential herbal medicine, quality control has become a focal point for regulatory bodies and the scientific community. Numerous countries have established regulatory frameworks centered on quantifying active constituents, impurity limits, and batch-to-batch consistency of herbal medicines. For instance, the European Food Safety Authority (EFSA) and the Joint FAO/WHO Expert Committee on Food Additives (JECFA) have provided specific guidelines on the purity, safety thresholds, and allowable impurities of steviol glycosides (JECFA, 2008; Peters and Burdock, 2008; EFSA Panel on Food Additives and Nutrient Sources added to Food, 2010; JECFA, 2010; Younes et al., 2020).

The pharmacologically active components of *S. rebaudiana*, such as steviol glycosides, flavonoids, and phenolic acids, are highly influenced by environmental and technical variables, including geographical origin, cultivar, growth period, harvest season, fertilization, and postharvest processing (Karaköse et al., 2015; Bao et al., 2024). These variables directly affect the pharmacological activity, clinical efficacy, and safety of the plant. Thus, establishing a standardized and quantifiable quality control system is crucial for the regulated development of this product.

In contrast to international standards, which mainly emphasize high-purity steviol glycosides, quality control efforts in China focus more on the botanical diversity of raw materials, the context of multi-herb formulations, and the reproducibility of the preparation methods. Given the extensive use of whole-leaf materials and polyherbal combinations in traditional Chinese medicine, an adaptive and multifaceted evaluation system is particularly important. This section highlights recent representative studies from China and compares them with international standards to provide insights into the global integration of plants.

In China, Although *S. rebaudiana* has not yet been included in the *Chinese Pharmacopoeia, 2020 edition*, a multidimensional quality assessment system has gradually emerged, encompassing four key areas: morphological identification, chemical fingerprinting, process standardization, and quantitative analysis of bioactive metabolites.

**FIGURE 6:**
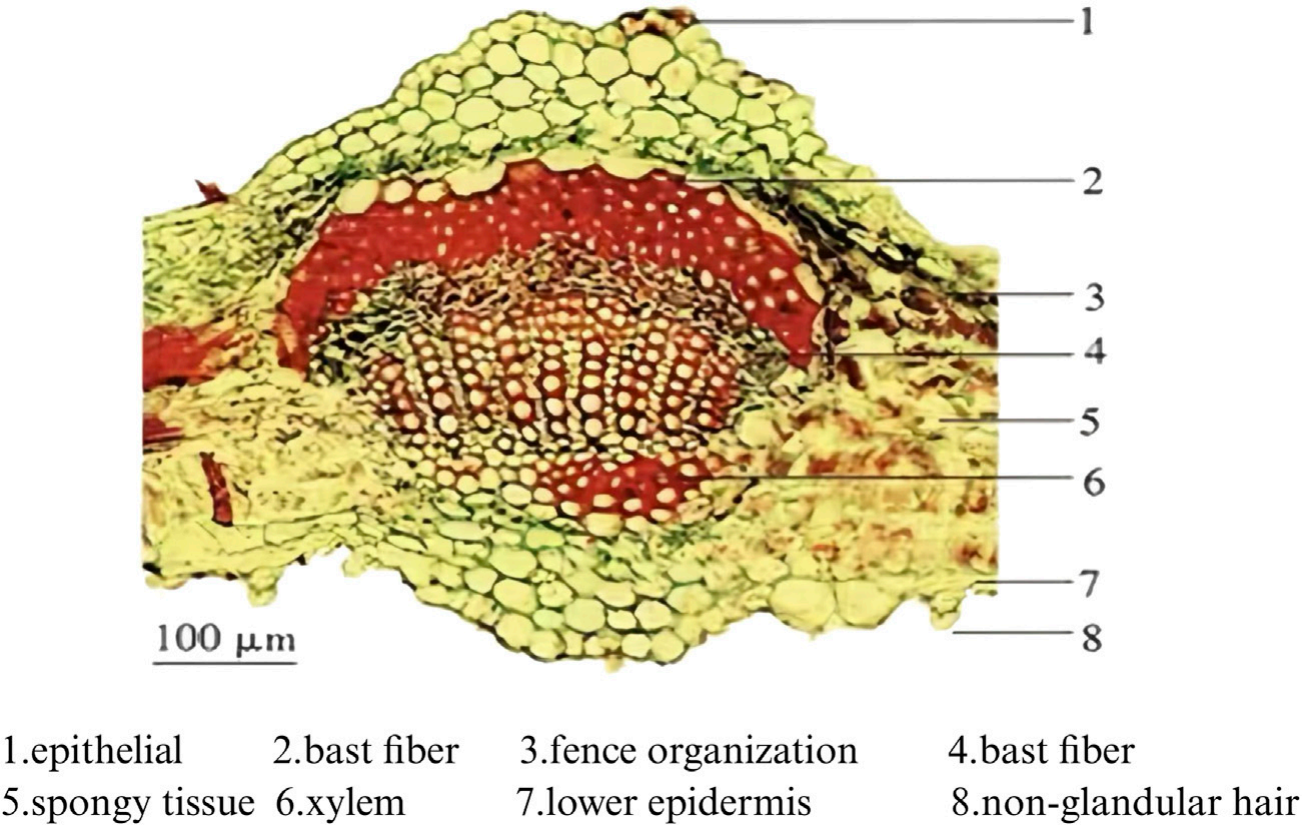
Detailed cross-section of *S*. *rebaudiana* leaf veins (https://yjj.gansu.gov.cn/yjj/c114435/202007/1301484.shtml).

**FIGURE 7:**
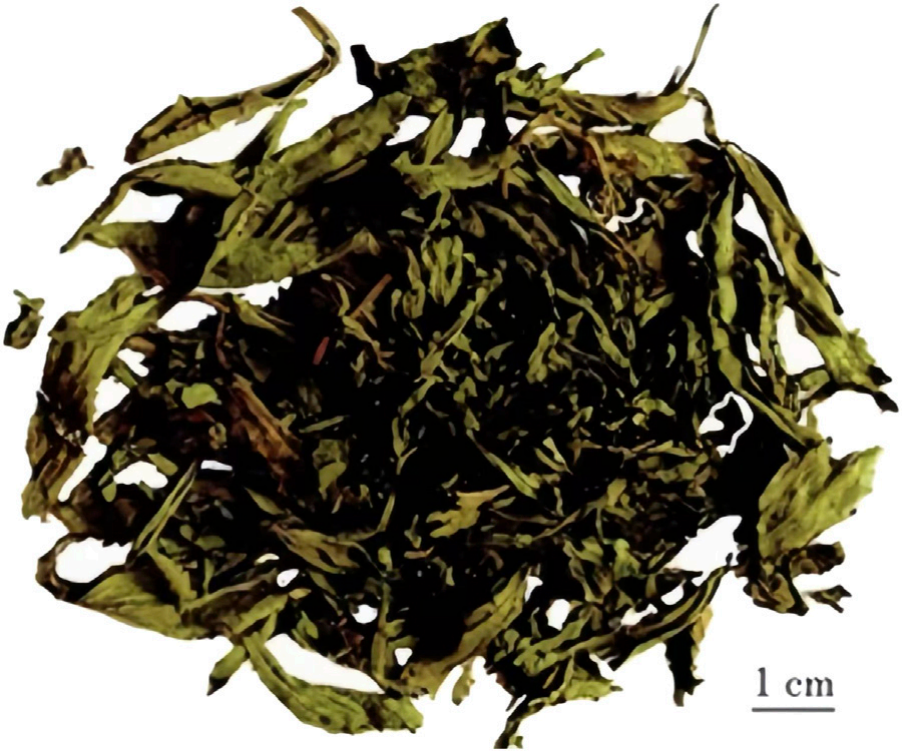
Dried leaves of *S*. *rebaudiana* after postharvest processing. The leaves retained their characteristic elongated and lanceolate shape with curled margins. These dried materials are commonly used as natural sweeteners because of their high steviol glycoside content. Scale bar = 1 cm (https://yjj.gansu.gov.cn/yjj/c114435/202007/1301484.shtml).

In terms of morphological identification, a local Chinese botanical medicine standard (*Gansu Provincial Standard of Chinese Medicinal Materials, 2020 editio*n) defines the appearance and microscopic characteristics of the dried leaves (Figures 6, 7). Drawing on the strategy proposed by Chen et al. in their study on Saussurea involucrata, a combination of polarized light microscopy, scanning electron microscopy (SEM), and macroscopic characterization can facilitate the differentiation of adulterant species within the same genus, enhancing the traceability of botanical sources (Chen et al., 2014).

For process standardization, Wu et al. (Wu et al., 2025) proposed a quality control strategy centered on the concept of a “standard decoction,” identifying stevioside content, total phenolic acids, and extract yield as critical quality indicators. A combined analytic hierarchy process and entropy weighting model was employed to derive a comprehensive score (mean value: 78.03), thereby constructing a “design space” for extraction processes to ensure batch-to-batch consistency and support the standardized production of Chinese herbal granules.

With regard to pharmacopoeial parameters, Guo et al. (Guo et al., 2014) recommended the inclusion of physicochemical indices, such as impurities (≤4%), moisture (≤11%), total ash (≤9%), and ethanol-soluble extractives (≥41%), alongside minimum content thresholds for bioactive metabolites (stevioside ≥2%, rebaudioside A ≥3%) in quality specifications to enhance extract consistency and pharmacological reliability.

In chemical fingerprinting and quantitative analyses, Li et al. (Li Z. et al., 2023) established a high-performance liquid chromatography (HPLC) fingerprint covering 21 batches of samples, with six stable peaks (including chlorogenic acid and its isomers) identified and validated for repeatability, making it suitable for raw material consistency evaluation. Further, Gao et al. (Gao et al., 2021) applied a strategy combining “reference fingerprinting + comprehensive scoring model” to achieve an integrated, precise, and cost-effective assessment of *S. rebaudiana* decoction pieces, confirming the feasibility and industrial applicability of this approach.

In summary, the quality control system for *S. rebaudiana* is evolving toward greater standardization, traceability, and multidimensional integration. This development provides a robust foundation for high-quality applications in Chinese herbal granules, functional foods, and pharmacological research.

## 7 The potential value of *S*. *rebaudiana* in population health and clinical applications

**FIGURE 8:**
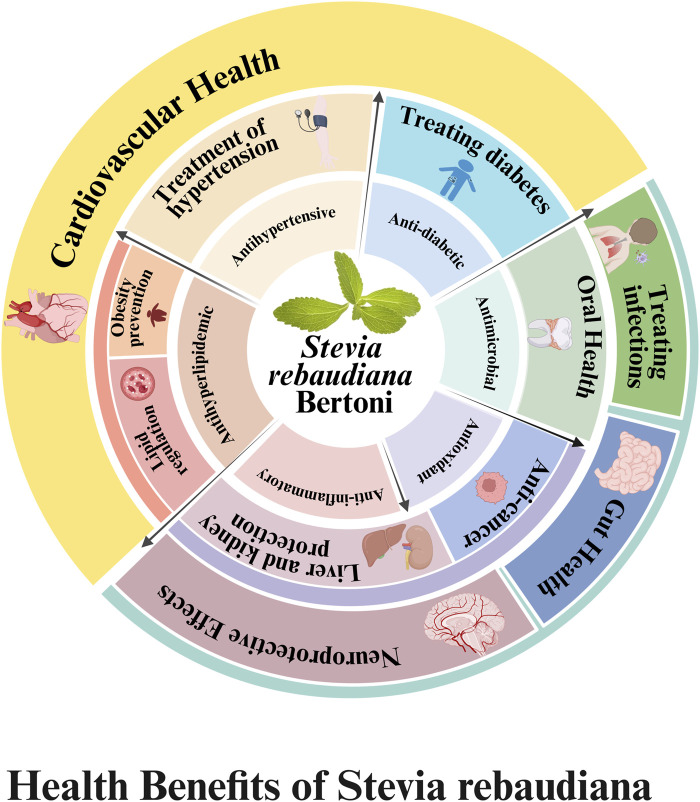
Bioactivities and health impacts of *S. rebaudiana* were created using BioRender.

Building on the identification of its bioactive components, it is particularly important to further explore the potential of *S*. *rebaudiana* for disease prevention and treatment. In recent years, as pharmacological studies have advanced, the functional applications of Stevia in both pediatric and adult populations have shown promising prospects. Table 8 summarizes representative *in vivo* and clinical studies on *S. rebaudiana*, illustrating its core pharmacological effects and therapeutic applications. The overall bioactivities and health effects of *S. rebaudiana* are summarized in Figure 8.

**TABLE 8:** Reported pharmacological activities and therapeutic potentials of *S*. *rebaudiana*.

metabolite(s)	Extraction type	Dose range	Experimental setting	Experimental subject	Positive/Negative control	Main pharmacological effect	Main pharmacological category	Ref.
Stevioside	Monomeric compound (infusion/oral)	Infusion 0.3 mg/mL; Oral 25 mg/kg/day	*In vitro* + *In vivo*	Isolated rat hearts and *in vivo* rat model	Untreated ischemia/reperfusion (I/R)	Cardioprotection via regulation of myocardial calcium homeostasis and energy metabolism; reduction of mitochondrial calcium overload; increased sarcoplasmic reticulum Ca^2+^ storage	Cardiovascular protection	Ragone et al. (2017)
Stevioside (Nan Kai Chemical Factory, Tien Jing, China)	Capsules (250 mg)	250 mg, 3 times/day for 4 weeks	Clinical	Human subjects (106 Chinese patients with mild hypertension)	Placebo-controlled	Significant reduction in systolic and diastolic blood pressure; no significant changes in body weight or biochemical parameters	Antihypertensive	Chan et al. (2000)
Stevioside	Pure compound (oral equivalent, modeled)	4 mg/kg bw (simulated yearly intake)	*In vitro* (gut model)	Human child gut microbiota (fecal samples from 3 healthy donors)	--	Increased microbial diversity (Actinobacteria, Enterobacteriaceae ↑); initial decline then stabilization of *Lactobacillus*; SCFA shift (butyrate and propionate ↑, lactate ↓); *E. coli* ↑; no significant antioxidant change	Gut microbiota modulation/Prebiotic	Gatea et al. (2021)
Stevioside (ST), Sucralose (SU), ST+SU	Pure compounds (not extract)	>3 weeks (dose not specified)	*In vivo* (oral)	Normal and diabetic rats (STZ-induced)	Insulin used as positive control	ST and ST+SU significantly reduced blood glucose in diabetic rats; SU alone ineffective. ST mitigated pancreatic damage and improved lipid profile. ST+insulin improved bilirubin and cholesterol. SU caused pancreatic lesions.	Antidiabetic	Barakat et al. (2023)
*S. rebaudiana* metabolites	--	--	*In vivo*	Diabetic rats (n = 40)	Metformin (positive control)	Significant reduction in fasting blood glucose (FBG); Increased serum insulin and reduced DPP-4 levels. Lower total cholesterol (TC), triglycerides (TGs), LDL, and increased HDL. Improved antioxidant markers (Glutathione (GSH) ↑, Malondialdehyde (MDA)↓). Enhanced metformin’s antidiabetic effect.	Antidiabetic/Cardiovascular Protection	Abdel-Aal et al. (2019)
Stevioside	sweetener	--	Open-label, single-arm study	Overweight and prediabetic adults (n = 45)	--	Significant weight loss (1.63 kg in overweight group, 1.34 kg in prediabetes group); waist circumference reduction (3.79 cm and 1.91 cm respectively). No significant effect on blood glucose, HbA1c, or lipid levels. No adverse effects reported.	Weight management, Metabolic health	Barakat et al. (2023)
*S. rebaudiana* metabolites	--	250 mg, twice daily	Randomized, single-blind, placebo-controlled clinical trial	Chronic kidney disease (CKD) patients (n = 97)	Placebo	Significant improvement in serum creatinine, uric acid, fasting and postprandial blood glucose, and microalbumin levels, supporting potential renal and metabolic health benefits.	Renal health, Metabolic health	Rizwan et al. (2018)
*S. rebaudiana* metabolites	Residue extract from Stevia leaves	--	Not specified	*In vivo*	Hyperuricemic mice	--	STR significantly reduced serum uric acid, BUN, and creatinine, restored inflammatory cytokine levels, reduced renal inflammation, fibrosis, and EMT. Regulated key pathways (NF-κB/NLRP3, AMPK/SIRT1, JAK2-STAT3, Nrf2). Strong anti-inflammatory and renal protective effects in hyperuricemic mice, suggesting therapeutic potential.	Zou et al. (2020)
Stevioside, Aspartame, Xylitol	Pure compounds	0.2% Sweetener in McBain Saliva	*In vitro* (microbial biofilm model)	228 bovine enamel and dentin samples exposed to human saliva	Sucrose (positive control), xylitol (negative control)	Stevioside, aspartame, and xylitol significantly reduced lactate production (92% reduction compared to sucrose). All sweeteners (Stevia, Aspartame, Xylitol) had similar effects on lactate production. Stevia, aspartame, and sucrose showed no significant difference in enamel and dentin lactate production. Decreased demineralization (85% and 83%) with Stevia and Aspartame showing no significant difference compared to sucrose.	Caries Prevention	Augustinho do Nascimento et al. (2021)
Stevioside	--	1 g per test meal	Acute paired crossover human trial	12 type 2 diabetic patients (4 female/8 male; mean age 65.8 years)	1 g cornstarch (control)	Stevioside reduced postprandial glucose iAUC by 18% (P = 0.013); increased insulinogenic index by ∼40% (P < 0 0.001); insulin AUCI showed increasing trend (P = 0.08); no significant changes in GLP-1 or GIP; slight reduction in glucagon; no adverse effects	Insulinotropic effect	Gregersen et al. (2004)
Stevioside	Purified stevioside capsule	500 mg, 3 times daily, for 2 years	Randomized, double-blind, placebo-controlled human trial	174 Chinese adults (87 female, 87 male) with mild primary hypertension (aged 20–75)	Placebo capsule, 3 times daily	Significant reductions in blood pressure: SBP from 150 to 140 mmHg, DBP from 95 to 89 mmHg (P < 0.05); improved quality of life (P < 0.001); lower incidence of left ventricular hypertrophy (LVH: 11.5% vs. 34%); no adverse effects reported	Antihypertensive	Hsieh et al. (2003)
Crude steviosides	Crude extract capsule	3.75, 7.5, 15.0 mg/kg/day (7, 11, 6 weeks respectively), b.i.d.	Randomized, double-blind, placebo-controlled trial	Untreated patients with mild hypertension	Placebo (4 weeks) followed by dose-escalation	SBP and DBP decreased in treatment group (p < 0.05), but similar changes in placebo group; no statistically significant difference observed; no adverse effects reported	Antihypertensive	Ferri et al. (2006)
Stevioside, Rebaudioside A	Aqueous solution (10%)	10% (w/v); 1 min rinse	*In vitro* (S. mutans biofilm via MTT assay) and *in vivo* (human dental plaque pH)	20 healthy human volunteers	Sucrose solution (10%) as positive control	Main Pharmacological Effect Did not promote S. mutans biofilm formation *in vitro*; prevented plaque pH drop *in vivo* after rinsing, unlike sucrose	Anti-caries/Antimicrobial	Brambilla et al. (2013)
Stevia-based rinse (steviol glycosides)	Aqueous rinse solution (6.4%)	Single dose, 1-min rinse	Human clinical study (parallel-group, single-blind)	Healthy adult volunteers	Tagatose, Sucrose	Significant differences in CFU/mL at 30 min and salivary pH at 48h; antimicrobial potential observed	Oral health/Antimicrobial	Urrutia-Espinosa et al. (2024)
Reb-A	Purified metabolite	10–30 μM	*In vitro* (cellular mechanism)	Human hepatoma HepG2 cells	Pre-treatment with Reb-A (1 h) followed by CCl4 (0.4% v/v) for 24 h	Reb-A reduced cell death and oxidative stress via PKCε-JNK/ERK-Nrf2 signaling pathway; upregulated antioxidant enzymes HO-1, NQO1	Hepatoprotective, Antioxidant	Wang et al. (2018)
*S. rebaudiana* aqueous extract	Water extract	100 mg/kg/day, oral for 15 days	*In vivo*	Fixed stress-induced hyperglycemic rabbit model	Compared to hyperglycemic control group	Significant reductions in blood glucose, TC, TG, LDL, AI; increased HDL, liver and muscle glycogen; attenuated weight loss	Antihyperglycemic, antihyperlipidemic, liver/muscle protective	Aghajanyan et al. (2017)
Stevioside	--	10 mg/kg, oral, 7 days prior to injury, continued for 3 or 7 days	*In vivo*	Adult male Wistar rats (TA muscle cardiotoxin injection model)	Contralateral limb (left leg) as internal control	No significant increase in myofibrillar protein; significant increase in MyoD + nuclei (P < 0.05), reduction in NF-κB nuclear translocation (P < 0.05)	Muscle regeneration/Anti-inflammatory	Bunprajun et al. (2012)
*S. rebaudiana* extract	--	400 mg/kg orally for 30 days	*In vivo* diabetes model	Male albino rats with STZ-induced	--	Significantly reduced fasting blood glucose (from 264.2 ± 8.9 to 137.3 ± 7.3 mg/dL), improved islet morphology, increased insulin levels, and showed no toxicity	Antidiabetic	El-Hadary and Sitohy (2021)
Steviol glycosides	--	200 mg/kg/d, 500 mg/kg/d, oral, 28 days	*In vivo*	STZ-induced diabetic rats	--	200 mg/kg/d: Mild reduction in TC, TG, LDL, slight increase in HDL, upregulation of CPT1 and PPARα, inhibition of Cebpa expression. 500 mg/kg/d: Significant reduction in TC, TG, LDL, significant increase in HDL (P < 0.05), upregulation of PPARα, CPT1, HSL, inhibition of sterol regulatory element-binding protein 1c (SREBP-1c), ACC, FAS, Cebpa, Fasn expression.	Lipid metabolism regulation/Antidiabetic	Kurek et al. (2023)
Stevia whole leaf powder, Polyphenols/Fiber extract	--	4.0% Stevia leaf powder in diet, 1 month	*In vivo*	Wistar rats (n = 80), STZ-induced diabetes (60 mg/kg i.p.)	--	Significant reduction in blood glucose, ALT, AST; increased insulin levels; improved liver MDA, antioxidant enzyme activity; enhanced glucose tolerance and insulin sensitivity; alleviated GFR decline and renal damage. Fiber group showed weaker effects.	Antidiabetic/Antioxidant/Hepatoprotective/Renoprotective	Shivanna et al. (2013)
Stevioside	--	100 and 200 mg/kg/day, oral, 8 weeks	*In vivo*	Male C57BL/6J mice, high-fat diet induced obesity, metabolic dysregulation, and anxiety/cognitive impairment	--	200 mg/kg: Significant reduction in body weight, blood lipids (TC, TG, LDL-C), fasting blood glucose (FBG); improvement in HOMA-IR, insulin levels, and sensitivity; reduction in liver fat accumulation; regulation of lipid metabolism genes (Glut4 ↑, Fasn ↓, Cebpa ↓); improvement in anxiety (elevated plus maze) and cognitive impairments (water maze); increased hippocampal BDNF and GABA_A expression; decreased inflammation markers (TNF-α, IL-1β).	Antidiabetic/Anti-inflammatory/Cognitive and Anxiety regulation	Khakpai et al. (2023)
Steviol	--	0.5, 1.0, 3.0 mg/kg/h, intravenous	*In vivo*	Male Wistar rats (n = 30)	30 min control period + 30 min experimental period	Effect on renal clearance rates (Glucose C_9_, PAH C_p_, Inulin C_i_), sodium/potassium fractional clearance rates (FeNa^+^, FeK^+^), urine flow rate (V/GFR)	Renal function regulation	Melis et al. (2009)
Steviol, Stevioside	--	10^−8^ to 10^−5^ M	*In vitro*	L6 muscle cells (diabetic induced), 3T3-L1 adipocytes	Insulin-treated groups	Significant increase in glucose uptake (2-NBDG), upregulation of GLUT4 expression and membrane translocation, enhanced Akt phosphorylation in the absence of insulin (P < 0.001)	Insulin-mimetic/Antidiabetic	Bhasker et al. (2015)
Stevioside, Rebaudioside	--	Stevioside 30 mg/kg, Rebaudioside A 100 mg/kg, i.p.	*In vivo* + Computational prediction	Swiss mice, MES and PTZ-induced epilepsy models	--	Predicted binding to voltage-gated sodium channels (Nav1.2/1.6) with anti-epileptic activity. *In vivo*: Dose-dependent suppression of seizures, prolonged latency, reduced seizure rate in MES model (P < 0.05).	Antiepileptic/Neuroprotective	Di Ianni et al. (2015)
Stevia ethanol extract	Ethanol extract	200 mg/kg, oral, 4 weeks	*In vivo* (Type 2 diabetes)	db/db mice	--	Improved insulin sensitivity, upregulated PGC-1α, NRF1, TFAM, reduced ROS and MDA, enhanced mitochondrial function	Antidiabetic/Mitochondrial regulation	Han et al. (2023)
Stevia residue extract (STVRE)	Water extract	75, 150, 300 mg/kg/day	*In vivo*	Male Kunming mice (high UA induced by 10% fructose, potassium oxonate, yeast extract)	Positive: Allopurinol 5 mg/kg	Reduces serum UA, XOD activity, inflammation markers (COX-2, NF-κB, PGE2, TNF-α, IL-1β), increases antioxidant enzymes (SOD, CAT, GPx)	Antihyperuricemic/Renal protective	Mehmood et al. (2020)
*S. rebaudiana* leaf extract	Water extract (butanol fraction); isolated stevioside	Not precisely stated; 0–1,000 μg/mL	*In vitro*	Guinea pig ileum (smooth muscle)	ACh, Histamine, CaCl_2_ induced contraction	Butanol extract and stevioside significantly inhibited ACh-, Histamine-, and CaCl_2_-induced contractions, acting as Ca^2+^ channel blockers	Antispasmodic	Shiozaki et al. (2006)
Stevioside, Rebaudioside A	--	Human: Dietary intake; *Cebus apella*: Equivalent exposure	*In vivo*	Healthy human volunteers and *Cebus apella* (capuchin monkeys)	--	Altered gut microbiota composition and diversity; Increased SCFA (butyrate) levels; No pathogenic overgrowth	Gut microbiome modulation/Prebiotic-like effect	Mahalak et al. (2020)
*S. rebaudiana* leaf	Leaf extract (aqueous)	0%–4%	*In vitro*	--	*Streptococcus* mutans	Reduce acid production and biofilm formation by S. mutans	Antibacterial/Dental	Escobar et al. (2020)
*S. rebaudiana* leaf extract	Water extract	300 mg/kg, oral, 28 days	*In vivo*	STZ-induced diabetic Wistar male rats (60 mg/kg)	Normal control group and diabetic model group	Significant reduction in GRP78, CHOP, and caspase-12 expression; improved ALT/AST levels; alleviated ER stress and apoptosis; histological recovery in liver tissue	Antidiabetic/Hepatoprotective	Saadi et al. (2024)
Stevioside	Commercially sourced (purity ≥98%)	25, 50, 100 μM	*In vitro* (MTT assay; qPCR for apoptosis genes)	Human prostate cancer cell line (PC-3)	DMSO (vehicle control); no positive drug	Stevioside significantly reduced cancer cell viability (MTT assay) in a dose-dependent manner. Induced apoptosis via increased Caspase-3 expression and decreased Bcl-2 and Mcl-1 expression (anti-apoptotic genes). Dose-dependent apoptosis induction (p < 0.05).	Apoptosis Induction	Preethi et al. (2023)
Stevioside	--	25, 50, 100 μM	*In vitro*	Human osteosarcoma cell line (SaOs 2)	--	Stevioside significantly inhibited osteosarcoma cell proliferation in a dose-dependent manner (MTT assay). Induced apoptosis through upregulation of pro-apoptotic Bax and downregulation of anti-apoptotic Bcl-xL and Bcl-2 genes via mitochondrial pathway.	Apoptosis Induction	Prithiksha and Priyadharshini (2024)
Stevioside (ST)	--	--	*In vitro*	Human breast cancer cell line (MCF-7)	--	Stevioside induced apoptosis through ROS production, mitochondrial membrane potential (MTP) disruption, and activation of mitochondrial apoptotic pathway involving Bax, Bcl-2, and Caspase-9. It may also have potential effects on stress-related transcription factors, especially NF-E2 related factor-2.	Apoptosis Induction	Paul et al. (2012)
steviol	--	100–250 μg/mL	*In vitro* cytotoxicity assay and gene expression analysis	Human gastrointestinal cancer cells (6 lines incl. DLD-1, HCT-116)	5-FU (positive); untreated control	Inhibits proliferation via mitochondrial apoptosis pathway (↑Bax/Bcl-2 ratio, activation of p21, p53); caspase-3-independent mechanism involved	Anti-proliferation	Chen et al. (2018)
Glycosylated isosteviol derivatives (e.g., 9a, 9e, 13a)	Semisynthetic glycosides from isosteviol	1–100 μM; some >50% inhibition at 25–50 μM	MTT assay; some Annexin V apoptosis assay	HL-60, HCT-116, MCF-7	Cisplatin (positive); DMSO (vehicle control)	Inhibited proliferation; some induced apoptosis (e.g., 9a, 13a in HL-60); improved water solubility	Apoptosis induction	Sharipova et al. (2019)
Glycosides and glycoconjugates of isosteviol (e.g., compound 13b)	Semisynthetic derivatization of isosteviol via glycosylation and linker conjugation	IC_50_ = 2.56–25.6 μM in HL-60; tested at 1–100 μM	*In vitro* cytotoxicity (MTT) + apoptosis (Annexin V-FITC/PI) + mitochondrial depolarization + G0/G1 cell cycle arrest	Human leukemia HL-60 cells	DMSO (vehicle); no chemical positive control used	Dose-dependent cytotoxicity; induced apoptosis via mitochondrial pathway (indicated by mitochondrial membrane potential loss); G0/G1 arrest; some compounds more potent than isosteviol	Apoptosis induction, activate mitochondrial pathway	Li et al. (2023a)
α-Fe_2_O_3_ nanoparticles biosynthesized using Stevia rebaudiana leaf extract	Green synthesis (*in situ* phytochemical-assisted reduction of Fe^3+^ using aqueous Stevia extract)	6.25–200 μg/mL; IC_50_ ≈ 31.28 μg/mL in HepG2	*In vitro* cytotoxicity by MTT assay	HepG2 (human hepatocellular carcinoma cells)	5-Fluorouracil (positive); DMSO (vehicle)	Significant cytotoxicity; dose-dependent inhibition of HepG2 cell viability	Cytotoxicity	Alshawwa et al. (2022)
Isosteviol, Steviol, Stevioside, Rebaudioside A	Isolated from Stevia rebaudianaleaves by methanol extraction and subsequent purification	6.25–200 μg/mL; IC_50_ for isosteviol = 37.02 μg/mL (MCF-7	*In vitro* MTT cytotoxicity assay	Human breast cancer cell line MCF-7	Doxorubicin (positive); DMSO (vehicle)	Doxorubicin (positive); DMSO (vehicle)	Cytotoxicity	Khatun et al. (2021)
Isosteviol derivatives	Semisynthetic from steviol	1–100 μM; IC_50_: 3.4–67.8 μM	*In vitro* MTT and apoptosis assays (Annexin V-FITC/PI)	HL-60 (highly sensitive); HSC-2, HSC-3 (moderate); Caco-2, HSC-4 (resistant)	DMSO control	Induced apoptosis in HL-60 cells; selective cytotoxicity varies by cell line; no mitochondrial or caspase assays conducted	Apoptosis induction	Ukiya et al. (2013)
Isosteviol and derivatives (e.g., isosteviol-thiadiazole analogs)	Semi-synthetic derivatives from *S. rebaudiana*	1–100 μM; IC_50_ = 4.4–58.4 μM (for Pol α inhibition)	*In vitro* enzyme inhibition assays and cancer cell proliferation tests	Human cancer cell lines (HL-60, HCT-116, etc.)	Etoposide (Topo inhibitor), 5-FU (Pol inhibitor)	Inhibited human DNA polymerase α, λ, γ and topoisomerase I activity; induced apoptosis; selective inhibition of tumor cells	DNA replication inhibition, topoisomerase inhibition	Mizushina et al. (2005)
Stevioside	Isolated from *S. rebaudiana* leaves	5–100 μM; IC_50_: 55 μM (MDA-MB-231), 66 μM (SKBR3)	*In vitro* cytotoxicity and chemosensitization assay	Human breast cancer cell lines (MDA-MB-231, SKBR3)	5-FU alone; untreated control	Induces apoptosis, increases ROS, enhances chemosensitivity to 5-FU, activates caspase-3/9, alters Bax/Bcl-2 ratio, DNA fragmentation	Chemosensitization, apoptosis induction	Khare and Chandra (2019)
Steviol	Isolated from *S. rebaudiana* leaves (purified standard compound)	10–250 μM; IC_50_ = 185 μM in MCF-7	*In vitro*: SRB assay (cytotoxicity); PI staining (cell cycle); AO/EB dual staining (apoptosis); ROS assay	MCF-7 (human breast cancer cell line)	DMSO (vehicle); no chemical positive control used	Induced apoptosis; dose-dependent cytotoxicity; G2/M phase arrest; reduced ROS levels; increased sub-G0/G1 apoptotic population	Apoptosis induction via ROS-mediated pathway; cell cycle regulation	Gupta et al. (2017)
Steviolbioside	Enzymatic hydrolysis of stevioside using β-galactosidase	25–400 μg/mL; IC_50_(HepG2) ≈ 80 μg/mL; SGC-7901 ≈ 110 μg/mL	*In vitro* (MTT assay, colony formation assay	Human hepatoma (HepG2), gastric carcinoma (SGC-7901), colon cancer (Caco-2), normal hepatocytes (L02), and over 30 additional human-derived cancer and normal cell lines	DMSO (vehicle); 5-FU (positive control)	Dose-dependent inhibition of cancer cell viability; weak effect on normal cells; no mechanism elaborated (no ROS, apoptosis markers reported)	Cytotoxicity effect	Chen et al. (2016)

### 7.1 Pharmacological potential of *S. rebaudiana* in pediatric populations

#### 7.1.1 Prevention of dental caries

The World Health Organization (WHO) guidelines indicate a clear association between the consumption of free sugars and the incidence of dental caries in children. When the intake of free sugars exceeds 10% of the total energy consumption, the incidence of dental caries is significantly higher (WHO, 2015).

Many children opt for oral solutions, syrups, or suspensions when treating colds, as these formulations are easy to swallow and often contain sweeteners and flavoring agents to mask the bitter taste of medicines (Alessandrini et al., 2021). Similarly, when using traditional Chinese herbal decoctions to treat colds, children often require candies to mask bitterness or rewards to improve medication adherence. Without timely oral care, the risk of developing dental caries increases significantly.

*S. rebaudiana* leaves have been shown to inhibit cariogenic bacteria in the oral cavity. With a lower acid production capacity, S. rebaudiana effectively reduces plaque formation, thereby preventing dental caries (Ding et al., 2016; Ruiz-Ruiz et al., 2017). Adding *S. rebaudiana* to decoctions not only improves the taste, enhancing children’s medication adherence, but also helps avoid excessive candy consumption to mask bitterness or neglecting oral care, thus reducing the risk of childhood caries.

#### 7.1.2 Management of inflammatory conditions

Inflammation, an early host immune response mediated by immune cells and their secreted cytokines, plays a vital role in combating infection and injury (Kaundal et al., 2024). Excessive activation of inflammatory responses can cause significant tissue damage, particularly in children (Slykerman et al., 2023). Moreover, children are particularly susceptible to oxidative stress induced by free radicals during rapid growth and development. The intake of natural compounds with antioxidant and anti-inflammatory properties can help mitigate inflammatory responses and support healthy development (Demirci-Cekic et al., 2022).

**FIGURE 9:**
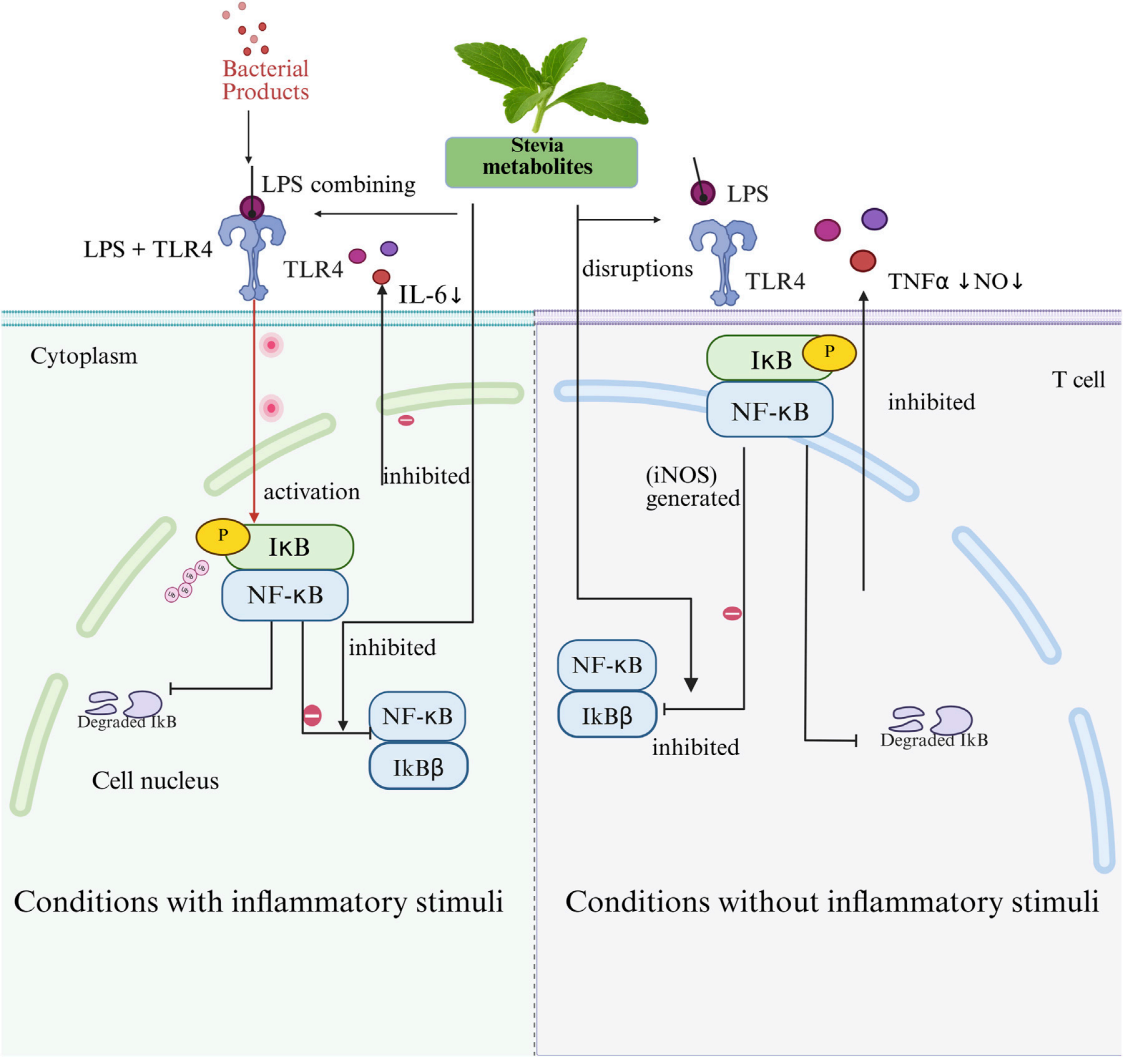
*S. rebaudiana* inhibits the NF-κB pathway to exert anti-inflammatory effects were created using BioRender.

However, traditional treatments for inflammation often involve the use of antibiotics, corticosteroids, and other medications (Zou et al., 2020). Early or excessive use of antibiotics can disrupt the gut microbiota, increasing the risk of diseases such as asthma and allergic rhinitis (Mitre et al., 2018). The use of antibiotics significantly reduces the diversity of gut and lung microbiota, disrupting the bidirectional regulation of the gut-lung axis (Hufnagl et al., 2020), thereby increasing susceptibility to respiratory diseases (Zeng and Liang, 2022). *S. rebaudiana* leaves possess antioxidant, anti-inflammatory, antimicrobial, and immunomodulatory properties, with minimal impact on gut microbiota balance, making them suitable for treating inflammatory conditions in children (Luo et al., 2024). In animal models of inflammatory stimulation, Stevia extracts have been shown to downregulate the inhibitor of kappa B alpha (IκBα)/NF-κB signaling pathway, inhibit the activation of IκB kinase β (IKKβ) and NF-κB, and suppress the expression of inflammatory cytokines, including IL-6. These actions effectively interfere with inflammation triggered by stimuli, including lipopolysaccharide (LPS) (Boonkaewwan et al., 2006; Arya et al., 2012; Boonkaewwan and Burodom, 2013). In the absence of external stimuli, *S. rebaudiana* extracts modulated the inflammatory response of THP-1 cells and under specific conditions, suppressed the excessive release of TNF-α and NO. This process disrupted the binding of Toll-like receptor 4 (TLR4) to LPS and inhibited downstream signaling pathways (Boonkaewwan et al., 2006). The mechanism underlying this process is illustrated in Figure 9.

In TCM, *S. rebaudiana* is used to relieve dryness, alleviate cough, clear heat, detoxify, and enhance immune function. Modern pharmacological studies have also supported these properties. In pediatric *mycoplasma* pneumonia and bronchial asthma, phenolic metabolites of *S. rebaudiana* have been shown to reduce airway inflammation and improve respiratory symptoms (Cheng and She, 2021; Wang et al., 2023). In the treatment of recurrent respiratory infections and recurrent purulent tonsillitis in children, S. rebaudiana has demonstrated immunomodulatory effects, enhancing the respiratory tract’s resistance to infection and inhibiting pathogen growth (Boling et al., 2020; Mao et al., 2023; Raghu and Velayudhannair, 2023; Guo et al., 2024; Yang et al., 2024).

In children with rhinitis and cough-variant asthma, phenolic metabolites have been shown to improve sinus inflammation and reduce cough (Liu et al., 2021; Xu and Zhao, 2023). In the treatment of primary immune thrombocytopenic purpura, S. rebaudiana has been shown to improve medication adherence and exert immunomodulatory effects (Li et al., 2024).

Overall, owing to its natural origin and low side-effect profile, *S. rebaudiana* provides an effective and safe treatment option for inflammatory diseases in children, particularly for the long-term management of chronic inflammatory conditions in pediatric populations.

#### 7.1.3 Prevention and treatment of pediatric obesity

Childhood and adolescent obesity have been increasingly recognized as global health problems. By 2016, over 340 million children and adolescents aged 5–19 years worldwide were overweight or obese (Chien et al., 2023). Overweight and obesity among school-aged children are argued to impact their physical and mental health, academic performance, and quality of life (Liu et al., 2022). Controlling the intake of sugary beverages is a key measure for preventing childhood obesity (Liu et al., 2022). The WHO recommends that, ideally, the intake of free sugars in children and adolescents should be limited to less than 5% of the total energy intake (WHO, 2022a).

Compared to common artificial non-nutritive sweeteners, stevia is a natural sweetener that is not metabolized by the human body (Samuel et al., 2018) and has a minimal biological impact on hypothalamic cells, thus avoiding the central leptin resistance associated with obesity (Park et al., 2019). A long-term study of 1,893 children and adolescents aged 6–15 years confirmed that regular consumption of stevia does not increase body fat content, suggesting that stevia can be used as a sugar substitute to reduce the risk of obesity in childhood (Ding et al., 2016; Chien et al., 2023).

Furthermore, stevia reduces appetite without increasing food intake or postprandial blood glucose levels. This suggests that stevia could be a valuable strategy for the prevention and management of obesity and diabetes (Farhat et al., 2019; Stamataki et al., 2020; WHO, 2022a).

#### 7.1.4 Benefits for gut health

In traditional Chinese medicine, *S. rebaudiana* leaves are believed to have multiple benefits, including nourishing the yin, replenishing body fluids, tonifying the spleen and stomach, and promoting bowel movement. Modern pharmacological studies have gradually verified these traditional uses and revealed the impact of its bioactive metabolites on gut functions.

In children, the gut microbiota undergoes significant changes during early life, influenced by factors such as maternal diet, mode of delivery, infant feeding practices, and antibiotic use. A healthy gut microbiota is essential for maintaining growth and health in children, whereas dysbiosis can lead to issues such as allergies, obesity, and metabolic disorders (Korpela et al., 2017; Mitre et al., 2018). The gut microbiota plays a crucial role in the body by regulating enzyme secretion, metabolite production (e.g., short-chain fatty acids (SCFAs)), and influencing hormone levels (e.g., insulin-like growth factor 1 (IGF-1), Peptide YY (PYY), and glucagon-like peptide 1 (GLP-1)), as well as regulating immune responses and inflammation (e.g., lipopolysaccharide (LPS)) (Niu et al., 2020).

WeiKe is a phytochemically bioactive metabolites mixture isolated from *S. rebaudiana*, with major constituents including curcumin (27.26%), encecalin (14.22%), altholactone (8.28%), protocatechuic aldehyde (8.24%), 4-nitrocatechol (7.67%), syringic acid (7.38%), and phenol (5.07%) (Luo et al., 2024). Studies have demonstrated that WeiKe can effectively modulate gut dysbiosis induced by a high-fat high-fructose diet (HFFD), significantly reducing the Firmicutes/Bacteroidetes ratio and thereby ameliorating obesity and metabolic disorders (Luo et al., 2024). Additionally, stevia significantly increases the abundance of *Lactobacillus* and Akkermansia, further promoting gut health and maintaining the integrity of the intestinal mucosal barrier (Luo et al., 2024). These effects align closely with traditional Chinese medicine theory, which suggests that stevia promotes spleen and stomach health and facilitates bowel movements.

Steviosides and their analogs also show potential as antidiarrheal agents. Steviol, a major metabolite, plays a vital role in regulating intestinal ion transport, particularly in its effects on intestinal Cl^−^, thereby contributing to its therapeutic potential (Brahmachari et al., 2010). Steviosides can also inhibit excessive contraction of the intestinal smooth muscle, alleviating diarrhea caused by hypermotility, especially in conditions such as irritable bowel syndrome and inflammatory bowel disease (Gantait et al., 2015).

Moreover, its antioxidant properties help improve and maintain the integrity of the intestinal barrier, optimize nutrient absorption, and reduce inflammation-induced damage to the gut, supporting its use in promoting gut health (Mehmood et al., 2019; Xu et al., 2023).

#### 7.1.5 Neurological and cognitive support

Depression, attention-deficit/hyperactivity disorder (ADHD), epilepsy, and tic disorders are common neurological conditions in children. These disorders are often accompanied by oxidative stress and excessive neuroinflammatory responses (El Nashar et al., 2022). Natural bioactive metabolites in *S. rebaudiana* exhibit significant anti-inflammatory and antioxidant properties, which help mitigate neuroinflammation, scavenge free radicals, and reduce oxidative damage to neurons, thereby contributing to the protection of the pediatric nervous system (Slykerman et al., 2023; Waris et al., 2024). Notably, *S. rebaudiana* extracts have been shown to cross the blood–brain barrier (Nunes et al., 2007), enhancing their accessibility and potential efficacy in the treatment of central nervous system disorders (Uyanikgil et al., 2016).

In the treatment of depression, *S. rebaudiana* alleviates mood disorders by modulating the activation of the NLRP3 inflammasome and improving microglial cell function (Chavushyan et al., 2017). Moreover, inhibiting the release of NADPH oxidase helps to stabilize neural networks and ameliorate neurological dysfunction caused by metabolic disorders (Du et al., 2024).

In epilepsy and neuroinflammatory diseases such as encephalitis and febrile seizures, *S. rebaudiana* suppresses astrocyte proliferation, inhibits the NF-κB signaling pathway, and reduces excessive intracellular calcium influx. These actions significantly lower the release of pro-inflammatory cytokines, such as TNF-α and IL-6, thereby alleviating neuroinflammation and improving seizure symptoms (Nunes et al., 2007; Uyanikgil et al., 2016; El Nashar et al., 2022).

In addition, children with ADHD and tic disorders are generally more sensitive to the adverse effects of conventional medications (Cortese et al., 2018). The metabolites of *S. rebaudiana* can modulate the dopaminergic system, suppress excessive dopamine receptor activation, and reduce neuroinflammation, effectively improving attention, controlling impulsive behavior, and alleviating tic symptoms (Essoe et al., 2019; Afonso et al., 2020; Salinas-Velarde et al., 2021).

In the TCM system, *S. rebaudiana* has been incorporated into compound herbal formulas for the treatment of pediatric tic disorders. It is commonly combined with Chrysanthemum morifolium (to clear heat and suppress the hyperactive liver), Arisaema cum bile (to resolve phlegm and calm endogenous wind), and Paeonia suffruticosa (to cool the blood and disperse stasis) as core couplet medicines (Lu et al., 2021). However, these prescriptions are largely based on empirical use and preliminary research, and their possible underlying mechanisms lack systematic pharmacological validation. The potential synergistic effects may include multi-target anti-inflammatory action, sedative and tranquilizing effects, and the regulation of neurotransmitter metabolism. Further pharmacological investigations and clinical trials are warranted to validate their efficacy as monotherapy or adjunctive treatments in pediatric neuropsychiatric disorders, particularly in patients sensitive to adverse drug reactions or requiring long-term management.

Therefore, *S. rebaudiana* holds promise as an adjuvant therapeutic strategy or natural alternative medication for pediatric neuropsychiatric patients who require long-term treatment and are sensitive to adverse effects.

### 7.2 Pharmacological potential of *S. rebaudiana* in adults

#### 7.2.1 Lipid regulation and cardiovascular health

*S. rebaudiana* promotes cardiovascular health through multiple activities, including the regulation of blood lipids and blood pressure, and exhibits anti-inflammatory and antioxidant properties. Its polyphenolic and other antioxidant metabolites effectively reduce free radical damage (Olas, 2022) and inhibit the mitogen-activated protein kinase (MAPK) signaling pathway, lowering the levels of inflammation markers associated with atherosclerosis, such as IL-6 and MCP-1, thereby delaying arteriosclerosis progression (Rojas et al., 2018). Furthermore, steviosides reduce inflammation and oxidative stress while promoting the activation of satellite cells and muscle regeneration by inhibiting the NF-κB signaling pathway, particularly in the recovery from heart muscle injury caused by cardiotoxicity (Bunprajun et al., 2012).

Steviosides also alleviate cardiac fibrosis by inhibiting the TGF-β/Smad pathway and regulating the protein expression of MMP2/9 and TIMP2/4. When used in combination with insulin, steviosides demonstrate enhanced reversal effects, providing new evidence of their efficacy in the treatment of diabetic heart complications (Zhao et al., 2020).

In cardiovascular disease treatment, *S. rebaudiana* metabolites typically exhibit only mild side effects (Melis, 1992; Onakpoya and Heneghan, 2015; Villegas Vílchez et al., 2022). Compared to common antihypertensive drugs, *S. rebaudiana* metabolites not only show a significant cardioprotective effect but also exhibit lower nephrotoxicity (Rizwan et al., 2020). Moreover, *S. rebaudiana* metabolites do not induce dose-dependent liver dysfunction or muscle damage, which are commonly associated with statin use (Ilias et al., 2021). Overall, *S. rebaudiana* leaves and their extracts exhibit significant potential for improving cardiovascular health in adults, with evidence supporting their application as natural cardiovascular protectants in humans.

#### 7.2.2 Management of diabetes mellitus

Diabetes mellitus is a chronic metabolic disease characterized by impaired glucose metabolism due to defects in insulin secretion or action, resulting in disruption of carbohydrate, lipid, and protein metabolism (Galicia-Garcia et al., 2020). Although *S. rebaudiana* is widely recognized as a natural sweetener, its use as a natural hypoglycemic herbal remedy for diabetes has garnered increasing attention in the past few years. *S. rebaudiana* metabolites can activate sweet taste receptors (T1R2/T1R3) in the intestine, promoting glucose absorption and insulin secretion (Meyer-Gerspach et al., 2016). The polyphenolic metabolites in *S. rebaudiana* enhance the activity of hepatic glucokinase, facilitating glucose utilization and glycogen storage while inhibiting hepatic glucose output, thereby contributing to stable blood glucose levels (Myint et al., 2020). Additionally, *S. rebaudiana* metabolites inhibit the activity of digestive enzymes, such as α-amylase and α-glucosidase, which slows the digestion and absorption of carbohydrates, thereby further assisting in the control of blood glucose levels (Myint et al., 2023).

Moreover, studies have confirmed that metabolites in *S. rebaudiana*, including steviophethanoside, 6-O-acetyl-(12R)-epiblumdane, and rebaudioside IX, act directly on Insulinoma Cell Line-1 (INS-1) cell lines to activate GLUT4, significantly promoting insulin secretion (Bhasker et al., 2015; Prakash et al., 2017; Ruiz-Ruiz et al., 2017; He et al., 2019; Kang et al., 2022). This mechanism has been validated in diabetic rat models, where steviol glycosides activate the phosphatidylinositol 3-kinase (PI3K) signaling pathway, regulate the translocation of GLUT4, and enhance cellular glucose uptake (Ghanta et al., 2007).

In response to oxidative stress and organ damage caused by hyperglycemia, S. rebaudiana metabolites protect pancreatic β-cell function and survival by inhibiting apoptosis and necrosis pathways associated with diabetes (Ghanta et al., 2007; Oudbor et al., 2022). Moreover, research has indicated that these polyphenols effectively alleviate oxidative stress and organ damage caused by hyperglycemia (Myint et al., 2020).

Furthermore, *S. rebaudiana* metabolites modulate antioxidant signaling pathways in the kidneys (Nrf2/Keap1) and the expression of aquaporin-2 (AQP2), alleviating metabolic disturbances and renal damage induced by diabetes (Bayat et al., 2020). The Suprol complex in Stevia also interacts with Nox enzymes, modulating intracellular redox signaling and impacting glucose metabolism and NF-κB-mediated inflammation (Isoyan et al., 2019).

**FIGURE 10:**
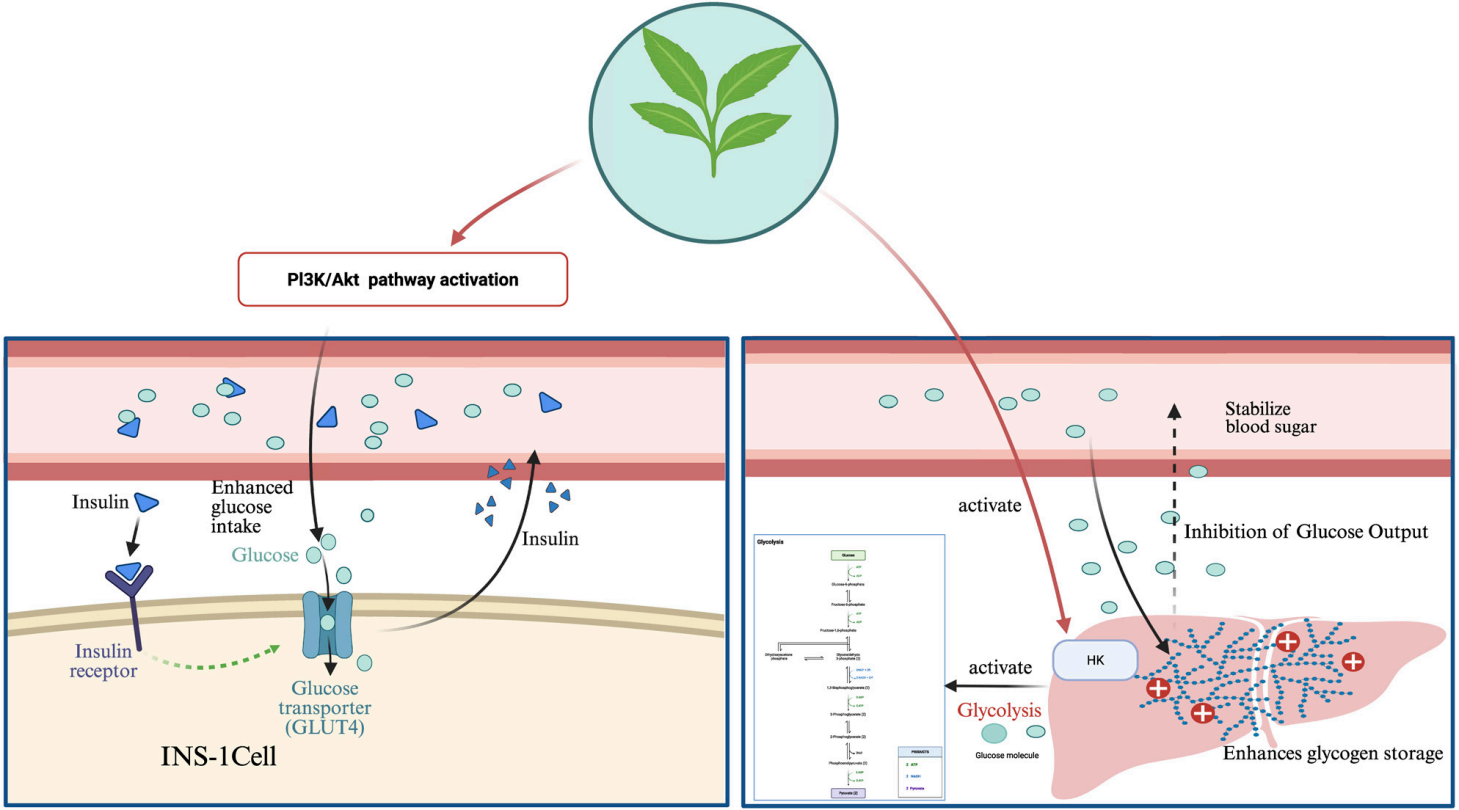
Hypoglycemic effects of *Stevia rebaudiana* through the activation of the PI3K/Akt pathway and regulation of hepatic glucose metabolism were created using BioRender.

**FIGURE 11:**
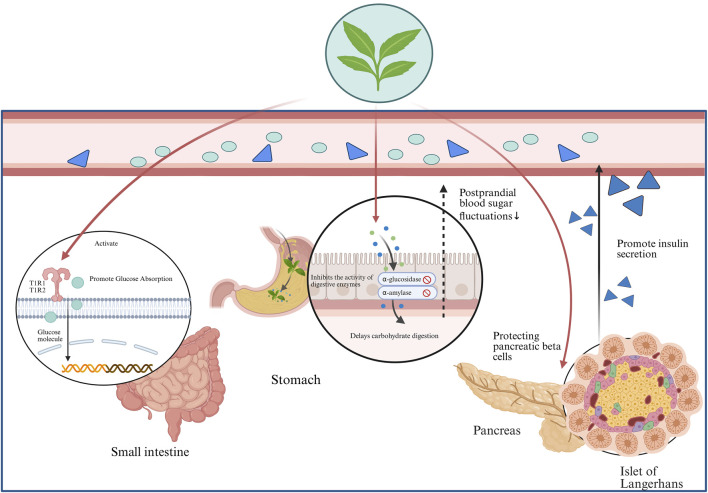
Mechanisms of Stevia rebaudiana in regulating postprandial glucose levels via modulation of intestinal absorption, enzyme inhibition and pancreatic β-cell protection were created using BioRender.

Additionally, *S. rebaudiana* metabolites inhibit the formation of advanced glycation end products (AGEs) and reverse DNA damage caused by glycation (Shahu et al., 2023). Under hyperglycemic conditions, a stevia-enriched diet reduces the number of cytotoxic T cells and pro-inflammatory cytokines (TNF-α and IL-1β) in peripheral circulation, thereby modulating the inflammatory processes associated with diabetes (Cebeci et al., 2024). Figures 10, 11 illustrate the multi-targeted mechanisms by which *S. rebaudiana* regulates glucose metabolism.

The incorporation of nanotechnology has further enhanced the delivery and efficacy of steviosides, offering promising avenues for future antidiabetic strategies (Sudhakar et al., 2021).

#### 7.2.3 Hepatoprotective and nephroprotective effects

In terms of hepatoprotection, *S. rebaudiana* metabolites promote the degradation of intracellular lipid droplets through the activation of autophagy mediated by Sirt1, AMPK, and PPARα, thereby reducing lipid accumulation, hepatic steatosis, and improving liver function (Mei et al., 2022; Park et al., 2022b). Additionally, they regulate the gene expression of phosphoenolpyruvate carboxykinase (PEPCK) and glucokinase (GCK), significantly decreasing the expression of the insulin receptor (INSR) in the liver, thereby improving glucose metabolism and mitigating diabetes-induced liver cell apoptosis (Mousavi-Niri et al., 2023). Furthermore, *S. rebaudiana* metabolites effectively prevent experimental liver cirrhosis by modulating NF-κB, Nrf2, TGF-β, and Smad7 and activating hepatic stellate cells (Ramos-Tovar et al., 2018).

For kidney protection, *S. rebaudiana* metabolites inhibit the formation of angiotensin II, thereby reducing glomerular pressure (Melis, 1996) and regulating water and electrolyte balance, promoting sodium and water excretion (Melis, 1999a), and effectively alleviating the renal burden induced by hypertension. *S. rebaudiana* metabolites also improve renal tubular necrosis, protein casts, and diabetic kidney tissue damage caused by insulin dysregulation (Shivanna et al., 2013; Rizwan et al., 2019). By activating the AMPK and Sirt1 signaling pathways, *S. rebaudiana* metabolites enhance autophagy, thereby improving the self-repair capacity of the kidneys (Mehmood et al., 2022). Moreover, steviosides can activate PPARγ to reduce the protein expression of NF-κB, TGF-β1, Smad2/3, p-Smad2/3, and p-STAT3 in NRK-52E cells, thereby inhibiting LPS-induced epithelial-mesenchymal transition, providing pharmacological evidence for their application in preventing and treating kidney fibrosis (Shen et al., 2022).

Overall, *S. rebaudiana* leaves show promising potential for hepatoprotective and nephroprotective applications, particularly in the treatment of liver and kidney diseases associated with oxidative stress, inflammation, and metabolic disorders.

## 8 Advantages of *S. rebaudiana* over artificial sweeteners

A wide range of non-nutritive sweeteners are currently in use, including acesulfame potassium, aspartame, advantame, saccharin, neotame, cyclamate, sucralose, and natural alternatives such as steviol glycosides (WHO, 2022b; Chen et al., 2024). To assess the unique advantages of *S. rebaudiana*, particularly its thermal stability and compatibility with compound herbal formulas in traditional decoctions, this study makes a comprehensive comparison of the physicochemical properties and biological activities of various sweeteners (Table 9).

**TABLE 9:** Comparative assessment of *S*. *rebaudiana* and synthetic non-nutritive sweeteners.

Category	Sweetness (relative to sucrose)	Advantages	Disadvantages	Ref.
Aspartame	200x	Widely used in beverages and low-calorie foods	Heat-labile; unsuitable for baking; contraindicated in phenylketonuria	(Chattopadhyay et al., 2014; Farag et al., 2022)
Stevioside	200–300x	Naturally derived Contains bioactive metabolites with potential health benefits; heat stable; gut-friendly; Sweet and neutral in TCM properties, suitable for a wide range of populations	May exhibit a bitter or metallic aftertaste at high concentrations	(Goyal et al., 2010; Chattopadhyay et al., 2014; Arumugam et al., 2020)
Sucralose	450–650x	Highly heat stable; not metabolized by the human body	Potential gut microbiota disruption; insufficient long-term data	Chattopadhyay et al. (2014)
Saccharin	300–400 x	Low-cost; heat stable	May produce an unpleasant metallic or bitter taste at high concentrations; historical carcinogenic concerns remain controversial	Chattopadhyay et al. (2014)
Acesulfame K	200x	High stability: Heat-resistant and acid-resistant, suitable for a wide range of applications.	May be perceived as bitter or metallic	Chattopadhyay et al. (2014)
Advantame	20,000x	Extremely high sweetness; highly stable	Contraindicated in phenylketonuria	(Ruiz-Ojeda et al., 2019; Mora and Dando, 2021; Farag et al., 2022)
		High stability: Suitable for a wide range of applications	Low market penetration: Limited to a smaller range of applications	
Cyclamate	30–50x	Heat stable; low-cost; broadly applicable	Health risks; Previously banned in some countries due to suspected links to cancer risk Poor taste: Sometimes has a slightly bitter or metallic aftertaste	(Ruiz-Ojeda et al., 2019; Mora and Dando, 2021)
Neotame	7,000–13,000x	Heat-resistant and acid-resistant; widely applicable Contains phenylalanine: Does not affect individuals with phenylketonuria.	Low market penetration: Despite broad applications, it has lower recognition compared to other sweeteners.	(Ruiz-Ojeda et al., 2019; Mora and Dando, 2021; Farag et al., 2022)

This table summarizes the sweetness intensity, advantages, and limitations of common Artificial Sweeteners. Glycosides from *S*. *rebaudiana*, derived from traditional Chinese botanical drugs, have unique advantages in terms of safety, thermal stability, and cultural compatibility with traditional decoction methods.

Compared to commonly used artificial sweeteners, steviol glycosides have several notable advantages. First, as natural plant-derived metabolites, they align with consumer preferences for “natural and healthy” products and are culturally compatible, particularly in the form of TCM preparations. With a neutral sweet flavor and mild thermal properties, steviol glycosides do not interfere with the therapeutic nature of herbal formulas. They also exhibit excellent thermal stability and water solubility, allowing them to retain both sweetness and bioactivity during high-temperature decoction processes (Jahan et al., 2010). Additionally, stevia has minimal impact on the gut microbiota and possesses antioxidant, hypoglycemic, and anti-inflammatory properties, making it suitable for long-term use in patients with chronic conditions and improving overall treatment adherence.

In contrast, many artificial sweeteners, despite their high sweetness intensity and cost efficiency, have limitations such as thermal instability (e.g., aspartame), potential safety concerns (e.g., saccharin and cyclamate), and adverse effects on gut microbial balance (e.g., sucralose) (Chattopadhyay et al., 2014; Ruiz-Ojeda et al., 2019; Mora and Dando, 2021; Farag et al., 2022). Although some artificial sweeteners, such as acesulfame potassium and neotame, are thermally stable, their lack of cultural relevance and limited regulatory support in TCM contexts restrict their application in traditional medicine systems.

Among all widely used non-nutritive sweeteners, *S. rebaudiana* extracts are derived from medicinal and edible traditional herbs, giving them a unique advantage in integrated medicine (Harshita, 2023; Yeung, 2023). They exhibit good thermal and aqueous stability, making them ideal for herbal decoctions and functional foods, while providing health-promoting bioactivities (Jahan et al., 2010). Moreover, from a TCM perspective, steviol glycosides are considered sweet and neutral in nature, and are thus suitable for a wide range of individuals.

Taken together, based on the comparative characteristics summarized in Table 9, steviol glycosides emerge as a superior functional sweetener because of their natural origin, thermal stability, biofunctional properties, safety profile, and compatibility with both cultural and medicinal contexts. These qualities underscore their promising potential for inclusion in dietary recommendations and standardization of traditional medicine-based prescriptions.

## 9 Safety evaluation

In recent years, the widespread application of *S*. *rebaudiana* as a natural sweetener has led to sustained attention to its safety. Major international regulatory agencies, including the WHO and EFSA, have confirmed its safe use in both food and pharmaceutical contexts (Orellana-Paucar, 2023). The acceptable daily intake for steviol glycosides has been established at 4 mg/kg body weight, with a permitted impurity level of up to 5% (Younes et al., 2020). In China, the Ministry of Health approved its use as a pharmaceutical excipient as early as 1990 (Ding et al., 2016). The safety of *S. rebaudiana* has been supported by a large volume of clinical and toxicological research, including over 40,000 clinical trials conducted in Japan (Goyal et al., 2010).

From a pharmacokinetic perspective, steviol glycosides are not hydrolyzed in the upper gastrointestinal tract but are metabolized by the intestinal microbiota into steviol, which is rapidly excreted in the urine without systemic accumulation. This metabolic route significantly reduces potential toxicity and is consistent across both children and adults (Samuel et al., 2018; Salehi et al., 2019; Purkayastha and Kwok, 2020; Orellana-Paucar, 2023).

Genotoxicity assessments have indicated that *S*. *rebaudiana* and its metabolites are non-mutagenic and non-genotoxic (Suttajit et al., 1993; Uçar et al., 2018). Long-term carcinogenicity studies in rodents have not demonstrated tumorigenic potential (Chappell et al., 2021). In a 90-day subchronic toxicity test, even exposure to doses 300-fold higher than the recommended ADI did not result in significant adverse effects (Zhang et al., 2017). Similarly, oral administration of steviol glycosides at doses up to 1,880 mg/kg for 4 weeks caused only mild increases in oxidative damage and chromosomal abnormalities in mice, whereas no genotoxic effects were observed at doses as high as 8,000 mg/kg *in vivo* (Orellana-Paucar, 2023).

However, emerging evidence suggests potential safety concerns under high-dose or long-term exposure conditions. *In vitro* studies have shown that steviol glycosides at concentrations of 10 mg/mL and 50 mg/mL significantly induced DNA damage in human lymphocytes, with damage levels approximately 62% higher than those in the negative-control group. In addition, chromatin structural changes, such as karyopyknosis, were observed, indicating a potential genotoxic effect (Pasqualli et al., 2020). Animal studies have also indicated reproductive toxicity, including reduced spermatogenesis and decreased seminal vesicle weight (Melis, 1999b). Conversely, other studies have reported beneficial effects on male reproductive function, as steviol glycosides may enhance spermatogenesis by activating sweet taste receptors, such as T1R2 and GNAT3 (Shen and Li, 2021; Hanna et al., 2023). As these doses far exceed typical dietary exposure levels, further studies are needed to establish the therapeutic index of steviol glycosides in healthy volunteers, which would help define the upper boundary of their pharmacological applications.

In terms of metabolic toxicity, a 16-week mouse study using human-equivalent doses (HED) revealed that long-term stevia consumption significantly increased HbA1c, liver enzymes (ALT and AST), urea, creatinine, cholesterol, LDL, and free fatty acids. These changes are accompanied by elevated nitric oxide levels and reduced superoxide dismutase (SOD) activity, which are indicative of oxidative stress and insulin resistance (Farid et al., 2020). Additionally, increased water intake and diuretic activity were observed, along with renal inflammation and functional impairment. Steviol, the primary metabolite, may accumulate in the kidneys via entero-renal circulation, imposing further stress on the glomeruli and proximal tubules, consistent with previous findings of stevia-induced nephrotoxicity (Toskulkao et al., 1994). Hepatic histopathology also revealed lobular disorganization, inflammatory infiltration, and disrupted bile secretion (Cardoso et al., 1996; Geuns et al., 2003; Farid et al., 2020).

Regarding immune function, steviol glycosides have been shown to exert dose-dependent immunosuppressive effects. At concentrations of 10–50 μg/mL, CD4^+^ and CD8^+^ T-cell populations were significantly reduced by approximately 35% and 32%, respectively, suggesting disruption of immune homeostasis (Pasqualli et al., 2020). Farid et al. (2020) further demonstrated that stevia intake significantly elevated immunoglobulin levels (IgG, IgE, and IgA) and pro-inflammatory cytokines (IL-6, Interleukin-8 (IL-8)) while reducing anti-inflammatory IL-10, indicating systemic immune activation. Although high-purity stevia metabolites are generally non-allergenic, individuals with known allergic predispositions may exhibit sensitivity in skin prick tests (Urban et al., 2015), warranting caution in those with a documented history of hypersensitivity (Kimata, 2007).

Although most studies emphasize the beneficial effects of Stevia on the gut microbiota, Farid et al. (2020) reported that chronic stevia intake may impair intestinal barrier integrity, enabling the translocation of LPS into the portal circulation, which subsequently activates hepatic innate immune responses and promotes inflammation. Additionally, steviol glycosides may inhibit the growth of beneficial species, such as *Lactobacillus* reuteri, while promoting pathogenic bacteria, thereby disrupting microbial homeostasis (Deniņa et al., 2014).

Furthermore, case reports suggest that *S. rebaudiana* metabolites may cause decreased mean arterial pressure, bradycardia, or hypoglycemia in some individuals (Chatsudthipong and Muanprasat, 2009; Olas, 2022; Orellana-Paucar, 2023). However, these effects have been disputed. In a study by Ray et al. (2020), no significant changes in systolic or diastolic blood pressure were observed after 4 weeks of oral administration of steviol glycoside A at 1,000 mg/kg/day in normotensive or hypotensive individuals. Collectively, most evidence supports that steviol glycosides do not significantly affect blood pressure or glycemic homeostasis within the recommended intake limits (Barriocanal et al., 2008). Furthermore, a case report described a 54-year-old man with obstructive sleep apnea, post-transplant diabetic nephropathy, and peripheral neuropathy who developed moderate restless legs syndrome associated with daily stevia intake (used in coffee). Symptoms resolved upon discontinuation and reappeared with re-exposure (Goswami and Pusalavidyasagar, 2020). This suggests that stevia may interfere with dopaminergic signaling and should be used with particular caution in patients with chronic comorbidities undergoing lifestyle interventions.

In conclusion, current mainstream evidence supports the safety of *S*. *rebaudiana* when used within the recommended intake range. Its favorable metabolic characteristics and non-toxicological profile provide a robust foundation for its application in functional foods and pharmaceutical preparations. Nevertheless, potential adverse effects related to immune modulation, hepatic and renal metabolism, and gut barrier function under high-dose or chronic exposure conditions warrant careful consideration of these factors. Further targeted studies are needed to assess its safety in vulnerable populations and long-term use scenarios, thus contributing to a more comprehensive safety evaluation framework.

## 10 Limitations

Despite the broad pharmacological activities and favorable safety profile exhibited by *S*. *rebaudiana*, current research still faces several critical limitations. On one hand, its multi-target therapeutic potential lacks validation through large-scale, randomized, double-blind, placebo-controlled clinical trials in humans, which hinders the effective translation of preclinical findings into clinical applications. Particularly, clinical evidence remains inadequate for its traditional uses in skin regeneration, anti-fatigue, and immune enhancement, underscoring the urgent need to bridge the gap between basic research and clinical practice. On the other hand, the chemical composition of its active constituents is significantly influenced by multiple factors, such as cultivar, geographical origin, extraction method, and harvest time (Chen et al., 2014; Karaköse et al., 2015; Bao et al., 2024), resulting in considerable pharmacological heterogeneity. Therefore, the establishment of standardized quality evaluation systems and unified dosage guidelines remains essential. In addition, most existing studies have concentrated on a narrow range of species and those of specific geographic sources, which limits the global application and systematic development and utilization of high-quality germplasm resources. To enable the scientific and sustainable development of *S. rebaudiana* and its related species, comprehensive global studies comparing their chemical profiles and pharmacological activities are urgently needed to elucidate the functional relationships among species and to recognize optimal selection strategies (Yi et al., 2012).

From a safety perspective, although international regulatory bodies, such as the EFSA and WHO, have confirmed its safety within the recommended dosage range, some studies have suggested that long-term or high-dose intake may pose potential risks, including immune dysregulation, reproductive toxicity, and hepatic or renal metabolic burdens. These findings highlight the necessity of systematic toxicological assessments and long-term clinical follow-ups, especially in vulnerable populations, such as children, pregnant women, and patients with chronic illnesses. Targeted safety evaluations based on population subgroups, dosage, and treatment duration are imperative.

Furthermore, current research provides limited insights into the pharmacological mechanisms of *S. rebaudiana* in compound medicines. In TCM, *S. rebaudiana* is commonly co-administered with other herbal preparations. However, the synergistic or antagonistic mechanisms of these combinations remain underexplored. Future studies should focus on dissecting metabolite interactions, pharmacological targets, and underlying mechanisms within the context of polyherbal interventions to enhance the clinical applicability and mechanistic understanding of *S. rebaudiana* in modern integrative medicine.

## 11 Conclusion and future perspectives

*S. rebaudiana* is a multifunctional medicinal plant with broad pharmacological activities, demonstrating significant application potential in both ethnopharmacology and evidence-based medicine. Its bioactivities, such as antidiabetic, antioxidant, anti-inflammatory, neuroprotective, and gut microbiota-modulating effects, are primarily attributed to its key active constituents, including steviol glycosides, flavonoids, polyphenols, and polysaccharides. In addition, *S. rebaudiana* exhibits favorable water solubility, thermal stability, and safety, making it particularly suitable for use in functional foods, traditional decoctions, and in pediatric medications. Its ability to cross the blood–brain barrier and modulate the gut microbiota further bolsters its potential for treating metabolic, neurological, and inflammatory disorders. Although existing studies have demonstrated the potential pharmacological activities of *S. rebaudiana* in various diseases, systematic clinical evidence regarding its optimal and safe dosage for different indications remains limited (Barriocanal et al., 2008). Therefore, future research should focus on clarifying the dose–response relationship, particularly in contexts such as functional foods, pediatric applications, and chronic disease management, to establish scientifically grounded and rational dosage recommendations.

In addition, future studies should focus on multi-omics integration, elucidation of synergistic mechanisms among key constituents, and pharmacokinetic profiling, particularly through advanced technologies such as UPLC-DAD-QTOF-MS, bioassay-guided screening, and computer-aided drug design (Yi et al., 2010; Yi et al., 2012; Chen et al., 2017; Chen et al., 2021). In-depth investigations into the *in vivo* behavior of composite phytochemicals, including extraction, absorption, biotransformation, tissue distribution, metabolism, and target engagement, are needed to advance new drug development for metabolic diseases, neurodegenerative disorders, and chronic inflammation.

Moreover, it is recommended that future research expand the diversity of biological materials by including samples from different geographical and genetic backgrounds, in order to systematically evaluate how environmental and germplasm variability influences phytochemical composition and pharmacological efficacy. This will help optimize breeding strategies and establish a robust quality control system for *S. rebaudiana*.

In summary, future research on *S. rebaudiana* should evolve toward the integration of multisource materials, multidimensional pharmacological mechanisms, multi-omics platforms, and multicomponent pharmacokinetics. Such approaches will enhance its scientific value and clinical potential as a functional food and natural therapeutic agent, while laying a theoretical foundation and shaping a practical framework for its standardized development, global application, and cross-cultural integration into modern medicine.

## Glossary

ADHD: Attention Deficit Hyperactivity Disorder
AGEs: Advanced Glycation End Products
AHP: Analytic Hierarchy Process
ALT: Alanine Aminotransferase
AMPK: Adenosine Monophosphate-Activated Protein Kinase
AST: Aspartate Aminotransferase
AQP2: Aquaporin-2
CAT: Catalase
EFSA: European Food Safety Authority
GCK: Glucokinase
GLP-1: Glucagon-like Peptide 1
GLUT4: Glucose Transporter Type 4
GSH: Glutathione
GSH-Px: Glutathione Peroxidase
HPLC: High-Performance Liquid Chromatography
HSV-1: Herpes Simplex Virus Type 1
HSV-2: Herpes Simplex Virus Type 2
IGF-1: Insulin-like Growth Factor 1
IL-1β: Interleukin-1 Beta
IL-6: Interleukin-6
IL-8: Interleukin-8
IκBα: Inhibitor of kappa B alpha
IKKβ: IκB kinase β
INSR: Insulin Receptor
JECFA: Joint FAO/WHO Expert Committee on Food Additives
LPS: Lipopolysaccharide
MAPK: Mitogen-Activated Protein Kinase
MDA: Malondialdehyde
NADPH: Nicotinamide Adenine Dinucleotide Phosphate
NF-κB: Nuclear Factor Kappa-Light-Chain-Enhancer of Activated B Cells
NO: Nitric Oxide
Nox: NADPH Oxidase
Nrf2/Keap1: Nuclear Factor Erythroid 2-Related Factor 2/Kelch-like ECH-Associated Protein 1
PEPCK: Phosphoenolpyruvate Carboxykinase
PPARγ: Peroxisome Proliferator-Activated Receptor Gamma
PYY: Peptide YY
PI3K/Akt: Phosphoinositide 3-kinase/Protein Kinase B
ROS: Reactive Oxygen Species
SCFA: Short-Chain Fatty Acids
SEM: Scanning Electron Microscopy
Sirt1: Sirtuin 1
SOD: Superoxide Dismutase
SREBP-1c: Sterol Regulatory Element-Binding Protein 1c
TCM: Traditional Chinese Medicine
TGF-β: Transforming Growth Factor Beta
TNF-α: Tumor Necrosis Factor Alpha
VEGF: Vascular Endothelial Growth Factor
WHO: World Health Organization

## References

[B1] Abdel-Aal R. A. Abdel-Rahman M. S. Ali L. (2019). Stevia improves the antihyperglycemic effect of metformin in streptozotocin-induced diabetic rats: a novel strategy in type 2 diabetes mellitus. Bull. Pharm. Sci. Assiut Univ. 42 (1), 39–50. 10.21608/bfsa.2019.62264

[B2] Afonso G. J. M. Silva J. B. Santos R. M. Rosário L. M. Quinta-Ferreira R. M. Quinta-Ferreira M. E. (2020). ROS changes evoked by the natural sweetener rebaudioside A in a neuronal system. Energy Rep. 6, 909–914. 10.1016/j.egyr.2019.12.003

[B3] Aghababaei F. Hadidi M. (2023). Recent advances in potential health benefits of quercetin. Pharmaceuticals 16 (7), 1020. 10.3390/ph16071020 37513932 PMC10384403

[B4] Aghajanyan A. Movsisyan Z. Trchounian A. (2017). Antihyperglycemic and antihyperlipidemic activity of hydroponic Stevia rebaudiana aqueous extract in hyperglycemia induced by immobilization stress in rabbits. Biomed. Res. Int. 2017, 9251358. 10.1155/2017/9251358 28758125 PMC5512021

[B5] Albani C. M. Borgo J. Fabbri J. Pensel P. Paladini A. Beer M. F. (2022). Antiparasitic effects of asteraceae Species extracts on Echinococcus granulosus s.s. Evid. Based Complement. Altern. Med. 2022, 6371849. 10.1155/2022/6371849 36193140 PMC9526667

[B6] Alessandrini E. Brako F. Scarpa M. Lupo M. Bonifazi D. Pignataro V. (2021). Children's preferences for oral dosage forms and their involvement in formulation research *via* EPTRI (European paediatric translational research infrastructure). Pharmaceutics 13 (5), 730. 10.3390/pharmaceutics13050730 34063499 PMC8156390

[B7] Ali A. Kant K. Gupta S. Kaur N. Jindal P. Naeem M. (2024). “Chapter 6 - enhancing nutritional and antidiabetic properties of Stevia rebaudiana bert.—A sweet-leaf plant through various scientific approaches,” in Antidiabetic medicinal plants. Editors Naeem M. Aftab T. (Academic Press), 229–253.

[B8] Alshawwa S. Z. Mohammed E. J. Hashim N. Sharaf M. Selim S. Alhuthali H. M. (2022). *In situ* biosynthesis of reduced alpha hematite (α-Fe(2)O(3)) nanoparticles by Stevia rebaudiana L. leaf extract: insights into antioxidant, antimicrobial, and anticancer properties. Antibiot. (Basel) 11 (9), 1252. 10.3390/antibiotics11091252 36140030 PMC9495369

[B9] Alves L. R. Borges C. L. Almeida F. (2024). Editorial: global excellence in fungal pathogenesis: central and South America. Front. Cell Infect. Microbiol. 14, 1481806. 10.3389/fcimb.2024.1481806 39324057 PMC11422200

[B10] Andrade J. K. S. Barros R. G. C. Rezende Y. R. R. S. Nogueira J. P. de Oliveira C. S. Gualberto N. C. (2021). Evaluation of bioactive compounds, phytochemicals profile and antioxidant potential of the aqueous and ethanolic extracts of some traditional fruit tree leaves used in Brazilian folk medicine. Food Res. Int. 143, 110282. 10.1016/j.foodres.2021.110282 33992382

[B11] Aran B. Mijeong K. Koeun J. Seulki K. Jeehyun L. Yeong Ok S. (2014). Inhibitory effects of functional sujeonggwa drinks on hepatic lipid accumulation in hypercholesterolemic ApoE knockout mice. J. Korean Soc. Food Sci. Nutr. 43 (11), 1648–1657. 10.3746/jkfn.2014.43.11.1648

[B12] Araya R. Menezes P. R. Claro H. G. Brandt L. R. Daley K. L. Quayle J. (2021). Effect of a digital intervention on depressive symptoms in patients with comorbid hypertension or diabetes in Brazil and Peru: two randomized clinical trials. Jama 325 (18), 1852–1862. 10.1001/jama.2021.4348 33974019 PMC8114139

[B13] Arumugam B. Subramaniam A. Alagaraj P. (2020). Stevia as a natural sweetener: a review. Cardiovasc Hematol. Agents Med. Chem. 18 (2), 94–103. 10.2174/1871525718666200207105436 32031079

[B14] Arya A. Kumar S. Kasana M. (2012). Anti-inflammatory activity of *in vitro* regenerated calli and *in vivo* plant of Stevia rebaudiana (bert.) bertoni. Int. J. Sci. Res. Publ. 2 (8), 435–439. Available online at: http://www.ijsrp.org/research-paper-0812.php?rp=P0787.

[B15] Augustinho do Nascimento C. Kim R. R. Ferrari C. R. de Souza B. M. Braga A. S. Magalhães A. C. (2021). Effect of sweetener containing stevia on the development of dental caries in enamel and dentin under a microcosm biofilm model. J. Dent. 115, 103835. 10.1016/j.jdent.2021.103835 34653536

[B16] Bai H. Zhang Z. Zhu M. Sun Y. Wang Y. Li B. (2024). Research progress of treating hyperuricemia in rats and mice with traditional Chinese medicine. Front. Pharmacol. 15, 1428558. 10.3389/fphar.2024.1428558 39101136 PMC11294118

[B17] Balkrishna A. Sharma I. P. Arya V. Joshi B. Kushwaha A. K. Kumar A. (2025). Ethnomedicinal plant exploration and traditional healthcare practices along the ganga river: a journey from gomukh to gangasagar, India. J. Herbs, Spices and Med. Plants 31, 143–174. 10.1080/10496475.2025.2449877

[B18] Bao Y. Liu C. Shao T. Han J. Wang G. (2024). Progress in the study of chemical components and pharmacological effects of Stevia rebaudiana and its quality markers (Q-Marker) prediction (in Chinese). Chin. Herb. Med. 55 (03), 1014–1025. Available online at: https://next.cnki.net/middle/abstract?v=5l3vb1p_-rh-eNsjYPehCFu-R8Eeknb0mAq6JsZmYFfBEPEYfogx7eKec7k5n32BcP8rkxWI1w6Ny_ChQvGwLEicysFnc0IZ9ynYDJwGTw4J-EL4je3KM-8xnY2QJw-C5NYRQs751hyZRWBblCXhJxbBU4XHJF-dOa1zijz4lB_PLE8Fd8sgzQ4cKs5ef2KW6ibb-6ejCjA=&uniplatform=NZKPT&language=CHS&scence=null.

[B19] Barakat H. Al-Roug K. Algonaiman R. Althwab S. A. Alfheeaid H. A. Alhomaid R. M. (2023). Biological assessment of stevioside and sucralose as sucrose substitutes for diabetics on STZ-induced diabetes in rats. Molecules 28 (3), 940. 10.3390/molecules28030940 36770608 PMC9920551

[B20] Barriocanal L. A. Palacios M. Benitez G. Benitez S. Jimenez J. T. Jimenez N. (2008). Apparent lack of pharmacological effect of steviol glycosides used as sweeteners in humans. A pilot study of repeated exposures in some normotensive and hypotensive individuals and in type 1 and type 2 diabetics. Regul. Toxicol. Pharmacol. 51 (1), 37–41. 10.1016/j.yrtph.2008.02.006 18397817

[B21] Bayat E. Rahpeima Z. Dastghaib S. Gholizadeh F. Erfani M. Asadikaram G. (2020). Stevia rebaudiana extract attenuate metabolic disorders in diabetic rats *via* modulation of glucose transport and antioxidant signaling pathways and aquaporin-2 expression in two extrahepatic tissues. J. Food Biochem. 44 (8), e13252. 10.1111/jfbc.13252 32515037

[B22] Beer M. F. Frank F. M. Germán Elso O. Ernesto Bivona A. Cerny N. Giberti G. (2016). Trypanocidal and leishmanicidal activities of flavonoids isolated from Stevia satureiifolia Var. satureiifolia. Pharm. Biol. 54 (10), 2188–2195. 10.3109/13880209.2016.1150304 26983579

[B23] Belda-Galbis C. M. Pina-Pérez M. C. Espinosa J. Marco-Celdrán A. Martínez A. Rodrigo D. (2014). Use of the modified gompertz equation to assess the Stevia rebaudiana bertoni antilisterial kinetics. Food Microbiol. 38, 56–61. 10.1016/j.fm.2013.08.009 24290626

[B24] Benelli G. Pavela R. Drenaggi E. Desneux N. Maggi F. (2020). Phytol, (E)-nerolidol and spathulenol from Stevia rebaudiana leaf essential oil as effective and eco-friendly botanical insecticides against Metopolophium dirhodum. Industrial Crops Prod. 155, 112844. 10.1016/j.indcrop.2020.112844

[B25] Bhasker S. Madhav H. Chinnamma M. (2015). Molecular evidence of insulinomimetic property exhibited by steviol and stevioside in diabetes induced L6 and 3T3L1 cells. Phytomedicine 22 (11), 1037–1044. 10.1016/j.phymed.2015.07.007 26407946

[B26] Bhattacharjee T. Sen S. Chakraborty R. Maurya P. K. Chattopadhyay A. (2020). “Cultivation of medicinal plants: special reference to important medicinal plants of India,” in Herbal medicine in India: indigenous knowledge, practice, innovation and its value. Editors Sen S. Chakraborty R. (Singapore: Springer Singapore), 101–115.

[B27] Boling L. Cuevas D. A. Grasis J. A. Kang H. S. Knowles B. Levi K. (2020). Dietary prophage inducers and antimicrobials: toward landscaping the human gut microbiome. Gut Microbes 11 (4), 721–734. 10.1080/19490976.2019.1701353 31931655 PMC7524278

[B28] Boonkaewwan C. Burodom A. (2013). Anti‐inflammatory and immunomodulatory activities of stevioside and steviol on colonic epithelial cells. J. Sci. Food Agric. 93 (15), 3820–3825. 10.1002/jsfa.6287 23794454

[B29] Boonkaewwan C. Toskulkao C. Vongsakul M. (2006). Anti-inflammatory and immunomodulatory activities of stevioside and its metabolite steviol on THP-1 cells. J. Agric. Food Chem. 54 (3), 785–789. 10.1021/jf0523465 16448183

[B30] Borgo J. Laurella L. C. Martini F. Catalán C. A. N. Sülsen V. P. (2021). Stevia genus: phytochemistry and biological activities update. Molecules 26 (9), 2733. 10.3390/molecules26092733 34066562 PMC8125113

[B31] Brahmachari G. Mandal L. C. Roy R. Mondal S. Brahmachari A. K. (2010). Stevioside and related compounds – molecules of pharmaceutical promise: a critical overview. Arch. Pharm. 344 (1), 5–19. 10.1002/ardp.201000181 21213347

[B32] Brambilla E. Cagetti M. G. Ionescu A. Campus G. Lingström P. (2013). An *in vitro* and *in vivo* comparison of the effect of Stevia rebaudiana extracts on different caries-related variables: a randomized controlled trial pilot study. Caries Res. 48 (1), 19–23. 10.1159/000351650 24216624

[B33] Brinckmann J. A. (2015). Geographical indications for medicinal plants: globalization, climate change, quality and market implications for geo-authentic botanicals. World J. Traditional Chin. Med. 1 (1), 16–23. 10.15806/j.issn.2311-8571.2014.0020

[B34] Bunprajun T. Yimlamai T. Soodvilai S. Muanprasat C. Chatsudthipong V. (2012). Stevioside enhances satellite cell activation by inhibiting of NF-κB signaling pathway in regenerating muscle after cardiotoxin-induced injury. J. Agric. Food Chem. 60 (11), 2844–2851. 10.1021/jf203711d 22316332

[B35] Cai T. Ye H. Jiang H. Lin C. Lou C. Wang W. (2023). Stevioside targets the NF-κB and MAPK pathways for inhibiting inflammation and apoptosis of chondrocytes and ameliorates osteoarthritis *in vivo* . Int. Immunopharmacol. 115, 109683. 10.1016/j.intimp.2023.109683 36630751

[B36] Cardoso V. N. Barbosa M. F. Muramoto E. Mesquita C. H. Almeida M. A. (1996). Pharmacokinetic studies of 131I-stevioside and its metabolites. Nucl. Med. Biol. 23 (1), 97–100. 10.1016/0969-8051(95)02072-1 9004922

[B37] Carmo C. D. S. Ribeiro M. R. C. Teixeira J. X. P. Alves C. M. C. Franco M. M. França A. (2018). Added sugar consumption and chronic oral disease burden among adolescents in Brazil. J. Dent. Res. 97 (5), 508–514. 10.1177/0022034517745326 29342369

[B38] Cebeci E. Katirci E. Karhan M. Korgun E. T. (2024). The immunomodulator effect of Stevia rebaudiana bertoni mediated by TNF‐α and IL‐1β in peripheral blood in diabetic rats. Food Sci. and Nutr. 12, 7581–7590. 10.1002/fsn3.4371 39479688 PMC11521730

[B39] Celaya L. Martina P. Kolb-Koslobsky N. (2022). Infusions prepared with stevia rebaudiana: application of a simplex centroid mixture design for the study of natural sweeteners and phenolic compounds. J. Food Sci. Technol. 59 (1), 55–64. 10.1007/s13197-021-04979-9 35068551 PMC8758884

[B40] Ceole L. F. Companhoni M. V. P. Sanches Lopes S. M. de Oliveira A. J. B. Goncalves R. A. C. Dias Filho B. P. (2020). Anti-herpes activity of polysaccharide fractions from Stevia rebaudiana leaves. Nat. Prod. Res. 34 (11), 1558–1562. 10.1080/14786419.2018.1516662 30580608

[B41] Cevasco Contreras M. D.P. Borgo J. Celentano A. M. Elso O. G. Bach H. Catalán C. A. N. (2024). Extracts and terpenoids from stevia species as potential anthelmintics for neglected tropical diseases caused by cestode parasites. Molecules 29 (18), 4430. 10.3390/molecules29184430 39339424 PMC11433760

[B42] Chaiyana W. Charoensup W. Sriyab S. Punyoyai C. Neimkhum W. (2021). Herbal extracts as potential antioxidant, anti‐aging, anti-inflammator Y, and whitening cosmeceutical ingredients. Chem. and Biodivers. 18 (7), e2100245. 10.1002/cbdv.202100245 33989453

[B43] Chakma A. Afrin F. Rasul M. G. Maeda H. Yuan C. Shah A. K. M. A. (2023). Effects of extraction techniques on antioxidant and antibacterial activity of stevia (Stevia rebaudiana bertoni) leaf extracts. Food Chem. Adv. 3, 100494. 10.1016/j.focha.2023.100494

[B44] Chan P. Tomlinson B. Chen Y. J. Liu J. C. Hsieh M. H. Cheng J. T. (2000). A double-blind placebo-controlled study of the effectiveness and tolerability of oral stevioside in human hypertension. Br. J. Clin. Pharmacol. 50 (3), 215–220. 10.1046/j.1365-2125.2000.00260.x 10971305 PMC2014988

[B45] Chappell G. A. Heintz M. M. Borghoff S. J. Doepker C. L. Wikoff D. S. (2021). Lack of potential carcinogenicity for steviol glycosides - systematic evaluation and integration of mechanistic data into the totality of evidence. Food Chem. Toxicol. 150, 112045. 10.1016/j.fct.2021.112045 33587976

[B46] Chatsudthipong V. Muanprasat C. (2009). Stevioside and related compounds: therapeutic benefits beyond sweetness. Pharmacol. and Ther. 121 (1), 41–54. 10.1016/j.pharmthera.2008.09.007 19000919

[B47] Chattopadhyay S. Raychaudhuri U. Chakraborty R. (2014). Artificial sweeteners - a review. J. Food Sci. Technol. 51 (4), 611–621. 10.1007/s13197-011-0571-1 24741154 PMC3982014

[B48] Chavushyan V. A. Simonyan K. V. Simonyan R. M. Isoyan A. S. Simonyan G. M. Babakhanyan M. A. (2017). Effects of stevia on synaptic plasticity and NADPH oxidase level of CNS in conditions of metabolic disorders caused by fructose. BMC Complement. Altern. Med. 17 (1), 540. 10.1186/s12906-017-2049-9 29258552 PMC5735878

[B49] Chaweekulrat P. Kanokrungsee S. Viriyaskultorn N. Prasertsook S. Likittanasombat S. Boonchai W. (2025). Sensitive skin in thais: prevalence, clinical characteristics, and diagnostic cutoff scores. J. Cosmet. Dermatol 24 (4), e70181. 10.1111/jocd.70181 40211966 PMC11986799

[B50] Chen Z. Ye S.-Y. (2022). Research progress on antiviral constituents in traditional Chinese medicines and their mechanisms of action. Pharm. Biol. 60 (1), 1063–1076. 10.1080/13880209.2022.2074053 35634712 PMC9154771

[B51] Chen Q. Yi T. Tang Y. Wong L. L. Huang X. Zhao Z. (2014). Comparative authentication of three “snow lotus” herbs by macroscopic and microscopic features. Microsc. Res. Tech. 77 (8), 631–641. 10.1002/jemt.22381 24841997

[B52] Chen J. M. Ding L. Sui X. C. Xia Y. M. Wan H. D. Lu T. (2016). Production of a bioactive sweetener steviolbioside *via* specific hydrolyzing ester linkage of stevioside with a β-galactosidase. Food Chem. 196, 155–160. 10.1016/j.foodchem.2015.09.035 26593477

[B53] Chen Q.-L. Zhu L. Tang Y.-N. Kwan H.-Y. Zhao Z.-Z. Chen H.-B. (2017). Comparative evaluation of chemical profiles of three representative 'snow lotus' herbs by UPLC-DAD-QTOF-MS combined with principal component and hierarchical cluster analyses. Drug Test. Analysis 9 (8), 1105–1115. 10.1002/dta.2123 27764538

[B54] Chen J. Xia Y. Sui X. Peng Q. Zhang T. Li J. (2018). Steviol, a natural product inhibits proliferation of the gastrointestinal cancer cells intensively. Oncotarget 9 (41), 26299–26308. 10.18632/oncotarget.25233 29899860 PMC5995179

[B55] Chen Q. Zhu L. Yip K. M. Tang Y. Liu Y. Jiang T. (2021). A hybrid platform featuring nanomagnetic ligand fishing for discovering COX-2 selective inhibitors from aerial part of Saussurea laniceps hand.-mazz. J. Ethnopharmacol. 271, 113849. 10.1016/j.jep.2021.113849 33485983

[B56] Chen N. Cao W. Yuan Y. Wang Y. Zhang X. Chen Y. (2024). Recent advancements in mogrosides: a review on biological activities, synthetic biology, and applications in the food industry. Food Chem. 449, 139277. 10.1016/j.foodchem.2024.139277 38608607

[B57] Cheng W. She J. (2021). Application of Gao Fang in children with bronchial asthma. (in Chinese). J. Tradit. Chin. Med. 62 (11), 1006–1007+1012. 10.13288/j.11-2166/r.2021.11.017

[B58] Chien Y. H. Lin C. Y. Hsu S. Y. Chen Y. H. Wu H. T. Huang S. W. (2023). Effects of nonnutritive sweeteners on body composition changes during pubertal growth. Nutrients 15 (10), 2319. 10.3390/nu15102319 37242202 PMC10224528

[B59] Chowdhury A. I. Rahanur Alam M. Raihan M. M. Rahman T. Islam S. Halima O. (2022). Effect of stevia leaves (Stevia rebaudiana bertoni) on diabetes: a systematic review and meta-analysis of preclinical studies. Food Sci. Nutr. 10 (9), 2868–2878. 10.1002/fsn3.2904 36171777 PMC9469865

[B60] Cordeiro M. S. Simas D. L. R. Pérez-Sabino J. F. Mérida-Reyes M. S. Muñoz-Wug M. A. Oliva-Hernández B. E. (2020). Characterization of the antinociceptive activity from Stevia serrata cav. Biomedicines 8 (4), 79. 10.3390/biomedicines8040079 32272558 PMC7235832

[B61] Cortese S. Adamo N. Del Giovane C. Mohr-Jensen C. Hayes A. J. Carucci S. (2018). Comparative efficacy and tolerability of medications for attention-deficit hyperactivity disorder in children, adolescents, and adults: a systematic review and network meta-analysis. Lancet Psychiatry 5 (9), 727–738. 10.1016/S2215-0366(18)30269-4 30097390 PMC6109107

[B62] Danpanichkul P. Suparan K. Dutta P. Kaeosri C. Sukphutanan B. Pang Y. (2024). Disparities in metabolic dysfunction-associated steatotic liver disease and cardiometabolic conditions in low and lower middle-income countries: a systematic analysis from the global burden of disease study 2019. Metabolism - Clin. Exp. 158, 155958. 10.1016/j.metabol.2024.155958 38942169

[B63] Das K. (2013). Wound healing potential of aqueous crude extract of Stevia rebaudiana in mice. Rev. Bras. Farmacogn. 23 (2), 351–357. 10.1590/S0102-695X2013005000011

[B64] Dave M. (2024). EBD spotlight: the prevalence of root caries in the Indian population. BDJ Team 11 (1), 36–37. 10.1038/s41407-024-2057-9

[B65] de Souza P. Mariano L. N. B. Cechinel-Zanchett C. C. Cechinel-Filho V. (2020). Promising medicinal plants with diuretic potential used in Brazil: state of the art, challenges, and prospects. Planta Med. 87 (01/02), 24–37. 10.1055/a-1257-0887 32957146

[B66] Demirci-Cekic S. Ozkan G. Avan A. N. Uzunboy S. Capanoglu E. Apak R. (2022). Biomarkers of oxidative stress and antioxidant defense. J. Pharm. Biomed. Anal. 209, 114477. 10.1016/j.jpba.2021.114477 34920302

[B67] Deniņa I. Semjonovs P. Fomina A. Treimane R. Linde R. (2014). The influence of stevia glycosides on the growth of Lactobacillus reuteri strains. Lett. Appl. Microbiol. 58 (3), 278–284. 10.1111/lam.12187 24251876

[B68] Di Ianni M. E. Del Valle M. E. Enrique A. V. Rosella M. A. Bruno F. Bruno-Blanch L. E. (2015). Computer-aided identification of anticonvulsant effect of natural nonnutritive sweeteners stevioside and rebaudioside A. Assay. Drug Dev. Technol. 13 (6), 313–318. 10.1089/adt.2015.29010.meddrrr 26258457 PMC4533089

[B69] Ding H. Hong L. Zhao B. Liu C. Xing J. Zhu X. (2016). Main applications and current production issues of stevia rebaudiana. (in Chinese). Chin. Sugar Crops. 38 (06), 77–78+80. 10.13570/j.cnki.scc.2016.06.026

[B70] Du L. Fan X. Yang Y. Wu S. Liu Y. (2024). Quercetin ameliorates cognitive impairment in depression by targeting HSP90 to inhibit NLRP3 inflammasome activation. Mol. Neurobiol. 61 (9), 6628–6641. 10.1007/s12035-024-03926-x 38329680

[B71] Duarte M. C. T. Figueira G. M. Sartoratto A. Rehder V. L. G. Delarmelina C. (2005). Anti-candida activity of Brazilian medicinal plants. J. Ethnopharmacol. 97 (2), 305–311. 10.1016/j.jep.2004.11.016 15707770

[B72] Echavarría N. G. Echeverría L. E. Stewart M. Gallego C. Saldarriaga C. (2021). Chagas disease: chronic chagas cardiomyopathy. Curr. Probl. Cardiol. 46 (3), 100507. 10.1016/j.cpcardiol.2019.100507 31983471

[B73] EFSA Panel on Food Additives and Nutrient Sources added to Food (2010). Scientific opinion on the safety of steviol glycosides for the proposed uses as a food additive. EFSA J. 8 (4), 1537. 10.2903/j.efsa.2010.1537

[B74] El Nashar E. M. Obydah W. Alghamdi M. A. Saad S. Yehia A. Maryoud A. (2022). Effects of Stevia rebaudiana bertoni extracts in the rat model of epilepsy induced by pentylenetetrazol: sirt-1, at the crossroads between inflammation and apoptosis. J. Integr. Neurosci. 21 (1), 21. 10.31083/j.jin2101021 35164457

[B75] El-Hadary A. Sitohy M. (2021). Safely effective hypoglycemic action of stevia and turmeric extracts on diabetic albino rats. J. Food Biochem. 45 (1), e13549. 10.1111/jfbc.13549 33161596

[B76] Escobar E. Piedrahita M. Gregory R. L. (2020). Growth and viability of Streptococcus mutans in sucrose with different concentrations of Stevia rebaudiana bertoni. Clin. Oral Investig. 24 (9), 3237–3242. 10.1007/s00784-020-03197-5 32189073

[B77] Esquivel-García R. Pérez-Calix E. Ochoa-Zarzosa A. García-Pérez M. E. (2018). Flora etnomedicinal utilizada para el tratamiento de afecciones dermatológicas en la Meseta Purépecha, Michoacán, México. Acta Bot. Mex. (125), 95–132. 10.21829/abm125.2018.1339

[B78] Essoe J. K. Grados M. A. Singer H. S. Myers N. S. McGuire J. F. (2019). Evidence-based treatment of Tourette's disorder and chronic tic disorders. Expert Rev. Neurother. 19 (11), 1103–1115. 10.1080/14737175.2019.1643236 31295410

[B79] Farag M. A. Rezk M. M. Hamdi Elashal M. El-Araby M. Khalifa S. A. M. El-Seedi H. R. (2022). An updated multifaceted overview of sweet proteins and dipeptides as sugar substitutes; the chemistry, health benefits, gut interactions, and safety. Food Res. Int. 162 (Pt A), 111853. 10.1016/j.foodres.2022.111853 36461268

[B80] Farhat G. Berset V. Moore L. (2019). Effects of Stevia extract on postprandial glucose response, satiety and energy intake: a three-arm crossover trial. Nutrients 11 (12), 3036. 10.3390/nu11123036 31842388 PMC6950708

[B81] Farid A. Hesham M. El-Dewak M. Amin A. (2020). The hidden hazardous effects of stevia and sucralose consumption in male and female albino mice in comparison to sucrose. Saudi Pharm. J. 28 (10), 1290–1300. 10.1016/j.jsps.2020.08.019 33132722 PMC7584803

[B82] Ferri L. A. Alves-Do-Prado W. Yamada S. S. Gazola S. Batista M. R. Bazotte R. B. (2006). Investigation of the antihypertensive effect of oral crude stevioside in patients with mild essential hypertension. Phytother. Res. 20 (9), 732–736. 10.1002/ptr.1944 16775813

[B83] Galicia-Garcia U. Benito-Vicente A. Jebari S. Larrea-Sebal A. Siddiqi H. Uribe K. B. (2020). Pathophysiology of Type 2 diabetes Mellitus. Int. J. Mol. Sci. 21 (17), 6275. 10.3390/ijms21176275 32872570 PMC7503727

[B84] Gantait S. Das A. Mandal N. (2015). Stevia: a comprehensive review on ethnopharmacological properties and *in vitro* regeneration. Sugar Tech. 17 (2), 95–106. 10.1007/s12355-014-0316-3

[B85] Gao Y. Fan Y. Liu L. Chen S. Li S. Pan F. (2021). Study on quality evaluation of Stevia Rebaudiana decoction pieces based on quality criterion model of effective reference characteristic spectrum(in Chinese). Glob. Tradit. Chin. Med. 14 (05), 777–788. Available online at: https://kns.cnki.net/kcms2/article/abstract?v=SyD34uTtguCjnMM7TJfDONeE8qFXRecut0nzLdiUDL5_M3LM1ND0MpD_8NvxMPExengpTty2mFsY9RV-T4758LUFxGn0m_VHU5m1TAA1KrbjdVPAe_YTMb5twABg9vpG2UiIBUL2Zr6llpeYiiJf7BZV1gUVj-ythIejTMvavau0OGmPSjHpXFl-uFBXKlryW8VMNl_jdYA=&uniplatform=NZKPT&language=CHS.

[B86] Gatea F. Sârbu I. Vamanu E. (2021). *In vitro* modulatory effect of stevioside, as a partial sugar replacer in sweeteners, on human child Microbiota. Microorganisms 9 (3), 590. 10.3390/microorganisms9030590 33805627 PMC8000329

[B87] Geuns J. M. Augustijns P. Mols R. Buyse J. G. Driessen B. (2003). Metabolism of stevioside in pigs and intestinal absorption characteristics of stevioside, rebaudioside A and steviol. Food Chem. Toxicol. 41 (11), 1599–1607. 10.1016/s0278-6915(03)00191-1 12963013

[B88] Ghanta S. Banerjee A. Poddar A. Chattopadhyay S. (2007). Oxidative DNA damage preventive activity and antioxidant potential of Stevia rebaudiana (Bertoni) bertoni, a natural sweetener. J. Agric. Food Chem. 55 (26), 10962–10967. 10.1021/jf071892q 18038982

[B89] Gorain S. Pal J. Biswas S. J. (2024). “Chapter 3 - an ethnobotanical survey on the treatment of diabetes by tribal traditional healers of purulia district, West Bengal, India,” in Antidiabetic medicinal plants. Editors Naeem M. Aftab T. (Academic Press), 141–166.

[B90] Goswami U. Pusalavidyasagar S. (2020). Restless legs syndrome associated with use of stevia nonnutritive sweetener. J. Clin. Sleep. Med. 16 (10), 1819–1821. 10.5664/jcsm.8702 32691724 PMC7953997

[B91] Goyal S. K. Samsher Goyal R. K. (2010). Stevia (Stevia rebaudiana) a bio-sweetener: a review. Int. J. Food Sci. Nutr. 61 (1), 1–10. 10.3109/09637480903193049 19961353

[B92] Gregersen S. Jeppesen P. B. Holst J. J. Hermansen K. (2004). Antihyperglycemic effects of stevioside in type 2 diabetic subjects. Metabolism - Clin. Exp. 53 (1), 73–76. 10.1016/j.metabol.2003.07.013 14681845

[B93] Guo C. Wang M. Li X. (2014). Quality criteria of folium steviae rebaudianae (in Chinese). Chin. J. Exp. Traditional Med. Formulae 20 (14), 91–94. 10.13422/j.cnki.syfjx.2014140091

[B94] Guo Z. Cui X. Li W. Liu Y. Zhao J. (2024). Discussion on the differentiation and treatment of recurrent suppurative tonsillitis in children based on “heat, stasis, phlegm, and deficiency” (in Chinese). Mod. Clin. Tradit. Chin. Med., 1–5. Available online at: https://link.cnki.net/urlid/10.1157.R.20241009.1427.022

[B95] Gupta E. Kaushik S. Purwar S. Sharma R. Balapure A. K. Sundaram S. (2017). Anticancer potential of steviol in MCF-7 human breast cancer cells. Pharmacogn. Mag. 13 (51), 345–350. 10.4103/pm.pm_29_17 28839355 PMC5551348

[B96] Han J.-Y. Park M. Lee H.-J. (2023). Stevia (Stevia rebaudiana) extract ameliorates insulin resistance by regulating mitochondrial function and oxidative stress in the skeletal muscle of db/db mice. BMC Complementary Med. Ther. 23 (1), 264. 10.1186/s12906-023-04033-5 37488560 PMC10367355

[B97] Han H.-J. Ko M. N. Shin C. S. Hyun C.-G. (2024). Human health benefits and microbial consortium of stevia fermented with barley nuruk. Fermentation 10 (7), 330. 10.3390/fermentation10070330

[B98] Hanna D. H. Beshay S. N. El Shafee E. El-Rahman H. A. A. (2023). The protective effect of aqueous extract of Stevia rebaudiana against tartrazine toxicity in male wistar rat. Cell Biochem. Funct. 41 (8), 1462–1476. 10.1002/cbf.3886 38010705

[B99] Harshita K. (2023). Monk fruit (Siraitia grosvenorii): a comprehensive review of its sweetness, health benefits, and applications as a natural sweetener. Pharma Innovation J. 12 (6), 3007–3012. Available online at: https://api.semanticscholar.org/CorpusID:268364630.

[B100] He J. Zhu N. L. Kong J. Peng P. Li L. F. Wei X. L. (2019). A newly discovered phenylethanoid glycoside from Stevia rebaudiana bertoni affects insulin secretion in rat INS-1 islet β cells. Molecules 24 (22), 4178. 10.3390/molecules24224178 31752141 PMC6891645

[B101] Hsieh M. H. Chan P. Sue Y. M. Liu J. C. Liang T. H. Huang T. Y. (2003). Efficacy and tolerability of oral stevioside in patients with mild essential hypertension: a two-year, randomized, placebo-controlled study. Clin. Ther. 25 (11), 2797–2808. 10.1016/s0149-2918(03)80334-x 14693305

[B102] Hufnagl K. Pali-Scholl I. Roth-Walter F. Jensen-Jarolim E. (2020). Dysbiosis of the gut and lung microbiome has a role in asthma. Semin. Immunopathol. 42 (1), 75–93. 10.1007/s00281-019-00775-y 32072252 PMC7066092

[B103] Hurrell J. A. Puentes J. P. (2013). Medicinal and aromatic species of asteraceae commercialized in the conurbation buenos Aires-La plata (argentina). Ethnobiol. Conservation 2 (0). 10.15451/ec2013-8-2.7-1-40

[B104] Hurrell J. A. Puentes J. P. Arenas P. M. (2015). Medicinal plants with cholesterol-lowering effect marketed in the buenos Aires-La plata conurbation, Argentina: an urban ethnobotany study. Ethnobiol. Conservation 4 (0). 10.15451/ec2015-9-4.7-1-19

[B105] Ilias N. Hamzah H. Ismail I. S. Mohidin T. B. M. Idris M. F. Ajat M. (2021). An insight on the future therapeutic application potential of Stevia rebaudiana bertoni for atherosclerosis and cardiovascular diseases. Biomed. Pharmacother. 143, 112207. 10.1016/j.biopha.2021.112207 34563950

[B106] Isoyan A. S. Simonyan K. V. Simonyan R. M. Babayan M. A. Simonyan G. M. Chavushyan V. A. (2019). Superoxide-producing lipoprotein fraction from stevia leaves: definition of specific activity. BMC Complement. Altern. Med. 19 (1), 88. 10.1186/s12906-019-2500-1 31023287 PMC6485107

[B107] Jahan I. A. Mostafa M. Hossain H. Nimmi I. Sattar M. Alim A. (2010). Antioxidant activity of Stevia rebaudiana bert. Leaves from Bangladesh. Bangladesh Pharm. J. 13, 6–10. Available online at: https://www.academia.edu/26008439/Antioxidant_activity_of_Stevia_rebaudiana_Bert_Leaves_from_Bangladesh.

[B108] Jayaraman S. Manoharan M. S. Illanchezian S. (2008). *In-vitro* antimicrobial and antitumor activities of Stevia rebaudiana (asteraceae) leaf extracts. Trop. J. Pharm. Res. 7 (4), 1143–1149. 10.4314/tjpr.v7i4.14700

[B109] JECFA (2008). Steviol glycosides (E960) specifications and safety evaluation. Available online at: https://apps.who.int/food-additives-contaminants-jecfa-database/chemical.aspx?chemID=267.

[B110] JECFA (2010). Joint FAO/WHO Expert Committee on Food Additives. Compendium of Food Additive Specifications. FAO JECFA Monographs 10. Rome, Italy: Food and Agriculture Organization of the United Nations.

[B111] Kang H. Lee D. Kang K. S. Kim K. H. (2022). A new labdane-type diterpene, 6-O-Acetyl-(12R)-epiblumdane, from Stevia rebaudiana leaves with insulin secretion effect. Biomedicines 10 (4), 839. 10.3390/biomedicines10040839 35453589 PMC9026343

[B112] Karaköse H. Müller A. Kuhnert N. (2015). Profiling and quantification of phenolics in Stevia rebaudiana leaves. J. Agric. Food Chem. 63 (41), 9188–9198. 10.1021/acs.jafc.5b01944 26333998

[B113] Kaundal R. S. Pandey T. Pandey V. (2024). Exploring the therapeutic potential of quercetin: mitigating neuroinflammation. Neurosci. Behav. Physiology 54, 1082–1097. 10.1007/s11055-024-01640-8

[B114] Khakpai F. Naseroleslami M. Moheb-Alian M. Ghanimati E. Abdollah-pour F. Mousavi-Niri N. (2023). Intra-gastrically administration of Stevia and particularly nano-stevia reversed the hyperglycemia, anxiety, and memory impairment in streptozotocin-induced diabetic rats. Physiology and Behav. 263, 114100. 10.1016/j.physbeh.2023.114100 36716984

[B115] Khare N. Chandra S. (2019). Stevioside mediated chemosensitization studies and cytotoxicity assay on breast cancer cell lines MDA-MB-231 and SKBR3. Saudi J. Biol. Sci. 26 (7), 1596–1601. 10.1016/j.sjbs.2018.10.009 31762632 PMC6864384

[B116] Khatun M. C. S. Muhit M. A. Hossain M. J. Al-Mansur M. A. Rahman S. M. A. (2021). Isolation of phytochemical constituents from Stevia rebaudiana (bert.) and evaluation of their anticancer, antimicrobial and antioxidant properties *via in vitro* and *in silico* approaches. Heliyon 7 (12), e08475. 10.1016/j.heliyon.2021.e08475 34917793 PMC8645449

[B117] Kim I.-S. Yang M. Lee O.-H. Kang S.-N. (2011). The antioxidant activity and the bioactive compound content of Stevia rebaudiana water extracts. LWT - Food Sci. Technol. 44 (5), 1328–1332. 10.1016/j.lwt.2010.12.003

[B118] Kim D. H. Jeong C. H. Han S. G. Jung H. S. Han S. G. (2023). Stevia extract enhances the fermentation and functional properties of fermented milk in human Colon epithelial cells. Food Biosci. 53, 102747. 10.1016/j.fbio.2023.102747

[B119] Kimata H. (2007). Anaphylaxis by stevioside in infants with atopic eczema. Allergy 62 (5), 565–566. 10.1111/j.1398-9995.2007.01317.x 17441798

[B120] Kinki A. Mezgebe A. G. Bekele T. (2021). Evaluation of dried stevia (Stevia rebaudiana bertoni) leaf and its infusion nutritional profile. 10.35248/2167-0412.20.9.360

[B121] Koc S. Isgor B. S. Isgor Y. G. Shomali Moghaddam N. Yildirim O. (2015). The potential medicinal value of plants from asteraceae family with antioxidant defense enzymes as biological targets. Pharm. Biol. 53 (5), 746–751. 10.3109/13880209.2014.942788 25339240

[B122] Korpela K. Zijlmans M. A. Kuitunen M. Kukkonen K. Savilahti E. Salonen A. (2017). Childhood BMI in relation to microbiota in infancy and lifetime antibiotic use. Microbiome 5 (1), 26. 10.1186/s40168-017-0245-y 28253911 PMC5335838

[B123] Kujawska M. (2018). Yerba mate (Ilex paraguariensis) beverage: nutraceutical ingredient or conveyor for the intake of medicinal plants? Evidence from Paraguayan folk medicine. Evidence-Based Complementary Altern. Med. 2018 (1), 6849317. 10.1155/2018/6849317 29725356 PMC5872613

[B124] Kujawska M. Schmeda-Hirschmann G. (2022). The use of medicinal plants by Paraguayan migrants in the Atlantic forest of Misiones, Argentina, is based on Guaraní tradition, colonial and current plant knowledge. J. Ethnopharmacol. 283, 114702. 10.1016/j.jep.2021.114702 34627987

[B125] Kurek J. M. Mikołajczyk-Stecyna J. Krejpcio Z. (2023). Steviol glycosides from Stevia rebaudiana bertoni mitigate lipid metabolism abnormalities in diabetes by modulating selected gene expression – an *in vivo* study. Biomed. and Pharmacother. 166, 115424. 10.1016/j.biopha.2023.115424 37677968

[B126] Lemus-Mondaca R. Vega-Gálvez A. Zura-Bravo L. Ah-Hen K. (2012). Stevia rebaudiana bertoni, source of a high-potency natural sweetener: a comprehensive review on the biochemical, nutritional and functional aspects. Food Chem. 132 (3), 1121–1132. 10.1016/j.foodchem.2011.11.140 29243591

[B127] Lemus-Mondaca R. Vega-Gálvez A. Rojas P. Stucken K. Delporte C. Valenzuela-Barra G. (2018). Antioxidant, antimicrobial and anti-inflammatory potential of Stevia rebaudiana leaves: effect of different drying methods. J. Appl. Res. Med. Aromatic Plants 11, 37–46. 10.1016/j.jarmap.2018.10.003

[B128] Lemus-Mondaca R. Zura-Bravo L. Ah-Hen K. Di Scala K. (2021). Effect of drying methods on drying kinetics, energy features, thermophysical and microstructural properties of Stevia rebaudiana leaves. J. Sci. Food Agric. 101 (15), 6484–6495. 10.1002/jsfa.11320 34000065

[B129] Li Z. An L. Zhang S. Shi Z. Bao J. Tuerhong M. (2021). Structural elucidation and immunomodulatory evaluation of a polysaccharide from Stevia rebaudiana leaves. Food Chem. 364, 130310. 10.1016/j.foodchem.2021.130310 34237616

[B130] Li N. Li X. Deng M. Zhu F. Wang Z. Sheng R. (2023a). Isosteviol derivatives as protein tyrosine Phosphatase-1B inhibitors: synthesis, biological evaluation and molecular docking. Bioorg. and Med. Chem. 83, 117240. 10.1016/j.bmc.2023.117240 36963270

[B131] Li Z. Song S. Yang J. Zeng J. Liu X. (2023b). Study on HPLC characteristic chromatogram of Stevia rebaudiana extract(in Chinese). Feed Res. 46 (24), 105–111. 10.13557/j.cnki.issn1002-2813.2023.24.021

[B132] Li S. Yu H. Pan Y. Li M. Jiang M. (2024). Clinical experience in treating primary immune thrombocytopenia with high-dose mulberry leaves. (in Chinese). Mod. Clin. Tradit. Chin. Med., 1–6. Available online at: https://link.cnki.net/urlid/10.1157.r.20241009.1430.030

[B133] Liu Q. Pan Y. Wu W. (2018). Review on chemical compositions and pharmacological activities of Stevia rebaudiana(bertoni) hemsl.(in Chinese). Nat. Prod. Res. Dev. 10.16333/j.1001-6880.2018.6.027

[B134] Liu Z. Wang J. Xu H. Chang S. Ruan Y. (2021). Highlights of ruan Yan's experience in treating pediatric chronic rhinitis. (in Chinese). Chin. J. Tradit. Chin. Med. 36 (04), 2133–2135. Available online at: https://kns.cnki.net/kcms2/article/abstract?v=k15566fjT2mhR4-ERXa9F8Vr01Rg44kg4PglZB-5cw0rHMofFKPpjuZK1T1aueF9n3VmY2rs03e6QiHamnmBPy0Y7ExQWiOFEs7jfHBokIPZUJNuN9ONfLNqVDCPdgz2U_7mplmpVxrPpA2DHYuVMr6CQ_5S0iwGVBb4zGbzrnx2XgKt-mUg0HiTTOOpCfajCT2XDqn8k0Y=&uniplatform=NZKPT&language=CHS.

[B135] Liu Z. Gao P. Gao A. Y. Lin Y. Feng X. X. Zhang F. (2022). Effectiveness of a multifaceted intervention for prevention of obesity in primary school children in China: a cluster randomized clinical trial. JAMA Pediatr. 176 (1), e214375. 10.1001/jamapediatrics.2021.4375 34747972 PMC8576631

[B136] Lopez V. Perez S. Vinuesa A. Zorzetto C. Abian O. (2016). Stevia rebaudiana ethanolic extract exerts better antioxidant properties and antiproliferative effects in tumour cells than its diterpene glycoside stevioside. Food Funct. 7 (4), 2107–2113. 10.1039/c5fo01586c 27071804

[B137] Lu Q. Wang L. M. Huang M. Zhang M. J. Liu Z. H. Yang Y. (2021). Analysis of medication patterns in the treatment of tic disorders by chief physician Huang mao. J. Hebei Tradit. Chin. Med. 36 (05), 52–56. 10.16370/j.cnki.13-1214/r.2021.05.015

[B138] Luo G. Zhao Y. Wang X. Yin M. (2024). Flavonoid‐enriched extract from stevia (Stevia rebaudiana bertoni) regulated lipid accumulation and gut microbiota in obese mice. Int. J. Food Sci. and Technol. 59 (9), 6065–6077. 10.1111/ijfs.17329

[B139] Mahalak K. K. Firrman J. Tomasula P. M. Nuñez A. Lee J. J. Bittinger K. (2020). Impact of steviol glycosides and erythritol on the human and *Cebus apella* gut microbiome. J. Agric. Food Chem. 68 (46), 13093–13101. 10.1021/acs.jafc.9b06181 31869223

[B140] Maneenoon K. Khuniad C. Teanuan Y. Saedan N. Prom-in S. Rukleng N. (2015). Ethnomedicinal plants used by traditional healers in Phatthalung province, peninsular Thailand. J. Ethnobiol. Ethnomedicine 11 (1), 43. 10.1186/s13002-015-0031-5 26025447 PMC4469324

[B141] Manthattil Vysyan S. Suraj Prasanna M. Jayanandan A. Gangadharan A. K. Chittalakkottu S. (2024). Phytocompounds hesperidin, rebaudioside a and rutin as drug leads for the treatment of tuberculosis targeting mycobacterial phosphoribosyl pyrophosphate synthetase. J. Biomol. Struct. Dyn., 1–15. 10.1080/07391102.2024.2438363 39659199

[B142] Mao N. Li J. Wu F. (2023). Medication patterns based on real-world studies of Wu Fanwei's treatment of cough diseases using the six meridians theory. (in Chinese). New Tradit. Chin. Med. 55 (17), 32–40. 10.13457/j.cnki.jncm.2023.17.005

[B143] Marchyshyn S. Pasyechko N. Slobodianiuk L. BudnІAk L. Kozyr G. Khomitska A. (2023). Study of sugar-lowering activity of dry extract from stevia leaves. Fitoterapia (4), 48–56. 10.32782/2522-9680-2023-4-48

[B144] Marcinek K. Krejpcio Z. (2016). Stevia rebaudiana bertoni: health promoting properties and therapeutic applications. J. für Verbraucherschutz und Lebensmittelsicherheit 11 (1), 3–8. 10.1007/s00003-015-0968-2

[B145] Maryam N. N. Nangyal H. Ali L. Rashid A. (2015). Screening of leaf extracts of Stevia rebaudiana forAntibacterial activity, phytotoxic and haemagglutination activities. American-Eurasian J.Agric.and Environ. 10.5829/idosi.aejaes.2015.15.10.10165

[B146] Máthé Á. (2015). “Introduction: utilization/significance of medicinal and aromatic plants,” in Medicinal and aromatic plants of the world: scientific, production, commercial and utilization aspects. Editor Máthé Á. (Dordrecht, Netherlands: Springer), 1–12.

[B147] Mayasari N. R. Susetyowati Wahyuningsih M. S. H. Probosuseno (2018). Antidiabetic effect of rosella-stevia tea on prediabetic women in yogyakarta, Indonesia. J. Am. Coll. Nutr. 37 (5), 373–379. 10.1080/07315724.2017.1400927 29425471

[B148] Mehmood A. Zhao L. Wang C. Hossen I. Raka R. N. Zhang H. (2019). Stevia residue extract increases intestinal uric acid excretion *via* interactions with intestinal urate transporters in hyperuricemic mice. Food Funct. 10 (12), 7900–7912. 10.1039/c9fo02032b 31789332

[B149] Mehmood A. Zhao L. Ishaq M. Xin W. Zhao L. Wang C. (2020). Anti-hyperuricemic potential of stevia (Stevia rebaudiana bertoni) residue extract in hyperuricemic mice. Food Funct. 11 (7), 6387–6406. 10.1039/c9fo02246e 32613954

[B150] Mehmood A. Althobaiti F. Zhao L. Usman M. Chen X. Alharthi F. (2022). Anti-inflammatory potential of stevia residue extract against uric acid-associated renal injury in mice. J. Food Biochem. 46 (10), e14286. 10.1111/jfbc.14286 35929489

[B151] Mei Y. Kuai Y. Hu H. Liu F. Liu B. Sun X. (2020). Isosteviol sodium attenuates high fat/high cholesterol-induced kidney dysfunction by inhibiting inflammation, oxidative stress and apoptosis. Biol. Pharm. Bull. 43 (8), 1172–1178. 10.1248/bpb.b19-01028 32741937

[B152] Mei Y. Hu H. Deng L. Sun X. Tan W. (2022). Therapeutic effects of isosteviol sodium on non-alcoholic fatty liver disease by regulating autophagy *via* Sirt1/AMPK pathway. Sci. Rep. 12 (1), 12857. 10.1038/s41598-022-16119-0 35896572 PMC9329321

[B153] Melis M. S. (1992). Stevioside effect on renal function of normal and hypertensive rats. J. Ethnopharmacol. 36 (3), 213–217. 10.1016/0378-8741(92)90046-t 1434679

[B154] Melis M. S. (1996). A crude extract of Stevia rebaudiana increases the renal plasma flow of normal and hypertensive rats. Braz J. Med. Biol. Res. 29 (5), 669–675. Available online at: https://pubmed.ncbi.nlm.nih.gov/9033821/. 9033821

[B155] Melis M. S. (1999a). Effect of crude extract of Stevia rebaudiana on renal water and electrolytes excretion. Phytomedicine 6 (4), 247–250. 10.1016/s0944-7113(99)80016-6 10589443

[B156] Melis M. S. (1999b). Effects of chronic administration of Stevia rebaudiana on fertility in rats. J. Ethnopharmacol. 67 (2), 157–161. 10.1016/s0378-8741(99)00081-1 10619379

[B157] Melis M. S. Rocha S. T. Augusto A. (2009). Steviol effect, a glycoside of Stevia rebaudiana, on glucose clearances in rats. Braz J. Biol. 69 (2), 371–374. 10.1590/s1519-69842009000200019 19675940

[B158] Mendoza-Pérez S. Orta-Méndez-y-Sánchez I. García-Gómez R. S. Ordaz-Nava G. Gracia-Mora M. I. Macías-Rosales L. (2024). Stevia rebaudiana bertoni, an American plant used as sweetener: study of its effects on body mass control and glycemia reduction in wistar m ale and female rats. PLOS ONE 19 (2), e0298251. 10.1371/journal.pone.0298251 38412182 PMC10898749

[B159] Meyer-Gerspach A. C. Wolnerhanssen B. Beglinger C. (2016). Functional roles of low calorie sweeteners on gut function. Physiol. Behav. 164 (Pt B), 479–481. 10.1016/j.physbeh.2016.01.045 26861179

[B160] Mitre E. Susi A. Kropp L. E. Schwartz D. J. Gorman G. H. Nylund C. M. (2018). Association between use of acid-suppressive medications and antibiotics during infancy and allergic diseases in early childhood. JAMA Pediatr. 172 (6), e180315. 10.1001/jamapediatrics.2018.0315 29610864 PMC6137535

[B161] Mizushina Y. Akihisa T. Ukiya M. Hamasaki Y. Murakami-Nakai C. Kuriyama I. (2005). Structural analysis of isosteviol and related compounds as DNA polymerase and DNA topoisomerase inhibitors. Life Sci. 77 (17), 2127–2140. 10.1016/j.lfs.2005.03.022 15935396

[B162] Mlambo R. Wang J. Chen C. (2022). Stevia rebaudiana, a versatile food ingredient: the chemical composition and medicinal properties. J. Nanomater. 2022 (1), 3573005. 10.1155/2022/3573005

[B163] Molina-Calle M. Priego-Capote F. Luque de Castro M. D. (2017). Characterization of stevia leaves by LC-QTOF MS/MS analysis of polar and non-polar extracts. Food Chem. 219, 329–338. 10.1016/j.foodchem.2016.09.148 27765234

[B164] Mora M. R. Dando R. (2021). The sensory properties and metabolic impact of natural and synthetic sweeteners. Compr. Rev. Food Sci. Food Saf. 20 (2), 1554–1583. 10.1111/1541-4337.12703 33580569

[B165] Moraes Neto R. N. Setúbal R. F. B. Higino T. M. M. Brelaz-de-Castro M. C. A. da Silva L. C. N. Aliança A. S. D.S. (2019). Asteraceae plants as sources of compounds against leishmaniasis and chagas disease. Front. Pharmacol. 10, 477. 10.3389/fphar.2019.00477 31156427 PMC6530400

[B166] Mousavi-Niri N. Khakpai F. Moheb-Alian M. Ghanimati E. Abdollah-Pour F. Naseroleslami M. (2023). Nano-stevia reduces the liver injury caused by streptozotocin (STZ)-Induced diabetes in rats by targeting PEPCK/GCK genes, INSR pathway and apoptosis. J. Diabetes Metab. Disord. 22 (2), 1519–1529. 10.1007/s40200-023-01278-2 37975120 PMC10638348

[B167] Muanda F. N. Soulimani R. Diop B. Dicko A. (2011). Study on chemical composition and biological activities of essential oil and extracts from Stevia rebaudiana bertoni leaves. LWT - Food Sci. Technol. 44 (9), 1865–1872. 10.1016/j.lwt.2010.12.002

[B168] Myint K. Z. Wu K. Xia Y. Fan Y. Shen J. Zhang P. (2020). Polyphenols from Stevia rebaudiana (bertoni) leaves and their functional properties. J. Food Sci. 85 (2), 240–248. 10.1111/1750-3841.15017 31990038

[B169] Myint K. Z. Zhou Z. Shi Q. Chen J. Dong X. Xia Y. (2023). Stevia polyphenols, their antimicrobial and anti-inflammatory properties, and inhibitory effect on digestive enzymes. Mol. 28 (22), 7572. 10.3390/molecules28227572 38005293 PMC10673113

[B170] Niu J. Xu L. Qian Y. Sun Z. Yu D. Huang J. (2020). Evolution of the gut microbiome in early childhood: a cross-sectional study of Chinese children. Front. Microbiol. 11, 439. 10.3389/fmicb.2020.00439 32346375 PMC7169428

[B171] Nunes A. P. Ferreira-Machado S. C. Nunes R. M. Dantas F. J. De Mattos J. C. Caldeira-de-Araújo A. (2007). Analysis of genotoxic potentiality of stevioside by comet assay. Food Chem. Toxicol. 45 (4), 662–666. 10.1016/j.fct.2006.10.015 17187912

[B172] Olas B. (2022). Stevia rebaudiana bertoni and its secondary metabolites: their effects on cardiovascular risk factors. Nutrition 99-100, 111655. 10.1016/j.nut.2022.111655 35588653

[B173] Onakpoya I. J. Heneghan C. J. (2015). Effect of the natural sweetener, steviol glycoside, on cardiovascular risk factors: a systematic review and meta-analysis of randomised clinical trials. Eur. J. Prev. Cardiol. 22 (12), 1575–1587. 10.1177/2047487314560663 25412840

[B174] Orellana-Paucar A. M. (2023). Steviol glycosides from stevia rebaudiana: an updated overview of their sweetening activity, pharmacological properties, and safety aspects. Molecules 28 (3), 1258. 10.3390/molecules28031258 36770924 PMC9920402

[B175] Ortega-Carballo K. J. Gil-Becerril K. M. Acosta-Virgen K. B. Casas-Grajales S. Muriel P. Tsutsumi V. (2024). Effect of stevioside (Stevia rebaudiana) on Entamoeba histolytica trophozoites. Pathogens 13 (5), 373. 10.3390/pathogens13050373 38787225 PMC11123825

[B176] Oudbor L. Mokhtari Z. Dastghaib S. Mokarram P. Rajani H. F. Barazesh M. (2022). Aqueous extract of Stevia rebaudiana (bertoni) bertoni abrogates death-related signaling pathways *via* boosting the expression profile of oxidative defense systems. J. Food Biochem. 46 (7), e14151. 10.1111/jfbc.14151 35365911

[B177] Padilla-Mayne S. Ovalle-Magallanes B. Figueroa M. Linares E. Bye R. Rivero-Cruz I. (2024). Chemical analysis and antidiabetic potential of a decoction from Stevia serrata roots. J. Nat. Prod. 87 (3), 501–513. 10.1021/acs.jnatprod.3c00711 37738100

[B178] Paiva L. S. Motta M. H. Baptista J. A. B. (2024). Nutraceutical value of eleven aromatic medicinal plants and azorean camellia sinensis: Comparison of antioxidant properties and phenolic and flavonoid contents. Processes 12 (7), 1375. 10.3390/pr12071375

[B179] Park S. Sethi S. Bouret S. G. (2019). Non-nutritive sweeteners induce hypothalamic ER stress causing abnormal axon outgrowth. Front. Endocrinol. (Lausanne) 10, 876. 10.3389/fendo.2019.00876 31920985 PMC6928131

[B180] Park M. Baek H. Han J. Y. Lee H. J. (2022a). Stevioside enhances the anti-adipogenic effect and β-Oxidation by activating AMPK in 3T3-L1 cells and epididymal adipose tissues of Db/Db mice. Cells 11 (7), 1076. 10.3390/cells11071076 35406641 PMC8997985

[B181] Park M. Sharma A. Baek H. Han J. Y. Yu J. Lee H. J. (2022b). Stevia and stevioside attenuate liver steatosis through PPARα-Mediated lipophagy in db/db mice hepatocytes. Antioxidants (Basel) 11 (12), 2496. 10.3390/antiox11122496 36552704 PMC9774531

[B182] Pasqualli T. Chaves P. E. E. Pereira C. Serpa É A. Oliveira L. F. S. Machado M. M. (2020). Steviol, the active principle of the stevia sweetener, causes a reduction of the cells of the immunological system even consumed in low concentrations. Immunopharmacol. Immunotoxicol. 42 (5), 504–508. 10.1080/08923973.2020.1811309 32811239

[B183] Paul S. Sengupta S. Bandyopadhyay T. Bhattacharyya A. (2012). Stevioside induced ROS-mediated apoptosis through mitochondrial pathway in human breast cancer cell line MCF-7. Nutr. cancer 64 (7), 1087–1094. 10.1080/01635581.2012.712735 23061910

[B184] Peteliuk V. Rybchuk L. Bayliak M. Storey K. B. Lushchak O. (2021). Natural sweetener Stevia rebaudiana: functionalities, health benefits and potential risks. Excli J. 20, 1412–1430. 10.17179/excli2021-4211 34803554 PMC8600158

[B185] Peters R. Burdock G. A. (2008). GRAS notification for the use of high-purity rebaudioside A (≥95%) as a sweetener in food.

[B186] Pinto J. Skjefte M. Alonso-Padilla J. Lozano Beltran D. F. Pinto L. V. Casellas A. (2023). Five-year serological and clinical evolution of chronic Chagas disease patients in Cochabamba, Bolivia. PLoS Negl. Trop. Dis. 17 (12), e0011498. 10.1371/journal.pntd.0011498 38157376 PMC10756508

[B187] Planas G. M. Kucacute J. (1968). Contraceptive properties of Stevia rebaudiana. Science 162 (3857), 1007. 10.1126/science.162.3857.1007 17744732

[B271] POWO (2025). Plants of the World Online. Facilitated by the Royal Botanic Gardens, Kew. Published on the Internet. Available online at: https://powo.science.kew.org/. (Accessed March 4, 2025).

[B188] Pradeep P. Thomas A. Kaur K. Sarah Samson R. Mayya A. Adiga S. (2024). Herbal medicines to prevent dental caries. Cochrane Database Syst. Rev. 5 (5). 10.1002/14651858.CD015832 39908071 PMC11091949

[B189] Prakash I. Ma G. Bunders C. Charan R. D. Ramirez C. Devkota K. P. (2017). A novel diterpene glycoside with nine glucose units from Stevia rebaudiana Bertoni. Biomolecules 7 (1), 10. 10.3390/biom7010010 28146121 PMC5372722

[B190] Prasitpuriprecha N. Santaweesuk S. Boonkert P. Chamnan P. (2022). Prevalence and DALYs of skin diseases in Ubonratchathani based on real-world national healthcare service data. Sci. Rep. 12 (1), 16931. 10.1038/s41598-022-20237-0 36209158 PMC9547855

[B191] Preethi R. Priyadharshini R. Selvaraj J. Palati S. (2023). Molecular mechanisms underlying chemopreventive anticancer activity of stevioside on human prostate cancer cell line *in vitro* . Clin. Cancer Investigation J. 12 (2), 8–11. 10.51847/lvXDmvXRty

[B192] Prithiksha N. Priyadharshini R. (2024). *In vitro* molecular mechanisms of anticancer activity of stevioside in human osteosarcoma cell lines (Sarcoma osteogenic). Contemp. Clin. Dent. 15 (3), 198–201. 10.4103/ccd.ccd_429_23 39512298 PMC11540208

[B193] Purkayastha S. Kwok D. (2020). Metabolic fate in adult and pediatric population of steviol glycosides produced from stevia leaf extract by different production technologies. Regul. Toxicol. Pharmacol. 116, 104727. 10.1016/j.yrtph.2020.104727 32745585

[B194] Raghavan G. Bapna A. Mehta A. Shah A. Vyas T. (2023). Effect of sugar replacement with Stevia-Based tabletop sweetener on weight and cardiometabolic health among Indian adults. Nutrients 15 (7), 1744. 10.3390/nu15071744 37049584 PMC10097272

[B195] Raghu A. Velayudhannair K. (2023). Phytochemical analysis and antibacterial potential of Stevia rebaudiana (Bertoni, 1899) leaf extracts against Aeromonas species: influence of extraction methods and solvents in aquaculture applications. J. Pure Appl. Microbiol. 17 (4), 2352–2366. 10.22207/jpam.17.4.31

[B196] Ragone M. I. Bonazzola P. Colareda G. A. Lazarte M. L. Bruno F. Consolini A. E. (2017). Cardioprotection of stevioside on stunned rat hearts: a mechano-energetical study. Phytomedicine 35, 18–26. 10.1016/j.phymed.2017.08.022 28991641

[B197] Rahman M. M. Uddin M. J. Reza A. S. M. A. Tareq A. M. Emran T. B. Simal-Gandara J. (2021). Ethnomedicinal value of antidiabetic plants in Bangladesh: a comprehensive review. Plants 10 (4), 729. 10.3390/plants10040729 33918026 PMC8070064

[B198] Ramesh K. Singh V. Megeji N. W. (2006). “Cultivation of Stevia [Stevia rebaudiana (Bert.) Bertoni]: A Comprehensive Review,” in Advances in agronomy (Academic Press), 137–177.

[B199] Ramos-Tovar E. Flores-Beltrán R. E. Galindo-Gómez S. Vera-Aguilar E. Diaz-Ruiz A. Montes S. (2018). Stevia rebaudiana tea prevents experimental cirrhosis *via* regulation of NF-κB, Nrf2, transforming growth factor beta, Smad7, and hepatic stellate cell activation. Phytother. Res. 32 (12), 2568–2576. 10.1002/ptr.6197 30251285

[B200] Ray J. Kumar S. Laor D. Shereen N. Nwamaghinna F. Thomson A. (2020). Effects of Stevia Rebaudiana on glucose homeostasis, blood pressure and inflammation: a critical review of past and Current research evidence. Int. J. Clin. Res. and Trials 5 (1). 10.15344/2456-8007/2020/142

[B201] Reutrakul S. Deerochanawong C. (2016). Diabetes in Thailand: status and Policy. Curr. Diab Rep. 16 (3), 28. 10.1007/s11892-016-0725-7 26894266

[B202] Rizwan F. Rashid H. U. Yesmine S. Monjur F. Chatterjee T. K. (2018). Preliminary analysis of the effect of Stevia (Stevia rebaudiana) in patients with chronic kidney disease (stage I to stage III). Contemp. Clin. Trials Commun. 12, 17–25. 10.1016/j.conctc.2018.08.007 30211340 PMC6129687

[B203] Rizwan F. Yesmine S. Banu S. G. Chowdhury I. A. Hasan R. Chatterjee T. K. (2019). Renoprotective effects of stevia (Stevia rebaudiana Bertoni), amlodipine, valsartan, and losartan in gentamycin-induced nephrotoxicity in the rat model: biochemical, hematological and histological approaches. Toxicol. Rep. 6, 683–691. 10.1016/j.toxrep.2019.07.003 31372346 PMC6656923

[B204] Rizwan F. Yesmine S. Chowdhury I. A. Banu S. G. Chatterjee T. K. (2020). Dataset concerning effects of stevia (Stevia rebaudiana bertoni), amlodipine, losartan, and valsartan on water consumption, blood glucose and heart tissue in gentamycin-induced nephrotoxicity in the rat model. Data Brief. 31, 105965. 10.1016/j.dib.2020.105965 32671162 PMC7347974

[B205] Rojas E. Bermúdez V. Motlaghzadeh Y. Mathew J. Fidilio E. Faria J. (2018). Stevia rebaudiana Bertoni and its effects in human disease: emphasizing its role in inflammation, atherosclerosis and metabolic syndrome. Curr. Nutr. Rep. 7, 161–170. 10.1007/s13668-018-0228-z 29995279

[B206] Ruiz-Ojeda F. J. Plaza-Díaz J. Sáez-Lara M. J. Gil A. (2019). Effects of sweeteners on the gut Microbiota: a review of experimental studies and clinical trials. Adv. Nutr. 10 (Suppl. l_1), S31–s48. 10.1093/advances/nmy037 30721958 PMC6363527

[B207] Ruiz-Ruiz J. C. Moguel-Ordoñez Y. B. Segura-Campos M. R. (2017). Biological activity of Stevia rebaudiana Bertoni and their relationship to health. Crit. Rev. Food Sci. Nutr. 57 (12), 2680–2690. 10.1080/10408398.2015.1072083 26479769

[B208] Saadi H. F. Zamani M. Koohpeyma F. Raeisi A. Amirahmadi Z. Rezaei N. (2024). Therapeutic potential of aquatic Stevia extract in alleviating endoplasmic reticulum stress and liver damage in streptozotocin-induced diabetic rats. Mol. Biol. Rep. 51 (1), 993. 10.1007/s11033-024-09907-6 39292293

[B209] Salehi B. López M. D. Martínez-López S. Victoriano M. Sharifi-Rad J. Martorell M. (2019). Stevia rebaudiana Bertoni bioactive effects: from *in vivo* to clinical trials towards future therapeutic approaches. Phytother. Res. 33 (11), 2904–2917. 10.1002/ptr.6478 31423662

[B210] Salinas-Velarde I. D. Bernal-Morales B. Pacheco-Cabrera P. Sánchez-Aparicio P. Pascual-Mathey L. I. Venebra-Muñoz A. (2021). Lower ΔFosB expression in the dopaminergic system after stevia consumption in rats housed under environmental enrichment conditions. Brain Res. Bull. 177, 172–180. 10.1016/j.brainresbull.2021.10.001 34624462

[B211] Samuel P. Ayoob K. T. Magnuson B. A. Wölwer-Rieck U. Jeppesen P. B. Rogers P. J. (2018). Stevia leaf to stevia sweetener: exploring its science, benefits, and future potential. J. Nutr. 148 (7), 1186S–1205S. 10.1093/jn/nxy102 29982648

[B212] Shahu R. Kumar D. Ali A. Tungare K. Al-Anazi K. M. Farah M. A. (2023). Unlocking the therapeutic potential of Stevia rebaudiana Bertoni: a natural antiglycating agent and non-toxic support for HDF cell health. Molecules 28 (19), 6797. 10.3390/molecules28196797 37836640 PMC10574660

[B213] Sharipova R. R. Belenok M. G. Garifullin B. F. Sapunova A. S. Voloshina A. D. Andreeva O. V. (2019). Synthesis and anti-cancer activities of glycosides and glycoconjugates of diterpenoid isosteviol. Medchemcomm 10 (8), 1488–1498. 10.1039/c9md00242a 31673312 PMC6786240

[B214] Sharma S. K. Alam A. (2024). Ethnobotanical importance of families Apocynaceae, Asteraceae, and Fabaceae (Angiosperms) among Rajasthan tribes, India. Plant Sci. Today 11 (sp1). 10.14719/pst.3354

[B215] Shen T. Li J. (2021). Drinking non-nutritive sweetness solution of sodium saccharin or rebaudioside a for Guinea pigs: influence on histologic change and expression of sweet taste receptors in Testis and Epididymis. Front. Nutr. 8, 720889. 10.3389/fnut.2021.720889 34422887 PMC8375269

[B216] Shen W. Huang H. Xue J. Xie M.-L. (2022). Stevioside inhibits lipopolysaccharide-induced epithelial-to-mesenchymal transition of NRK-52E cells by PPARγ activation. Immunopharmacol. Immunotoxicol. 44 (2), 287–294. 10.1080/08923973.2022.2039935 35139741

[B217] Shiozaki K. Fujii A. Nakano T. Yamaguchi T. Sato M. (2006). Inhibitory effects of hot water extract of the Stevia stem on the contractile response of the smooth muscle of the Guinea pig ileum. Biosci. Biotechnol. Biochem. 70 (2), 489–494. 10.1271/bbb.70.489 16495667

[B218] Shivanna N. Naika M. Khanum F. Kaul V. K. (2013). Antioxidant, anti-diabetic and renal protective properties of Stevia rebaudiana. J. Diabetes Complicat. 27 (2), 103–113. 10.1016/j.jdiacomp.2012.10.001 23140911

[B219] Siddique A. B. Mizanur Rahman S. M. Hossain M. A. (2016). Chemical composition of essential oil by different extraction methods and fatty acid analysis of the leaves of Stevia Rebaudiana Bertoni. Arabian J. Chem. 9, S1185–S1189. 10.1016/j.arabjc.2012.01.004

[B220] Simlat M. Ptak A. Wójtowicz T. Szewczyk A. (2023). The content of phenolic compounds in Stevia rebaudiana (Bertoni) plants derived from melatonin and NaCl treated seeds. Plants (Basel) 12 (4), 780. 10.3390/plants12040780 36840128 PMC9960086

[B221] Simoni S. Vangelisti A. Clemente C. Usai G. Santin M. Ventimiglia M. (2024). Transcriptomic analyses reveal insights into the shared regulatory network of phenolic compounds and steviol glycosides in Stevia rebaudiana. Int. J. Mol. Sci. 25 (4), 2136. 10.3390/ijms25042136 38396813 PMC10889303

[B222] Singh A. Singh J. Parween G. Khator R. Monga V. (2024a). A comprehensive review of apigenin a dietary flavonoid: biological sources, nutraceutical prospects, chemistry and pharmacological insights and health benefits. Crit. Rev. Food Sci. Nutr. 65, 4529–4565. 10.1080/10408398.2024.2390550 39154213

[B223] Singh R. Borlace G. N. Sringam P. Thongkham E. Aiemsaard J. (2024b). Phytochemical composition and antimicrobial potential of Stevia rebaudiana Bertoni extract and its topical spray formulation against animal skin pathogens. Veterinary World 17, 2975–2984. 10.14202/vetworld.2024.2975-2984 39897348 PMC11784044

[B224] Slykerman R. F. Neumann D. Underwood L. Hobbs M. Waldie K. E. (2023). Age at first exposure to antibiotics and neurodevelopmental outcomes in childhood. Psychopharmacol. Berl. 240 (5), 1143–1150. 10.1007/s00213-023-06351-5 36930273 PMC10101895

[B225] Small E. Catling P. M. Daubeny H. (2001). BLOSSOMING TREASURES OF BIODIVERSITY: 1. Stevia (Stevia rebaudiana (Bertoni) Bertoni)—How sweet it is!. Biodiversity 2 (2), 22–23. 10.1080/14888386.2001.9712544

[B226] Soejarto D. D. Kinghorn A. D. Farnsworth N. R. (1982). Potential sweetening agents of plant origin. III. Organoleptic evaluation of Stevia leaf herbarium samples for sweetness. J. Nat. Prod. 45 (5), 590–599. 10.1021/np50023a013 7153776

[B227] Stachurska X. Mizielińska M. Ordon M. Nawrotek P. (2023). The use of plant extracts and bacteriophages as an alternative therapy approach in combatting bacterial infections: the study of lytic phages and Stevia rebaudiana. J. Vet. Res. 67 (4), 545–557. 10.2478/jvetres-2023-0059 38130461 PMC10730388

[B228] Stamataki N. S. Crooks B. Ahmed A. McLaughlin J. T. (2020). Effects of the daily consumption of stevia on glucose homeostasis, body weight, and energy intake: a randomised open-label 12-Week trial in healthy adults. Nutrients 12 (10), 3049. 10.3390/nu12103049 33036155 PMC7600789

[B229] Sudhakar K. Mishra V. Hemani V. Verma A. Jain A. Jain S. (2021). Reverse pharmacology of phytoconstituents of food and plant in the management of diabetes: current status and perspectives. Trends Food Sci. and Technol. 110, 594–610. 10.1016/j.tifs.2020.10.024

[B230] Sumit G. Subudhi E. Nayak S. (2008). Antimicrobial assay of Stevia rebaudiana Bertoni leaf extracts against 10 pathogens. Int. J. Integr. Biol. 2 (1), 27–31. Available online at: https://api.semanticscholar.org/CorpusID:85810578.

[B231] Suttajit M. Vinitketkaumnuen U. Meevatee U. Buddhasukh D. (1993). Mutagenicity and human chromosomal effect of stevioside, a sweetener from Stevia rebaudiana Bertoni. Environ. Health Perspect. 101 (Suppl. 3), 53–56. 10.1289/ehp.93101s353 8143647 PMC1521159

[B232] Takahashi K. Matsuda M. Ohashi K. Taniguchi K. Nakagomi O. Abe Y. (2001). Analysis of anti-rotavirus activity of extract from Stevia rebaudiana. Antivir. Res. 49 (1), 15–24. 10.1016/s0166-3542(00)00134-0 11166857

[B233] Taylor L. (2005). The healing power of rainforest herbs: a guide to understanding and using herbal medicinals. Square one. Gard. City Park, N. Y., 345. Available online at: https://www.amazon.com/Healing-Power-Rainforest-Herbs-Understanding/dp/0757001440.

[B234] Tipduangta P. Julsrigival J. Chaithatwatthana K. Pongterdsak N. Tipduangta P. Chansakaow S. (2019). Antioxidant properties of Thai traditional herbal teas. Beverages 5 (3), 44. 10.3390/beverages5030044

[B235] Tomita T. Sato N. Arai T. Shiraishi H. Sato M. Takeuchi M. (1997). Bactericidal activity of a fermented hot-water extract from Stevia rebaudiana Bertoni towards enterohemorrhagic Escherichia coli O157:H7 and other food-borne pathogenic bacteria. Microbiol. Immunol. 41 (12), 1005–1009. 10.1111/j.1348-0421.1997.tb01961.x 9492187

[B236] Toskulkao C. Deechakawan W. Temcharoen P. Buddhasukh D. Glinsukon T. (1994). Nephrotoxic effects of stevioside and steviol in rat renal cortical slices. J. Clin. Biochem. Nutr. 16 (2), 123–131. 10.3164/jcbn.16.123

[B237] Uçar A. Yılmaz S. Yılmaz Ş. Kılıç M. S. (2018). A research on the genotoxicity of stevia in human lymphocytes. Drug Chem. Toxicol. 41 (2), 221–224. 10.1080/01480545.2017.1349135 28738695

[B238] Ukiya M. Sawada S. Kikuchi T. Kushi Y. Fukatsu M. Akihisa T. (2013). Cytotoxic and apoptosis‐inducing activities of steviol and isosteviol derivatives against human cancer cell lines. Chem. and Biodivers. 10 (2), 177–188. 10.1002/cbdv.201200406 23418165

[B239] Urban J. D. Carakostas M. C. Taylor S. L. (2015). Steviol glycoside safety: are highly purified steviol glycoside sweeteners food allergens? Food Chem. Toxicol. 75, 71–78. 10.1016/j.fct.2014.11.011 25449199

[B240] Urrutia-Espinosa M. Concha-Fuentealba F. Fuentes-Barría H. Angarita Dávila L. C. Carrasco Hernández M. E. Aguilera-Eguía R. (2024). Effects of D-tagatose, Stevia and Sucrose on pH and oral bacterial activity in dentistry students. A randomized controlled trial. Nutr. Hosp. 41 (5), 1091–1097. 10.20960/nh.05253 39037177

[B241] Uyanikgil Y. Cavusoglu T. Balcıoglu H. A. Gurgul S. Solmaz V. Ozlece H. K. (2016). Rebaudioside A inhibits pentylenetetrazol-induced convulsions in rats. Kaohsiung J. Med. Sci. 32 (9), 446–451. 10.1016/j.kjms.2016.07.002 27638403 PMC11916012

[B242] Villegas Vílchez L. F. Ascencios J. H. Dooley T. P. (2022). GlucoMedix®, an extract of Stevia rebaudiana and Uncaria tomentosa, reduces hyperglycemia, hyperlipidemia, and hypertension in rat models without toxicity: a treatment for metabolic syndrome. BMC Complement. Med. Ther. 22 (1), 62. 10.1186/s12906-022-03538-9 35260150 PMC8905912

[B243] Wang D. (2000). Zhonghua Bencao (in Chinese). Beijing: Zhongyi Yao Chubanshe.

[B244] Wang L. Wu W. (2019). Angiotensin-converting enzyme inhibiting ability of ethanol extracts, steviol glycosides and protein hydrolysates from stevia leaves. Food Funct. 10 (12), 7967–7972. 10.1039/c9fo02127b 31750488

[B245] Wang Y. Li L. Wang Y. Zhu X. Jiang M. Song E. (2018). New application of the commercial sweetener rebaudioside a as a hepatoprotective candidate: induction of the Nrf2 signaling pathway. Eur. J. Pharmacol. 822, 128–137. 10.1016/j.ejphar.2018.01.020 29355553

[B246] Wang Y. Zhu S. Wang S. (2023). Treatment of Mycoplasma pneumonia in children from the perspective of wind-warm phlegm-heat. J. Nanjing Univ. Tradit. Chin. Med. 39 (12), 1237–1241. (in Chinese). 10.14148/j.issn.1672-0482.2023.1237

[B247] Wanyo P. Chomnawang C. Huaisan K. Chamsai T. (2024). Comprehensive analysis of antioxidant and phenolic profiles of Thai medicinal plants for functional food and pharmaceutical development. Plant Foods Hum. Nutr. 79 (2), 394–400. 10.1007/s11130-024-01179-6 38668915

[B248] Waris A. Ullah A. Asim M. Ullah R. Rajdoula M. R. Bello S. T. (2024). Phytotherapeutic options for the treatment of epilepsy: pharmacology, targets, and mechanism of action. Front. Pharmacol. 15, 1403232. 10.3389/fphar.2024.1403232 38855752 PMC11160429

[B249] WHO (2015). Guideline: sugars intake for adults and children. Geneva: World Health Organization. 25905159

[B250] WHO (2022a). Sugars factsheet. Available online at: https://www.who.int/andorra/publications/m/item/sugars-factsheet (Accessed October 21, 2024).

[B251] WHO (2022b). WHO guidelines on non-sugar sweeteners: a health recommendation. Geneva, Switzerland: World Health Organization.

[B252] Wölwer-Rieck U. (2012). The leaves of Stevia rebaudiana (Bertoni), their constituents and the analyses thereof: a review. J. Agric. Food Chem. 60 (4), 886–895. 10.1021/jf2044907 22250765

[B253] Wu X. Zhao Y. Shi W. Lu L. Chen G. Wu J. (2021). Toxicological safety evaluation of stevioside M (in Chinese). Mod. Food Sci. Technol. 37 (03), 250–258. 10.13982/j.mfst.1673-9078.2021.3.0760

[B254] Wu M. Wang Y. Sun X. Sun L. (2025). Research on the design space of extraction process for Folium stevia formula granules based on consistency with standard decoction(in Chinese). Front. Pharm. Sci. 29 (01), 40–49.Available online at: https://kns.cnki.net/kcms2/article/abstract?v=a4fp6zKrpgY-V37lKf9dDYNwPfGgNPsouE6xaXNwMdnruooRoq2A3fZaI4LWKey-4oSHSOLt-BWOHeHxa-YNSRWF2z43TAkaP9IGH3uFfwJH5zbkzX1mWKxel_2Hsu3Nj22_wkJRygyZ5iPJPBLTJAFxzaBjioka9GcSwcTUFxtbzFPMek3kpfIGldjX1Rt0jGVhhNDjJIE=&uniplatform=NZKPT&language=CHS.

[B255] Xu L. Zhao Z. (2023). Protective effects and mechanisms of Xiao'er Pingchuan formula on lung function in children with cough variant asthma. (in Chinese). Chin. J. Chin. Mater Med. 46 (06), 1552–1556. 10.13863/j.issn1001-4454.2023.06.039

[B256] Xu Q. Liu M. Chao X. Zhang C. Yang H. Chen J. (2023). Stevioside improves antioxidant capacity and intestinal barrier function while attenuating inflammation and apoptosis by regulating the NF-κB/MAPK pathways in diquat-induced oxidative stress of IPEC-J2 cells. Antioxidants (Basel) 12 (5), 1070. 10.3390/antiox12051070 37237936 PMC10215602

[B257] Yang J. Liu F. Wang F. (2024). Treatment of recurrent respiratory infections with spleen deficiency and accumulation syndrome based on the “simultaneous elimination and supplementation” approach. (in Chinese). J. Pract. Tradit. Chin. Intern Med., 1–5. Available online at: https://link.cnki.net/urlid/21.1187.R.20240518.1458.002.

[B258] Yao Y.-X. Yu Y.-J. Dai S. Zhang C.-Y. Xue X.-Y. Zhou M.-L. (2024). Kaempferol efficacy in metabolic diseases: molecular mechanisms of action in diabetes mellitus, obesity, non-alcoholic fatty liver disease, steatohepatitis, and atherosclerosis. Biomed. and Pharmacother. 175, 116694. 10.1016/j.biopha.2024.116694 38713943

[B259] Yeung A. W. K. (2023). Bibliometric analysis on the literature of monk fruit extract and mogrosides as sweeteners. Front. Nutr. 10, 1253255. 10.3389/fnut.2023.1253255 37706210 PMC10495570

[B260] Yi T. Zhao Z. Z. Yu Z. L. Chen H. B. (2010). Comparison of the anti-inflammatory and anti-nociceptive effects of three medicinal plants known as “Snow Lotus” herb in traditional Uighur and Tibetan medicines. J. Ethnopharmacol. 128 (2), 405–411. 10.1016/j.jep.2010.01.037 20083181

[B261] Yi T. Lo H. Zhao Z. Yu Z. Yang Z. Chen H. (2012). Comparison of the chemical composition and pharmacological effects of the aqueous and ethanolic extracts from a Tibetan “Snow Lotus” (Saussurea laniceps) herb. Molecules 17 (6), 7183–7194. 10.3390/molecules17067183 22692242 PMC6269069

[B262] Younes M. Aquilina G. Engel K. H. Fowler P. Frutos Fernandez M. J. Fürst P. (2020). Safety of a proposed amendment of the specifications for steviol glycosides (E 960) as a food additive: to expand the list of steviol glycosides to all those identified in the leaves of Stevia Rebaudiana Bertoni. Efsa J. 18 (4), e06106. 10.2903/j.efsa.2020.6106 32874306 PMC7448073

[B263] Zeng Y. Liang J. Q. (2022). Nasal microbiome and its interaction with the host in childhood asthma. Cells 11 (19), 3155. 10.3390/cells11193155 36231116 PMC9563732

[B264] Zhang Q. Yang H. Li Y. Liu H. Jia X. (2017). Toxicological evaluation of ethanolic extract from Stevia rebaudiana Bertoni leaves: genotoxicity and subchronic oral toxicity. Regul. Toxicol. Pharmacol. 86, 253–259. 10.1016/j.yrtph.2017.03.021 28351677

[B265] Zhang Y. Xu S. Jin Y. Dai Y. Chen Y. Wu X. (2020). Efficient biocatalytic preparation of rebaudioside KA: highly selective glycosylation coupled with UDPG regeneration. Sci. Rep. 10 (1), 6230. 10.1038/s41598-020-63379-9 32277148 PMC7148340

[B266] Zhao R. Wang J. Qin L. Zhang X. Mei Y. (2020). Stevioside improved hyperglycemia-induced cardiac dysfunction by attenuating the development of fibrosis and promoting the degradation of established fibrosis. J. Funct. Foods 68, 103895. 10.1016/j.jff.2020.103895

[B267] Zhou X. Gong M. Lv X. Liu Y. Li J. Du G. (2021). Metabolic engineering for the synthesis of steviol glycosides: current status and future prospects. Appl. Microbiol. Biotechnol. 105 (13), 5367–5381. 10.1007/s00253-021-11419-3 34196745

[B268] Zhu M. Sun Y. Su Y. Guan W. Wang Y. Han J. (2024). Luteolin: a promising multifunctional natural flavonoid for human diseases. Phytother. Res. 38 (7), 3417–3443. 10.1002/ptr.8217 38666435

[B269] Zipinotti Dos Santos D. de Souza J. C. Pimenta T. M. da Silva Martins B. Junior R. S. R. Butzene S. M. S. (2023). The impact of lipid metabolism on breast cancer: a review about its role in tumorigenesis and immune escape. Cell Commun. Signal 21 (1), 161. 10.1186/s12964-023-01178-1 37370164 PMC10304265

[B270] Zou X. Tan Q. Goh B.-H. Lee L.-H. Tan K.-L. Ser H.-L. (2020). ‘Sweeter’than its name: anti-inflammatory activities of Stevia rebaudiana. All Life 13 (1), 286–309. 10.1080/26895293.2020.1771434

